# “Small is beautiful” – Examining reliable determination of low-abundant therapeutic antibody glycovariants

**DOI:** 10.1016/j.jpha.2024.100982

**Published:** 2024-04-26

**Authors:** Katharina Böttinger, Christof Regl, Veronika Schäpertöns, Erdmann Rapp, Therese Wohlschlager, Christian G. Huber

**Affiliations:** a Department of Biosciences and Medical Biology, Bioanalytical Research Labs, University of Salzburg, Salzburg, 5020, Austria; b Center for Tumorbiology and Immunology (CTBI), University of Salzburg, Salzburg, 5020, Austria; c glyXera GmbH, Magdeburg, Sachsen-Anhalt, 39014, Germany; d Max Planck Institute for Dynamics of Complex Technical Systems, Magdeburg, 39106, Germany

**Keywords:** Glycosylation, Mass spectrometry, Monoclonal antibodies, Abundance profiling, Minor glycovariants, Multi-level analysis, Data integration

## Abstract

Glycans associated with biopharmaceutical drugs play crucial roles in drug safety and efficacy, and therefore, their reliable detection and quantification is essential. Our study introduces a multi-level quantification approach for glycosylation analysis in monoclonal antibodies (mAbs), focusing on minor abundant glycovariants. Mass spectrometric data is evaluated mainly employing open-source software tools. Released *N-*glycan and glycopeptide data form the basis for integrating information across different structural levels up to intact glycoproteins. Comprehensive comparison showed that indeed, variations across structural levels were observed especially for minor abundant species. Utilizing modification finder (MoFi), a tool for annotating mass spectra of intact proteins, we quantify isobaric glycosylation variants at the intact protein level. Our workflow's utility is demonstrated on NISTmAb, rituximab and adalimumab, profiling their minor abundant variants for the first time across diverse structural levels. This study enhances understanding and accessibility in glycosylation analysis, spotlighting minor abundant glycovariants in therapeutic antibodies.

## Highlights

• Quantification of minor abundant glycovariants occurring in monoclonal antibodies.
• Integration of quantitative data across protein structural levels.
• Quantification of MS data at the raw data level by open-source software tools.
• Site-specific discrepancies exist at diverse structural levels for minor species.
• Comprehensive meta-data study on glycosylation analyses in literature.

## Graphical abstract

**Figure:**
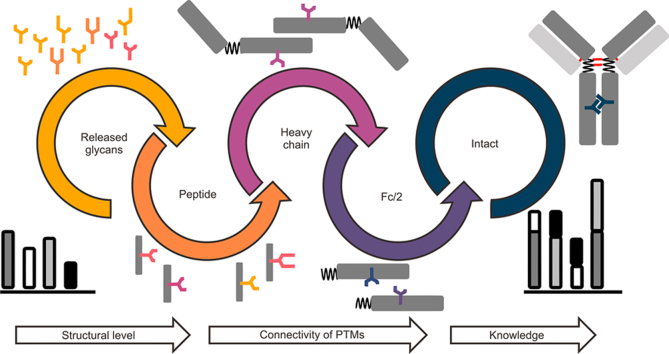

## 1 Introduction

Cells have multiple ways to modify the primary protein sequence of a secreted protein by attaching mono- or oligo-saccharides. Such mechanisms enable them to fine-tune the resulting glycoprotein's structure and function. The attachment of glycans to glycosylation sites is a non-template-driven enzymatic process, which results in a natural heterogeneity of glycoproteins [1]. As a result, a single, cell-secreted glycoprotein may embody a pool of hundreds of different glycoform species [2,3]. The molecular mass of glycosylated monoclonal antibodies (mAbs), such as immunoglobulin G (IgG)-type mAbs, amounts to around 150 kDa of which typically 1%−5% account for the carbohydrate portion, predominantly *N-*linked glycans [4].

The monosaccharide composition and linkages, as well as abundance of glycans wield substantial influence over the efficacy and safety of biotherapeutics. Among these factors, *N-*linked glycosylation stands as a pivotal critical quality attribute (CQA) for biotherapeutics, shaping their effector functions [1]. Core afucosylation enhances antibody-dependent cellular cytotoxicity (ADCC), while terminal galactosylation supports complement-dependent cytotoxicity (CDC) by aiding C1q binding [5]. Terminal sialylation contributes to anti-inflammatory effects [6], and the binding affinity to Fcγ-receptors varies based on terminal *N-*acetylneuraminic acid linkage [7]. In the pharmaceutical industry, quantitative alterations in CQAs are subject to approval, provided they do not compromise the biotherapeutic's safety and efficacy [2,8]. Notably, the ICH Q6B guideline outlines three key considerations for carbohydrate characterization on the protein: (i) carbohydrate content, (ii) carbohydrate structure, and (iii) carbohydrate location [9].

Protein glycosylation can be analysed at different structural levels, such as released glycans, glycopeptides, protein subunit domains (Fc/2 and heavy chain), and intact glycoproteins [[10], [11], [12]]. Mass spectrometry (MS) represents one of the preferred methods for studying protein glycosylation, offering quantitative data based on mass spectrometric signal intensities. Intact glycoprotein analysis by native mass spectrometry (nMS) provides a quick snapshot of protein variants and post-translational modifications (PTMs) with minimal sample preparation, but it has limited glycoform resolution due to natural isotopologue patterns [3,11]. Moreover, mass spectrometric analysis of intact glycoproteins cannot differentiate *N-*glycan compositions of equal or similar mass [13]. In contrast, subunit and glycopeptide analysis provides quantitative data on individual glycans, while released glycan analysis represents the gold standard for quantification, independent of glycan structure and capable of determining glycan linkages [10]. A recent study by Carillo et al. [10], in which individual methods for *N-*glycan quantification at diverse structural levels were applied, highlights the challenges in quantification of minor abundant glycosylation variants. This study showed that while obtaining glycosylation profiles of the major glycan species is relatively straightforward, quantification of minor species is highly method dependent [10]. Furthermore, diverse inter-laboratory studies emphasize the need for robust quantification methods, especially in addressing challenges associated with less prevalent glycoforms [[14], [15], [16]].

Chemical heterogeneity poses challenges to glycoprotein quantification, including sample preparation artifacts, glycovariant separation, and isomer issues in MS [16]. In addition, quantitative glycan profiles can be affected by various factors, including data processing and evaluation. Bioinformatic tools are available for relative glycosylation profiling, where mass-to-charge spectra of multiply charged protein species are converted into zero-charge spectra for quantification [10]. Ideally, the deconvoluted spectrum mimics the raw spectrum. Nonetheless, it is important to carefully set deconvolution parameters to avoid biased results, but strict settings may lead to information loss or false masses [17,18]. Thus, we previously emphasized the direct quantification of glycosylation variants from raw spectra [19]. In this respect, the R-based package fragquaxi can be applied to data of intact proteins and protein subunits, omitting the use of a deconvolution software [19,20]. Glycopeptides can be readily quantified from raw spectra using the freely available software tool Skyline [21]. These alternatives offer more accurate results as they bypass the need for a prior deconvolution step [19].

Motivated by these premises, we focused on a comprehensive workflow to relatively quantify less abundant glycosylation species in mAbs. Our approach utilizes readily available software tools such as Skyline for glycopeptide quantification and the R-package fragquaxi for subunit and intact glycoprotein profiling. Furthermore, we employ an algorithm to correct glycosylation profiles for glycation bias (CAFOG) [22]. This user-friendly workflow enables the quantification of minor abundant glycoprotein species as demonstrated for different mAbs. In this study, we address the analytical challenges posed by the structural diversity of glycan structures in characterizing and quantifying mAbs and their glycan variants. Through chemical and enzymatic treatments, we extensively characterize *N-*glycan species at multiple structural levels, including released *N-*glycan, glycopeptide, subunit, and intact protein levels. Additionally, to assess the detectability of low-abundant glycans in mAbs, we employed multiplexed capillary gel electrophoresis with laser-induced fluorescence detection (xCGE-LIF) as a benchmark technology, adding an extra dimension to the novelty of our study.

In light of a recent multi-interlaboratory study on antibody glycosylation, which highlighted challenges in confidently identifying and quantifying less abundant glycan structures [16], our study introduces several novel aspects: 1) We concentrate on profiling the less abundant glycosylation species in three different mAbs. 2) Unlike a comparative approach of different methods, our study emphasizes the integration of quantitative data across lower to higher structural levels, providing a comprehensive view of glycosylation. 3) Our methodology significantly reduces reliance on commercially available MS-data evaluation software, enhancing the accessibility and versatility of our approach.

## 2 Materials and methods

### 2.1 Chemicals and materials

The antibodies, rituximab (MabThera, batch N7025B04, exp. 02/17) and adalimumab (Humira, batch 1030241, exp. 10/16) were purchased from a local pharmacy store and stored at +7 °C. NISTmAb (RM8671, batch 14 HB-D-002) was obtained from the National Institute of Standards and Technology (NIST) Material Measurement Laboratory (Gaithersburg, MD, USA) and stored at −20 °C. Carboxypeptidase B (CpB) was obtained from Roche (Mannheim, Germany), PNGase F (2500 units, NEB P0705S, PNGase F Glycerol-free) was obtained from New England BioLabs GmbH (Frankfurt am Main, Germany). Sequencing grade trypsin (porcine) was bought from Promega (Madison, WI, USA). Ultrapure water was produced in-house using a water purification system (Milli-Q Integral 3, Merck/Millipore, Billerica, MA, USA). Ammonium hexa-fluorophosphate (AHFP, 99.99%), formic acid (FA, 98.0%–100.0%), guanidine hydrochloride (Gnd-HCl), iodoacetamide (IAA, ≥99.0%) and tris(2-carboxyethyl)phosphine (TCEP, ≥98.0%) were obtained from Sigma-Aldrich (St. Louis, MO, USA). High-performance liquid chromatography-mass spectrometry (HPLC-MS) grade acetonitrile (ACN) and methanol (MeOH) were purchased from VWR International (Radnor, PA, USA). Ammonium acetate (AmAc, ≥98.0%) was acquired from Merck (Darmstadt, Germany).

30 kDa and 50 kDa molecular weight cut-off (MWCO) filters were purchased from Sartorius Vivaspin (Göttingen, Germany). The immobilized IdeS (IgG degrading enzyme of *Streptococcus pyogenes*) columns were ordered from Genovis AB (Lund, Sweden). C_18_ purification tips (Pierce™ C18 Spin Tips) were purchased from Thermo Scientific™ (Rockford, IL, USA). S-Trap mini columns were obtained from Protifi (Huntington, NY, USA).

### 2.2 Released glycan analysis

The workflow of released glycan analysis was performed by glyXera GmbH and is described elsewhere [20,23,24].

### 2.3 Glycopeptide analysis

Tryptic peptides were generated following the S-Trap protocol (mini) according to manufacturer's suggestions with minor adaptions: for alkylation, 500 mmol/L IAA was used. For enzymatic deglycosylation, 10 μg (in a volume of 100 μL) of rituximab and adalimumab were mixed with 10 μL of 10x glycerol free buffer and 10 μL of PNGase F. 10 μg (in a volume of 50 μL) of NISTmAb were mixed with 5.0 μL of 10x glycerol free buffer and 5.0 μL of PNGase F. Digestion time was 24 h at 37 °C. In pre-experiments, this workflow has proven superior over a classical workflow containing C_18_ ZipTip purification (Fig. S1).

#### 2.3.1 HPLC-MS set-up and parameters for glycopeptide analysis

Separation was carried out on a Double nanoViper™ PepMap™ Neo UHPLC Column (150 mm × 75 μm i.d., 2 μm d_p_, 100 Å pore size, C_18_, Thermo Scientific™, Vilnius, Lithuania) using HPLC-grade water with 0.10% FA and ACN with 0.10% FA as mobile phases A and B, respectively, with a flow rate of 300 nL/min. Separations were carried out at 50 °C ± 2 °C. Injection mode was "μL-PickUp"; 300 ng of trypsin digested mAb were loaded for analysis. After an equilibration phase for 5.0 min at 1.0% B, two linear gradients followed with a first increase to 30% B within 35.0 min, and a second increase to 60% B within 10.0 min. The column was washed with 99% B for 5.0 min and re-equilibrated at 1.0% B for 20.0 min. For mass spectrometric analysis, a Q-Exactive™ Pluss mass spectrometer (Thermo Scientific™, Bremen, Germany) was employed. Peptides were sprayed on a nanospray interface at +1.4 kV with zero sheath, sweep, and auxiliary gases, a capillary temperature of 250 °C, and an S-lens RF level of 60. A full scan was carried out from 400 to 3,000 *m/z* at a resolution setting of 70,000, with an automatic gain control (AGC) target of 3×10^6^ and a maximum injection time of 100 ms. Fragmentation was carried out in data-dependent mode. Top ten intense peptides were selected for fragmentation at a normalized collision energy of 28. Fragments were acquired at a resolution setting of 17,500, an AGC target of 1 × 10^5^, and maximum injection time of 150 ms. The scan range was set from 200 to 2,000 *m/z*. Each sample was analysed in technical quintuplicates.

### 2.4 Subunit and intact protein analysis

#### 2.4.1 CpB and PNGase F digests for subunit and intact analyses

Rituximab, adalimumab, and NISTmAb were digested with CpB using an enzyme: protein ratio of 1:5 (*w/w*) at 37 °C for 1.0 h at 1,000 rpm in 175 mmol/L AmAc. Glycans were removed using PNGase F at 37 °C for 17 h (200 μL antibody solution were mixed with 5.0 μL PNGase F). CpB and PNGase F digests were purified with 30 kDa MWCO filters using 175 mmol/L AmAc. The final mAb concentration was determined using nanodrop at 280 nm. These samples served as basis for subunit and intact protein measurements.

For disulfide bond reduction, mAbs were diluted to a concentration of 0.10 μg/μL in 4.0 mol/L guanidinium hydrochloride and 5.0 mmol/L TCEP. The reaction was allowed to proceed at 60 °C and 1,000 rpm for 15 min.

For IdeS digestion, 50 μg of mAb were diluted to a concentration of 0.20 μg/μL in 175 mmol/L AmAc and digested on an immobilized IdeS column according to manufacturer's instructions. Obtained IdeS digests were diluted to a final concentration of 0.10 μg/μL with 20% ACN.

#### 2.4.2 HPLC-MS set-up for subunit and intact protein measurement

The following system was utilized for analysis of reduced, IdeS digested, and intact mAb samples: An Ultimate 3000 instrument was equipped with a MAbPAC RP column (50 mm × 2.1 mm i.d., 4 μm d_p_) from Thermo Scientific™ (Vilnius, Lithuania). Eluent A was H_2_O with 0.10% FA. Eluent B was ACN with 0.10% FA. The column oven temperature was set to 70 °C, and sampler temperature to 4.0 °C. All samples were detected at 214 nm. Additionally, the HPLC was hyphenated to a Q-Exactive™ Plus mass spectrometer. All samples were sprayed at 3.5 kV, where flow rates were set to 25, 0, and 5 for sheath, sweep and aux gas, respectively. Capillary temperature was 300 °C and aux gas temperature was 150 °C. S-lens RF level was 100. Data acquisition was done using Chromeleon software (Thermo Scientific™).

#### 2.4.3 HPLC-MS settings for intact mAb analysis

The column was equilibrated at 10% B for 30 s, next followed a linear increase to 80% B for 3.0 min. The column was washed at 80% B for 1.5 min and re-equilibrated at 10% B for 5.0 min. Gradient elution was operated at a constant flow rate of 250 μL/min. MS acquisition settings are described in Table 1.

**Table 1:** Mass spectroscopy acquisition settings for glycosylation study.

Parameter	Intact	Reduced LC	Reduced HC	IdeS Fc/2	IdeS F(ab')2
Runtime (min)	0–10 min	0–9 min	9–15 min	0–8.5 min	8.5–15 min
Polarity	Positive	Positive	Positive	Positive	Positive
In-source CID (eV)	80.0	0	50.0	0	20
Microscans	10	10	10	10	10
Resolution at *m/z* 200	17,500	140,000	17,500	140,000	17,500
AGC target	3 × 10^6^	3 × 10^6^	3 × 10^6^	3 × 10^6^	3 × 10^6^
Maximum IT (ms)	200	200	200	200	200
Scan range (*m/z*)	1800–5000	1200–2400	1200–2400	900–2800	900–2800

LC: light chain; HC: heavy chain; IdeS: *I*gG *d*egrading *e*nzyme of *Streptococcus pyogenes*; CID: collision-induced dissociation; AGC: automatic gain control; IT: injection time.

#### 2.4.4 HPLC-MS settings for reduced mAb analysis

The column was equilibrated at a flow rate of 500 μL/min with 24% B for 1.0 min. Gradient elution was performed at halved flow rate (250 μL/min) with a linear increase from 24% to 30% B within 7.5 min. This was followed by a washing step at 80% B for 1.5 min. Re-equilibration was done at 24% B for 4.0 min and at doubled flow rate (500 μL/min) for one more minute. MS acquisition settings are described in Table 1.

#### 2.4.5 HPLC-MS settings for analysis of IdeS digested mAbs

The column equilibration was done at a flow rate of 500 μL/min with 20% B for 1.0 min. Then, the flow rate was reduced to 250 μL/min and eluent B was linearly increased to 40% within 7.5 min. At 80% B, the column was washed for 1.5 min and re-equilibrated for the next injection at 20% B for 5.0 min. MS acquisition settings are described in Table 1.

#### 2.4.6 nMS of intact mAbs

For nMS analyses, mAbs were diluted to 0.10 μg/μL in 175 mmol/L AmAc. The samples were ionized with static nanoelectrospray ionization (nanoESI) using the following settings: spray voltage, 1.4 kV, in-source fragmentation, 60−200 eV. Mass analysis was conducted on a Q-Exactive Plus instrument at a resolution setting of 35,000 at *m/z* 200 with rolling averaging for approximately 2 min.

### 2.5 Meta-data analysis

Relevant publications considered for the meta-data analysis were searched by Google Scholar including mAb-name (i.e., adalimumab), drug market name (i.e., Humira), structural level (i.e., heavy chain, Fc, subunit, glycopeptide, intact), and glycosylation. A total of 18 publications were considered (Table S1). A glycan table was established in Microsoft Excel and corresponding Venn diagrams were generated using the R-package ggVennDiagram (v1.2.2) [25].

### 2.6 Data evaluation

Glycopeptide data were evaluated using Byonic (for settings refer to Table S2, v3.11.3, Protein Metrics Inc.). Glycopeptide quantification was achieved by Skyline (v20.2). Quantification of glycans at the subunit and intact levels was conducted by the in-house written, freely available R-package fragquaxi (v1.0), as already described elsewhere [19,20]. Prior to fragquaxi quantification, MS .raw files were converted to .mzml files using ThermoRawFileParser (v1.7.1) [26] with the following settings: mzML spectrum, no peak picking, all MS levels, and ignore missing instrument properties. Glycan annotation at the intact protein level was achieved by the modification finder (MoFi) software [13]. Zero-charge spectra of intact proteins were obtained by BioPharma Finder™ (v3.0, Thermo Scientific) using the following deconvolution parameters in the “Default Native” method: as source spectra method, the sliding windows and for deconvolution, the ReSpect™ algorithm were used. Fractional abundance plots were generated in RStudio using the ggplot2 package [27] as well as the ggbreak (v0.1.1) package [28]. Venn diagrams were created in RStudio using the ggVennDiagram (v1.2.2) package [25]. The heatmap plot was created in RStudio using the ComplexHeatmap (v2.13.1) package [29,30]. Hexosylation bias was eliminated according to a workflow published by Esser-Skala et al. [22] utilizing the CAFOG algorithm executed in Python (v3.9). A summary for data generation and evaluation workflow is provided in Fig. S2. Further, a comprehensive step-by-step workflow for data evaluation is provided (chapter 4 of the Supplementary data including Figs. S3–S29 and Table S3 and S10). All raw data, excel files, and R-scripts used for data evaluation are listed in the Supplementary data and are to be downloaded from Zenodo (https://doi.org/10.5281/zenodo.10455819).

## 3 Results

### 3.1 Capillary electrophoretic profiling of released glycans

Capillary electrophoresis, which is capable of separating charged species based on differences in charge and/or hydrodynamic radius, has been shown to facilitate the profiling of a broad range of glycans enzymatically or chemically released from glycoproteins [16,31,32]. Covalent and stoichiometric labelling of released glycans with a fluorescent dye (8-aminopyrene-1,3,6-trisulfonic acid, APTS) combined with detection by laser-induced fluorescence (LIF) holds the advantage of an equal molar response factor for all derivatized glycans such that the relative abundance of glycans can be directly derived from fluorescence signals.

**Fig. 1:**
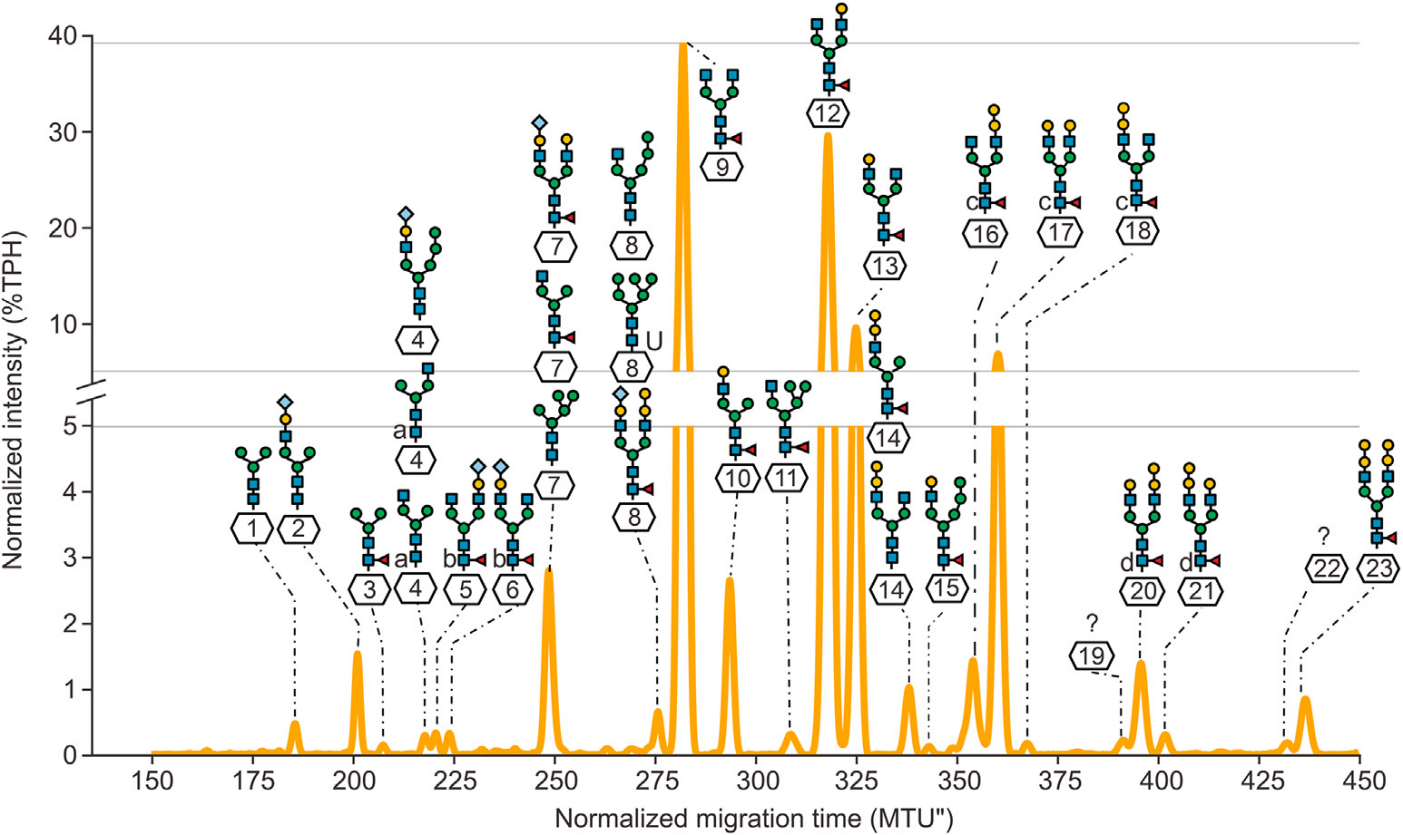
Glycofingerprint (aligned and normalized electropherogram) of NISTmAb released *N-*glycans analysed by multiplexed capillary gel electrophoresis with laser-induced fluorescence (xCGE-LIF). This electropherogram reveals the analysis of released *N-*glycans, where a total of 38 different glycan structures have been assigned to 21 out of the 23 peaks detected. Notably, glycans of the same mass are identified with superscript letters positioned to the left of the glycan, as exemplified by the alpha-Gal containing species FA2G2aG1[6] (labelled as d, peak 20), which shares the same mass as FA2G2aG1[3] (labelled as d, peak 21). In the glycan nomenclature for reversed phase-high-performance liquid chromatography-mass spectrometry (RP-HPLC-MS)-detected glycans, both are classified as FA2G3. For detailed glycan nomenclature information, please refer to Fig. S2. MTU'': migration time units; TPH: total peak height.

In order to characterize released NISTmAb glycans, xCGE-LIF analysis was carried out. Fig. 1 illustrates the glycofingerprint (migration time aligned and signal normalized electropherogram) of glycans released from 200 ng NISTmAb, wherein approximately 2 ng were injected per run. Based on migration time-matching with an internal xCGE-LIF database that contained over 300 *N-*glycan structures, one to three distinct *N-*glycan structures were assigned to 21 of the 23 detected peaks (Table S4). Using this straightforward migration time-matching as a screening method, good results can be obtained quickly and easily, but as can be seen from the peak annotations, a significant number of labelled glycans showed co-migration, e.g., Man5, FA1G0[3] and FA2G2Sg1(2,6)[3] in peak seven, which prevented the unambiguous annotation of corresponding glycan structures (for details of glycan nomenclature, refer to Fig. S13). On the other hand, some glycans with identical monosaccharide compositions but different linkages, representing stereoisomers such as FA2G1Sg1(2,6)[6] and FA2G1Sg1(2,6)[3], were clearly separated by CGE (peaks 5 and 6), while they are isobaric and thus indistinguishable by MS. For the later annotation of glycopeptides and glycosylated proteins and protein subdomains, we considered all 28 possible glycan structures. Diastereoisomers were counted as one glycan structure, because they are indistinguishable by MS resulting in 21 unique glycan moieties (Table S4).

**Fig. 2:**
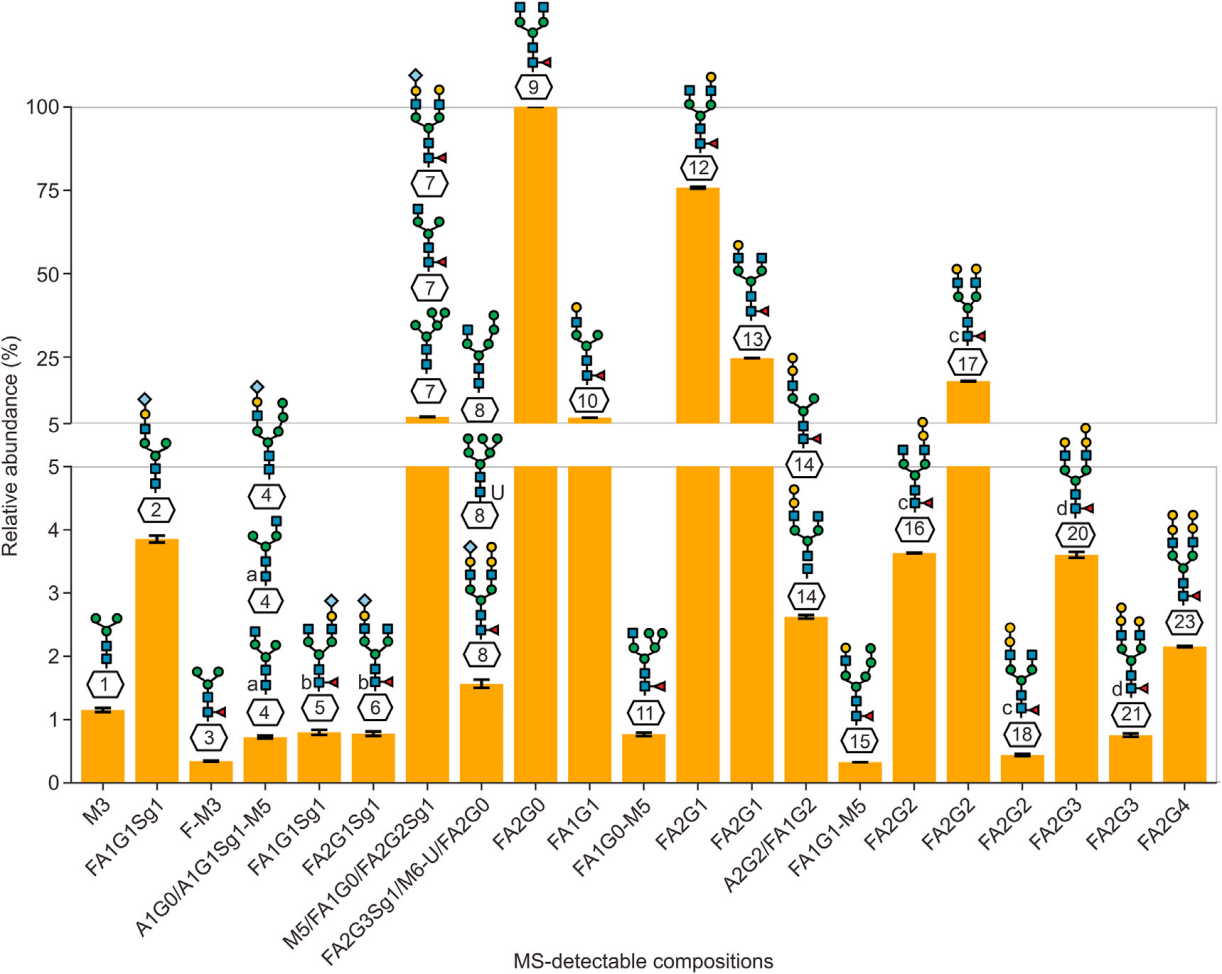
Relative abundances of NISTmAb released *N-*glycans analysed by multiplexed capillary gel electrophoresis with laser-induced fluorescence (xCGE-LIF). The predominant glycan species is of complex type with core fucosylation (FA2G0). Please note that no glycans were annotated to peaks 19 and 22 in Fig. 1. Therefore, the corresponding bar of relative abundance is missing in this figure. For detailed glycan nomenclature information, please refer to Fig. S2; corresponding raw data is provided in Table S4.

Next, annotated peaks in the glycofingerprint were relatively quantified. Relative abundances, normalized to the intensity of the most abundant glycan peak at 280 migration time units (MTU'') (aligned migration time units), of the different glycans are depicted in Fig. 2. The most intense of the 23 peaks detected in the glycofingerprint is associated with only one glycan, namely FA2G0. The second and third most abundant peaks correspond to the glycan FA2G1. Fifteen of the signals showed a relative abundance of below 5%, which we define here as threshold for low-abundant glycans or glycoforms. Combined with quantitative information from released glycan analysis and published literature on NISTmAb (reference material RM 8671) [16,33,34], the three major glycosylation variants are biantennary and core-fucosylated (F) with zero, one, or two galactose units (G) attached: FA2G0, FA2G1, FA2G2. As far as low-abundant glycans are concerned, FMan5 [D3]-A1G1 was the lowest abundant glycan (0.33%), which was detectable only in one of five technical xCGE-LIF replicates. The smallest peak to which several glycans were annotated in all five xCGE-LIF replicates had a relative abundance of 0.73% (peak 4, Fig. 2). FA2G1Sg1 (2,6)[3] was the lowest abundant uniquely annotated glycan which was detected in all five replicate analyses (peak 6, 0.78%). Relative standard deviations (RSDs) of relative peak heights in five technical replicates ranged between 0.14% and 5.14% and indicated excellent precision of relative glycan quantification by xCGE-LIF (Table S4). In summary, the identified glycans were at least present in one of five measurements, and glycans detected in more than three replicates had remarkably low standard deviations even for low abundant species. Thus, this method can be considered as robust and reproducible.

### 3.2 Site-specific glycan annotation and quantification at different molecular and structural levels of glycosylated polypeptides

**Fig. 3:**
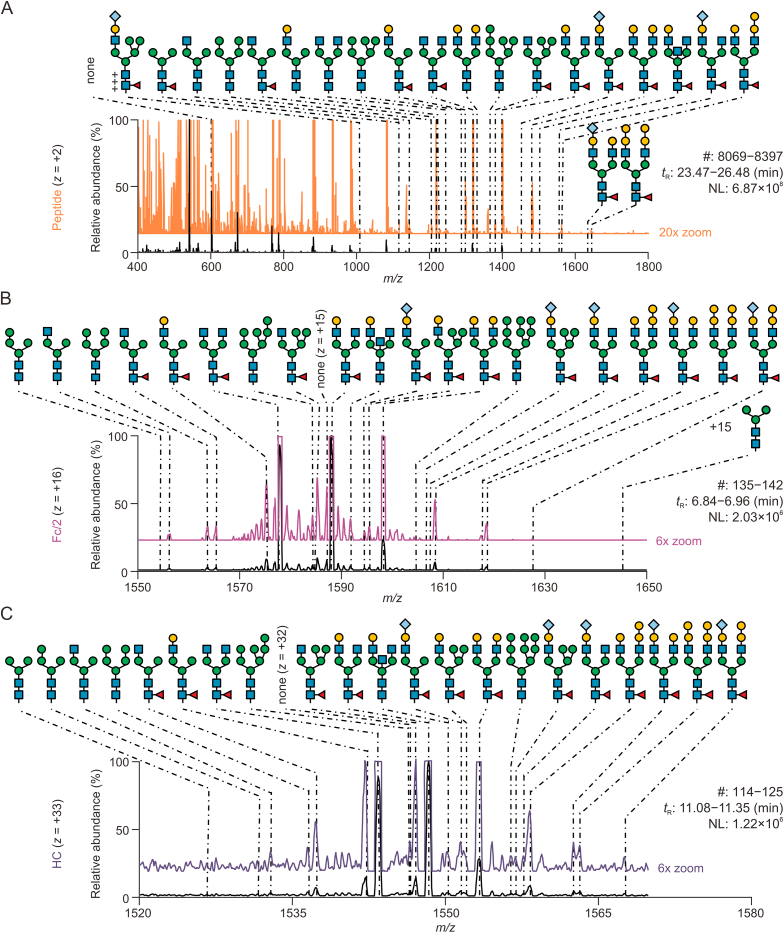
Glycovariant annotation at three structural levels of NISTmAb, i.e., peptide, Fc/2, and heavy chain (HC). Separation of polypeptides was accomplished by means of reversed phase-high-performance liquid chromatography-electrospray ionization-mass spectrometry (RP-HPLC-ESI-MS). For experimental details, see methods section. (A) EEQYNSTYR-glycopeptide mass spectrum within the 23.47–26.48 min retention time (*t*_R_) window. (B) Glycosylated Fc/2 at charge state +16. (C) Glycosylated HC at charge state +33. Within each spectrum, glycans are annotated. Magnifications provide insight into minor abundant species. #: number of MS scans; NL: intensity at 100%. For detailed glycan nomenclature information, please refer to Fig. S2.

The CH_2_ domain in the Fc region of the IgG heavy chain imbeds the highly conserved *N-*glycosylation site at Asn297. Employing different approaches of chemical or enzymatic dissection of the intact antibody, we analysed polypeptides of different sizes that incorporate glycosylated Asn297 for relative quantification of associated glycovariants. Fig. 3 provides an overview of the different glycovariants observable in mass spectra recorded during the chromatographic separation of NISTmAb glycopeptides generated upon tryptic digestion (tryptic peptide of nine amino acids, monoisotopic mass 1188.505 Da, Fig. 3A), of glycosylated Fc/2 region obtained through proteolytic cleavage by IdeS (Fc/2 region of 210 amino acids, average mass 23,783 Da, Fig. 3B), and of glycosylated heavy chain after reductive cleavage of intermolecular disulfide bridges (heavy chain of 449 amino acids, average mass 49,454 Da, Fig. 3C).

**Fig. 4:**
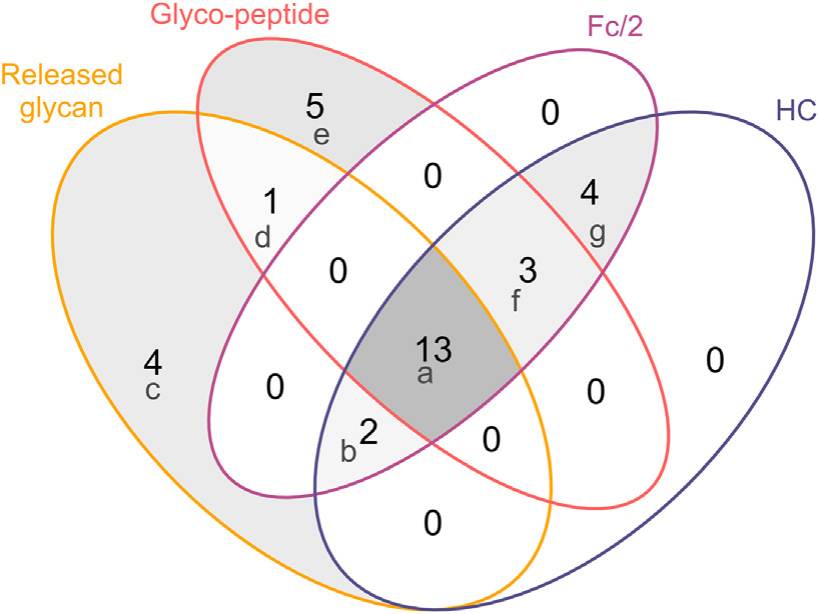
Comparing glycan detection across protein structural levels of NISTmAb. The Venn diagram illustrates the number of detected glycans at the released glycan, peptide, Fc/2, and heavy chain (HC) level. The following glycans are shared or unique to specific groups: a: A1G0, FA1G0, FA1G0-M5, FA1G1, FA1G1Sg1, FA2G0, FA2G1, FA2G1Sg1, FA2G2, FA2G2Sg1, FA2G3, FA2G4, M5; b: FA1G1-M5, M3; c: A1G0-M5, A2G2, FA1G2, M6-U; d: M3-F; e: A1G1, A2G0, A2G1, FA3G1, M6; f: A1G1SG1-M5, M7, none (unoccupied glycosylation site); g: A3G1, FA2G3Sg1, M4, M9. It's important to note that isobaric glycans, which were distinguished by released *N-*glycan analysis, were counted as one species for the comparison in the Venn diagram. For example, even though in the released glycan analysis, the two glycans FA2G1[6] and FA2G1[3] were recognized as two distinct species. They were counted as one, namely FA2G1, as they were also detected as one species by intact glycopeptide analysis. For detailed glycan nomenclature information, please refer to Fig. S2.

To explore the glycosylation of polypeptides, we have annotated mass spectra combining the intact mass of the polypeptide with the corresponding glycan structures identified at the released glycan level (Fig. 1). This analysis spans multiple structural levels, and, in this manner, 22 different glycopolypeptides were detected, each at the tryptic peptide- (Fig. 3A, and Tables S5 and S6), Fc/2- (Fig. 3B, and Table S7), and heavy chain level (Fig. 3C, and Table S7). The Venn diagram in Fig. 4 displays the overlap in detected glycopolypeptides, also incorporating released glycan identifications. Thirteen out of thirty-two glycan structures were detected at all different structural levels, whereas only four glycans were unique to released glycans and five to tryptic glycopeptide analysis. Between Fc/2 and heavy chain polypeptides, a 100% overlap was observed, of which four were unique to Fc/2 and heavy chain (A3G1, FA2G3Sg1, M4, M9), three were shared with the peptide level, and two were shared with the released glycan analysis (FA1G1-M5, M3). While non-glycosylated Asn297 is not amenable to released glycan analysis, it could be readily revealed at the glycopeptide, Fc/2, and heavy chain levels (Fig. 3, “none”). Only four released glycan structures remained unidentified at all polypeptide levels (A1G0-M5, A2G2, FA1G2, M6-U). This qualitative assessment is indicative of variations in the numbers of annotated glycans across different structural levels, including released glycans, glycopeptide, heavy chain, and Fc/2 (Figs. 3 and 4).

**Fig. 5:**
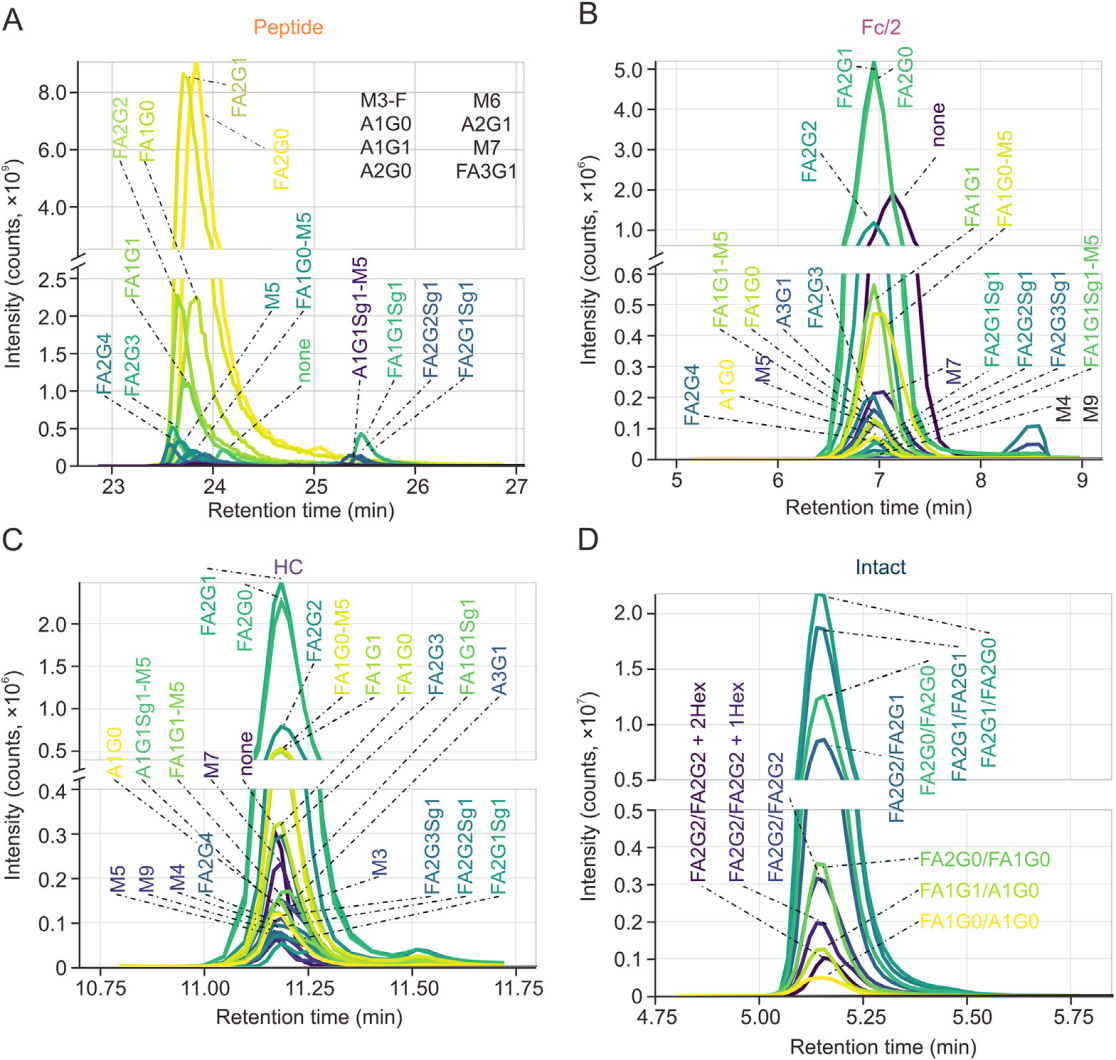
Extracted ion current chromatograms (EICCs) of the reversed phase-high-performance liquid chromatography-mass spectrometry (RP-HPLC-MS) measurements of glycopeptide, glycopolypeptides and intact NISTmAb. (A) EICCs for peptides obtained after tryptic digestion. (B) EICCs for Fc/2 obtained after IdeS treatment. (C) EICCs for heavy chain (HC) generated through intermolecular disulfide bond reduction. (D) EICCs for intact glycoprotein measured using denaturing RP-HPLC-MS. EICCs for glycopeptides were generated using Skyline, while those for Fc/2, HC, and the intact protein were manually generated with Xcalibur and Chromeleon from a single charge state. Full and original chromatograms can be found in Fig. S14 (peptide), Fig. S15 (subunits including Fc/2 and HC), and Fig. S16 (intact glycoprotein) for reference. For detailed glycan nomenclature information, please refer to Fig. S2.

To quantify specific glycosylated polypeptides, we generated extracted ion current chromatograms (EICCs) by utilizing the *m/z* ratios associated with the respective glycopolypeptides. While Skyline was used to generate EICCs for glycopeptides, EICCs for the remaining structural levels were created based on the most intense charge state in Chromeleon. Fig. 5 shows the resulting EICCs of NISTmAb glycopeptides generated upon tryptic digestion (Figs. 5A and S14), of glycosylated Fc/2 region obtained through proteolytic cleavage by IdeS (Figs. 5B and S15A), and of glycosylated heavy chain after reductive cleavage of intermolecular disulfide bridges (Figs. 5C and S15B), as well as at the intact protein level (Figs. 5D and S16).

Glycosylated tryptic peptides eluted in a time window between 23.5 and 25.5 min with sialylated glycopeptides shifted to the upper edge of the retention time window, which indicates a significant influence of the glycan on chromatographic retention. Glycovariants of the Fc/2 regions showed only a slight variation of retention times with sialylated variants eluting somewhat later, while retention differences at the heavy chain level were minimal. Separation of Fc/2 species appears to be mainly impacted by the glycan moiety, with the non-glycosylated variant eluting after the glycosylated species (Fig. S17). Taking advantage of specific glycan masses based on monosaccharide composition, the R-based fragquaxi script [19] was utilized to extract and calculate peak areas from the different EICCs to facilitate the relative quantification of the glycopolypeptide variants.

**Fig. 6:**
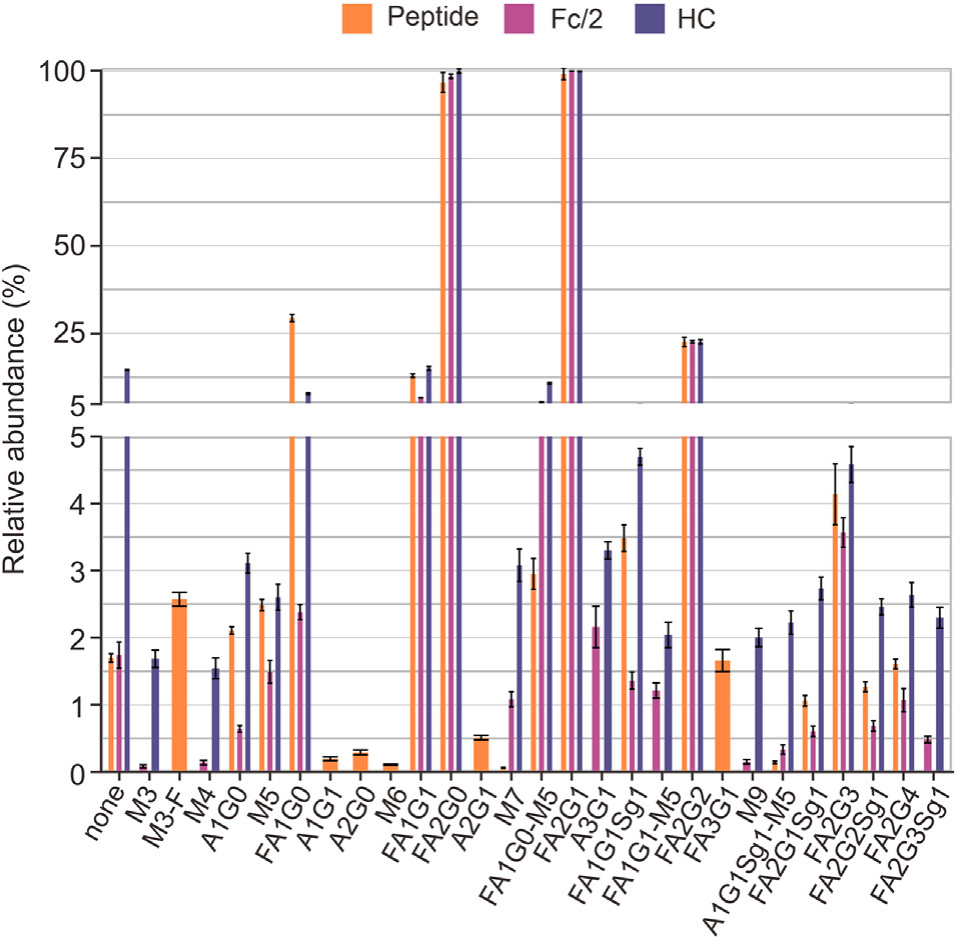
Quantitative glycosylation profiling of NISTmAb (poly)peptides across three structural levels: glycopeptide, Fc/2, and heavy chain (HC). Relative abundances of glycans are graphically represented for quantitative assessment of glycosylation patterns at different structural levels. The thickness of the bars signifies whether a particular glycan was detected at one, two, or all structural levels. To ensure accuracy, glycation bias in Fc/2 and HC levels has been corrected, using the correct abundances for glycation bias (CAFOG) algorithm [22] (see Fig. S19). For detailed glycan nomenclature information, please refer to Fig. S2.

Instead of relative quantification from deconvoluted mass spectra, which has been shown to be significantly dependent on deconvolution parameters [17], fragquaxi extracts peak areas directly from non-deconvoluted raw data [19]. For accurate quantification of glycovariants, we determined the degree of glycation (Fig. S18). Subsequently, relative abundances were corrected for the hexosylation bias according to Esser-Skala et al. [22] (Fig. S19). The relative abundances of all detectable glycovariants at the peptide-, Fc/2 region-, and heavy chain level can be deduced from the bar chart representing the relative intensities of quintuplicate analyses of the respective glycovariants (Fig. 6). The prevailing variants included the glycans FA1G0, FA1G1, FA2G0, FA2G1, and FA2G2, corroborating the results from released glycan analysis. While relative intensities of the high-abundant glycovariants showed very good congruence, significant differences were observed in the relative abundances of variants of below 5% relative abundance. Further, glycan structures, which were not detected at all levels were of a relative abundance of below 3% (Fig. 6).

To accurately depict the variations in the relative abundance of glycan-moieties at the different structural glycopolypeptide levels, we visualized the data using a heatmap (Fig. S20). Although the most abundant glycan at all levels was the same (FA2G1), the relative abundance of glycans was very variable. First, looking at the differences across glycopolypeptide levels, the relative abundance of some glycan-moieties was very different (FA1G0, M5, FA1G1, A1G0, FA2G2Sg1). On the other hand, the relative abundance of many glycan-moieties was similar at peptide and Fc/2 levels (FA2G2, M7, FA1G0-M5, FA2G1Sg1, FA2G4, none, FA2G3, A1G1Sg1-M5). Finally, for some glycans, there were also large differences within the quintuplicates (FA2G0, peptide and Fc/2 levels). Overall, this data shows considerable differences in relative abundance of all glycans at different molecular levels of analysis including glycopeptide, heavy chain, and Fc/2.

### 3.3 Assessing glycosylation variants of NISTmAb at the intact protein level

At the level of intact protein, both heavy chains of the heterotetrameric mAb may be glycosylated allowing the direct observation of paired glycoforms. We employed direct-infusion nMS for intact NISTmAb glycoform analysis due to its higher spatial resolution and minimal sample preparation requirements, accomplished through solvent exchange to 150 mmol/L ammonium acetate [3,23]. However, like subunit quantitative glycan profiling, the assignment of glycans is solely reliant on the total observed mass. It is important to recognize that a specific mass value can correspond to multiple *N-*glycan compositions. For example, certain hexoses like mannose, galactose, and others share a common mass of 162 Da, and the combination of two fucose residues (146 Da each) resembles the mass of a single sialic acid (291 Da, Neu5Ac). This is also visualized in Fig. 5D, where reversed phase (RP)-HPLC-MS data were used to construct EICCs of glycovariant combinations. However, it is impossible to directly identify all possible combinations contributing to one peak. To address this complexity, we employed the MoFi [13] software tool, which plays a pivotal role in glycosylation pair assignments based on quantitative data obtained from the glycopeptide level.

**Fig. 7:**
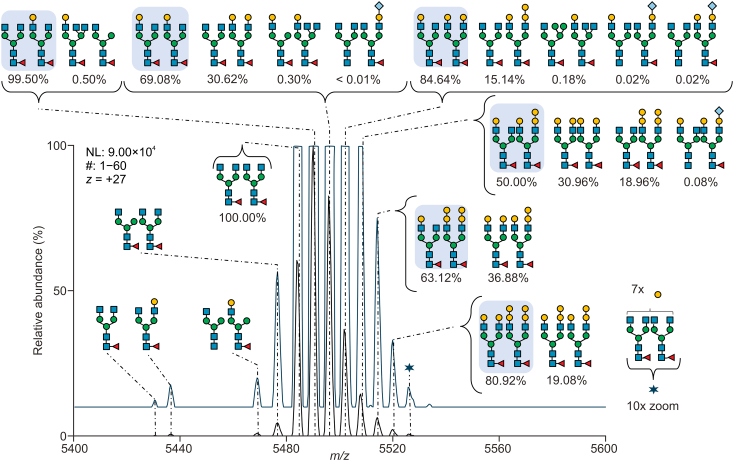
Analysis of glycoforms using direct-infusion native mass spectrometry (nMS). Depiction of the most intense charge state of native NISTmAb (first technical replicate) with annotated glycan compositions. A full spectrum of intact NISTmAb is displayed in Fig. S20. For detailed glycan nomenclature information, please refer to Fig. S2. #: number of MS scans; NL: intensity at 100%.

**Fig. 8:**
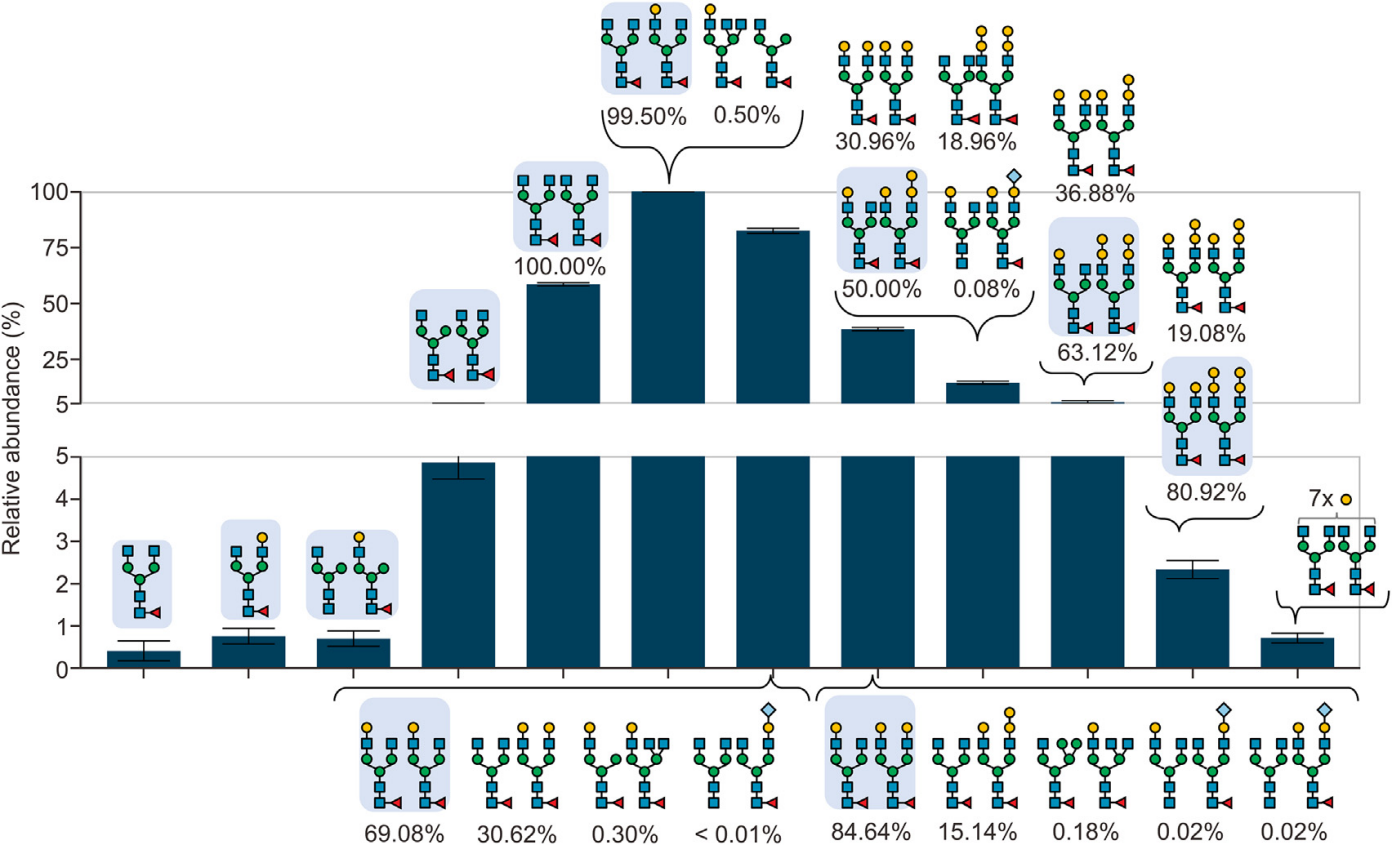
Relative quantification of glycosylation species of intact NISTmAb. First, spectra were deconvoluted to zero-charge using a deconvolution software (BioPharma Finder™). Second, permutations were calculated using MoFi (The corresponding permutation score is given below each glycan pair). Third, quantification was conducted on the raw data level using the R-package fragquaxi. Peaks that were not deconvoluted in the first step were annotated manually. Therefore, no permutation score is given for those abundances. For detailed glycan nomenclature information, please refer to Fig. S2.

Accordingly, the software provided a list of potential glycans for each observed mass, along with their respective contribution to the intensity expressed as a percentage (permutation score). This approach ensured a more accurate and comprehensive representation of glycan composition and distribution, as illustrated in Fig. 7, which depicts the 27-fold protonated form of NISTmAb. Signals for the different glycoforms spread over an *m/z* window of 5,420 to 5,540 without overlap with neighboring charge states (Fig. S21). Consequently, quantification at the intact protein level was performed on nMS measurements with its advantages having been demonstrated previously [3,35]. Since no chromatographic elution profile was inherent to this analysis, 60 scans were averaged before integrating the peak areas in the averaged mass spectrum to obtain the relative abundances of the glycoforms represented in the mass spectrum. The distribution of glycoforms featuring paired glycans is illustrated in Fig. 7. We consider this distribution of intact glycoforms the most realistic representation of the relative abundance of NISTmAb glycoforms, since the influence of glycan structure on ionization efficiency should be minimal in the large glycoprotein. However, a realistic picture of the underlying glycosylation species can be obtained only with information from other structural levels. Data evaluation using the MoFi software overcomes the ignorance of isobaric glycoforms at the intact protein level. It is important to emphasize that in the MoFi workflow, deconvoluted spectra are utilized as input. Consequently, peaks that elude the deconvolution process are limited to only one, manually annotated variant (Fig. 8).

### 3.4 Application and assessment of multi-level quantification workflow

**Fig. 9:**
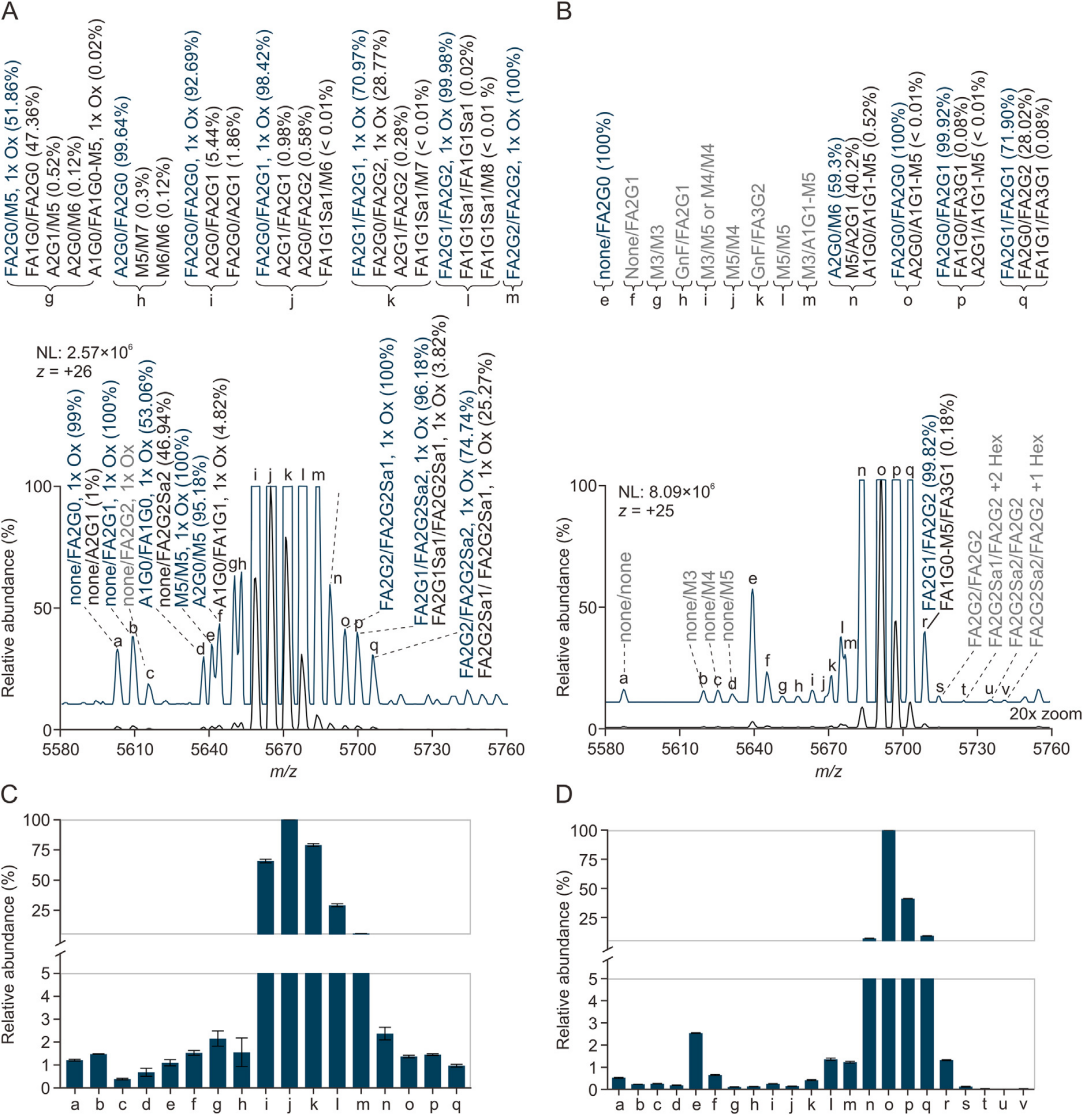
Annotation of glycosylation variants to the native mass spectrum of (A) intact rituximab and (B) adalimumab, and (C, D) their relative quantification. The contribution of each glyco-combination is given in brackets as permutation score provided after automated annotation using theMoFi software. Besides glycosylation, oxidized (1x Ox) variants were also quantified in rituximab (A, C). Variants with major contribution are highlighted in blue. Manually annotated variants are marked as grey. For detailed glycan nomenclature information, please refer to Fig. S2.

To further assess the workflow employed in our study, we conducted a thorough evaluation of the glycosylation profiles of two additional IgGs, i.e., therapeutic antibodies rituximab (MabThera) and adalimumab (Humira). Following the same methodology as applied to NISTmAb, we collected released *N-*glycan data using xCGE-LIF (as depicted in Figs. S22–S24, and Tables S8 and S9), determined the degree of glycation for deglycosylated subunits as well as for deglycosylated intact mAbs (Fig. S25), generated peptide and subunit-level data using RP-HPLC-MS (Fig. S26 for rituximab and Fig. S27 for adalimumab) and performed intact-level analysis using direct-infusion nMS (illustrated in Fig. 9). Annotation of glycan combinations at the intact protein level as aided by MoFi is shown in Figs. 9A and B. This comprehensive approach enabled assessment of glycosylation profiles across all structural levels, facilitating the integration of data from the lower to higher structural levels.

Generally, the glycosylation profile of rituximab is characterized by the presence of *N-*acetylneuraminic acid species, while adalimumab harbors a variety of oligomannose species (Fig. 9). Even though clearly present in the raw spectrum of adalimumab (Fig. 9B, grey), many peaks escaped MoFi annotation as they were not considered by the deconvolution software. Across all structural levels, all three mAbs share similar major glycosylation species, which are of the complex type with core-fucosylation and varying degrees of galactosylation. Comparison of glycan profiles corroborates findings already observed for NISTmAb, with notable discrepancies at different structural levels (glycopeptide, Fc/2, and heavy chain), particularly in the sub-5% relative abundance range. Some overarching trends that emerged from our analysis include: (i) Non-glycosylated species are relatively more abundant at the heavy chain level for all three mAbs compared to the Fc/2 or peptide level. (ii) For most glycovariants, heavy chain relative abundances surpass those of glycopeptides and Fc/2, although there are exceptions, such as FA1G0 in all three mAbs and FA2G2 in rituximab and adalimumab–with both points (i and ii) being reflected in Figs. 6, S26, and S27. (iii) RSDs tend to be greater at the Fc/2 level for NISTmAb and at the peptide level for adalimumab. Notably, RSD values remained below 30% for most glycovariants in NISTmAb, whereas for adalimumab, some reached as high as 60% (as illustrated in Figs. S28 and S29). As can be observed from Table S8, only two NISTmAb glycans (FA2G1 and FA2G2) did not exhibit significantly different relative abundances at the subunit levels (Fc/2 and heavy chain), while for most glycovariants (except FA1G1 and FA2G1), no significant difference in standard deviations was observed.

### 3.5 Unveiling the glycosylation diversity of mAbs in literature: a comprehensive meta-data analysis

In this meta-data analysis, we screened findings of various studies (Table S1) focusing on the glycosylation profiling of mAbs, including NISTmAb, rituximab, and adalimumab at the peptide, polypeptide, and intact levels. This analysis aims at providing a comprehensive overview of the qualitative and quantitative aspects of *N-*glycan structures associated with these antibodies.

**Fig. 10:**
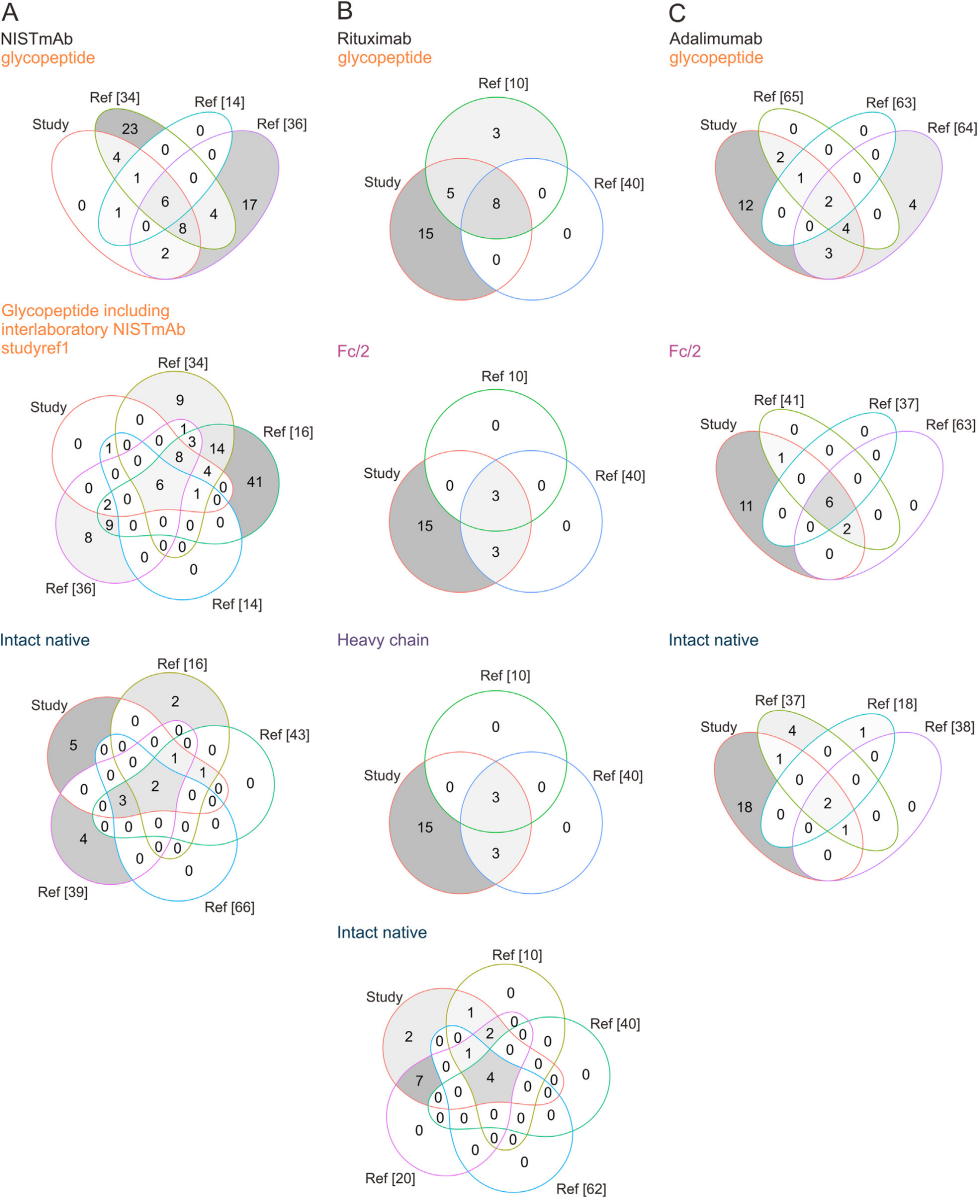
Meta-data analysis on *N-*glycosylation of mAbs in literature at diverse structural levels for (A) NISTmAb, (B) rituximab, and (C) adalimumab [[10], [14], [16], [18], [20], [34], [36], [37], [38], [39], [40], [41], [43], [62], [63], [64], [65], [66]]. More details about the references are summarized in Table S1. Data presented in this manuscript are labelled with “study”. For NISTmAb, only those studies working with the RM 8671 were considered in the meta-analysis. A full list of glycans is provided in the online Zenodo supplement (https://doi.org/10.5281/zenodo.10455819, Literature_comparison.xlsx).

When considering glycopeptide profiling of NISTmAb, it becomes evident that two specific studies [34,36] have identified a significantly larger number of glycopeptides than our analysis, as depicted in Fig. 10A. This discrepancy points to limitations in our glycopeptide mapping protocol, as our study detected only 22 glycopeptides within five replicate measurements. Zhao et al. [34] and Bi et al. [36] utilized highly advanced glycoproteomic workflows, while in our study glycopeptide measurements covered one of five structural levels. However, most of those additional glycans had relative abundances below 0.1% [34]. Notably, in comparison to those studies, structures containing *N-*acetylneuraminic acid and α-Gal species were mostly absent in our approach. Augmenting our analysis with data from the inter-laboratory NISTmAb study [16], which incorporated data from all structural levels, revealed a total of 88 glycan structures attributed to NISTmAb. Impressively, half of these structures were unique to this particular study. While some previous studies [18,37,38] presented data on intact NISTmAb, they focused on the major glycan species, neglecting the minor, low-abundant ones. This highlights the benefit of our multi-level quantification approach, where twelve peaks could be quantified in the intact NISTmAb spectrum and a total of 25 glycan combinations were annotated (Fig. 7). In a previous study [39], application of a strong cation exchange (SCX)-chromatographic separation prior to MS analysis enabled distinct detection of *N-*glycolylneuraminic acid containing species at the intact protein level (Fig. 10A), which in our approach were detected based on the MoFi algorithm.

In the case of rituximab, the literature offers coverage across all structural levels considered in this meta-analysis study, as demonstrated in Fig. 10B (peptide, polypeptide and intact level). For instance, a study by Montacir et al. [40] analysed rituximab at various levels: intact, subunit (Fc/2 and heavy chain), and peptide levels, quantifying four, six and eight glycan variants, respectively. Notably, Carillo et al. [10] did not detect sialylated species at the rituximab subunit level, possibly due to overly strict deconvolution parameters. To our knowledge, no study has detected a greater number of glycans at the subunit level than our multi-level approach.

The glycosylation profiling of adalimumab has also garnered attention in the literature (Fig. 10C). Zhu et al. [37] investigated adalimumab at the intact level and utilized a middle-down approach, quantifying predominantly complex-type glycovariants with sialylation but notably lacking oligomannose species. In contrast, our multi-level integrative approach identified a total of 30 different glycosylation variants from 22 peaks of the intact native adalimumab spectrum. Duivelshof et al. [41] analysed adalimumab's glycosylation profile at the middle-up level, identifying ten glycan structures through a released-glycan analysis. However, they did not quantify hybrid-type glycans or unglycosylated species. Another study by Millán-Martín et al. [18] in 2021 performed a charge-variant analysis of adalimumab using intact MS but identified only three glycovariants, however the primary focus was on optimizing deconvolution parameters.

## 4 Discussion

In the present study, we provide a workflow to quantify minor abundant glycosylation species of biotherapeutic proteins. Our approach utilized released glycan data obtained by xCGE-LIF analysis to integrate qualitative and quantitative results in further analyses involving glycopeptide quantification, subunit glycoprofiling and intact glycoprotein assessment with an emphasis on data evaluation mostly at the un-preprocessed raw data level. We elaborated our approach on NISTmAb, a mAb reference material, which has been frequently used as a reference compound to demonstrate applicability of various analytical workflows [16,33,42,43].

Our multi-level quantification approach relied on identified released glycans obtained by xCGE-LIF analysis which, in contrast to MS, does not suffer from differing signal response factors due to analyte structures [44,45]. Upon xCGE-LIF analysis, 38 *N-*glycan structures were identified; abundances of major glycans correlated with previous studies on the same reference material (RM 8761) [33,34,42]. In addition, we acquired relative abundance data by means of HPLC-MS/MS analysis of tryptic peptides of NISTmAb. Notably, the effect of different ESI efficiencies is greatly reduced if not eliminated already at the glycopeptide level for neutral glycans, as demonstrated by Stavenhagen et al. [45], however attention has to be paid to sialylated species [45,46]. In addition, glycosylated peptides exhibited a 10%−50% lower signal intensity compared to non-glycosylated species [45], which may slightly bias the relative quantification based on HPLC-MS analysis. From a chromatographic point of view, depending on the attached sugar moiety, glycopeptides showed different retention behavior (Fig. 5A). *N-*glycolyneuraminic acid containing glycopeptides eluted later due to their stronger interaction with the C_18_ matrix because of their charge-altering properties [47,48].

Glycopeptides were quantified using the software Skyline [21], wherefore potential glycans derived from the released glycan analysis (xCGE-LIF) were considered for relative quantification (Table S3). At the subunit level, heavy chain (obtained after disulfide bond reduction) and Fc/2 (generated after enzymatic treatment with IdeS) species-related glycans were quantified at the raw data level using an in-house written R-package *fragquaxi*. This algorithm calculates fractional abundances of extracted ion currents within a specified number of scans for the provided glycovariants, as can be observed in Fig. S21. The advantage of *fragquaxi*-based quantification compared to deconvolution-based quantification has been elaborated in a previous study [19]. Considering one-sided glycan assessment, 28 glycans were quantified at the peptide, and subunit levels of which 16 glycans were shared by all three approaches (Fig. 4, NISTmAb analysed at peptide, Fc/2, and heavy chain level). Notably, at the peptide and subunit levels, no information about actual glycan connectivity on the IgG molecule is obtained.

To obtain the most probable glycan combinations at the intact protein level, we supplied the software tool MoFi with quantitative glycopeptide data. MoFi has been utilized in previous studies for glycoform annotation in highly complex glycoproteins [3,13,23,49]. The observed glycosylation pattern of NISTmAb agrees with previous studies [34,43,50,51], with the singly galactosylated (FA2G0/FA2G1) as major variant, followed by the doubly (FA2G1/FA2G1) and non-galactosylated (FA2G0/FA2G0) species. The fractional abundances of the major glycoforms differed slightly in all those studies, which were based on deconvoluted spectra. On the contrary, we quantified glycoforms directly from raw mass spectra (likewise to subunit level quantification). However, intact protein glycoform quantification directly from raw data has the prerequisite of knowledge about the attached glycans prior to quantification.

The complexity of intact NISTmAb spectra could be attributed to different levels of galactosylation and core-fucosylation. Especially for minor abundant species, chemical or enzymatic dissection (i.e., subunit level analyses) yielded simplified mass spectra. Compared to peptide analysis, which is laborious in terms of sample preparation and data generation, subunit glycoprofiling is considerably faster (15 min sample preparation plus 15 min HPLC-MS analysis). Based on the obtained subunit level data, we were able to quantify minor abundant sialylated NISTmAb species (Fig. 6). As observed by Stavenhagen et al. [45] and Čaval et al. [46], the presence of sialic acid species has negative effects on ionization and thus quantification at the peptide level, which may lead to an underestimation of sialylated glycovariants. Especially, glycans containing *N-*glycolyneuraminic acid are critical due to their immunogenic properties in humans [52].

Reliable detection and accurate quantification of *N-*glycans and other PTMs is a critical aspect in the pharmaceutical industry [2]. Different sugar moieties have different pharmacological effects. For example, glycans decorated with terminal galactose exhibited faster serum clearance [53], mAbs with oligomannose glycans showed altered effector functions (ADCC, CDC) [54], and the non-enzymatic attachment of glucose has potential effects on the biological activity of mAbs depending on the site of glycation [55]. In the present study, the latter modification was addressed at the subunit and intact protein level by taking into account the issue of hexosylation bias arising from isobaricity of various hexoses (glucose, mannose, galactose) as well as multiple modification sites. For this purpose, we applied a recently published Phyton-based algorithm [22] resulting in altered glycosylation profiles upon correction for glycation (Fig. S19). Implementation of this computational step in subunit and intact glycoprotein analysis of therapeutic mAbs is thus recommended in order to obtain accurate glycoform profiles.

Concerning the quantification of glycans at the peptide and subunit levels, significant discrepancies were observed for minor abundant glycosylation variants in the present study (Fig. 6, <5% relative abundance). Direct comparison of relative glycan quantities obtained from different structural levels was previously described in a few studies [41,56,57]. Hines et al. [57] employed a “data-supported glycopeptide detection” with optimized ionization settings. Furthermore, sialylated glycoforms might decompose to smaller glycosylation species falsifying quantification [57]. Therefore, specific ionization and fragmentation properties of each glycoform to be quantified were considered and data were corrected by conversion factors, with the results showing high correlation of glycopeptide and released glycan quantitative information [57]. Furthermore, relative abundances of glycopeptide variants also depend on the charge state of the quantified peptide (i.e., +2 or +3) and whether only full or also miss-cleaved glycopeptides are considered [56]. Therefore, a targeted data acquisition with optimized ESI-MS parameters might provide most accurate glycopeptide quantities [56]. However, for neutral glycans, it was shown that ESI-based glycopeptide quantification correlated well with fluorescently labelled released glycan quantification [45,58]. In conclusion, reliable quantification relies on robust output signals and ionization efficiencies independent of the structure of the attached glycan.

A recently published study [59] by the US Food and Drug Administ-ration (FDA) in November 2023 underscores that the majority of IgG-based mAbs feature complex-type glycans dominated by zero, one, and two galactoses, constituting a significant portion of their glycovariants, while minor abundant species remain below 3%. Among these minor species, oligomannose, afucosylated, or sialic acid-containing glycans stand out as contributors to the top 10 glycan structures in mAbs [59]. Even though those variants occur to a low percentage of relative abundances, they have significant effects on efficacy of mAbs. For example, oligomannose species bearing five mannoses show faster serum clearance compared to other oligomannose variants [60]. Moreover, the minor abundant afucosylation has a drastic effect on ADCC activity: by increasing the afucosylation level in mAbs from 10% to 62%, a 1.89 and 5.93-fold increase in ADCC was observed by Thomann et al. [5]. These findings emphasize the need for accurate quantification of less-explored glycan variants. The remarkable heterogeneity of glycoproteins, particularly biotherapeutic proteins like mAbs, necessitates diverse analytical strategies for comprehensive characterization, evident from the numerous workflows and inter-laboratory studies found in the literature [[14], [15], [16],^61^].

The findings described here highlight the rich diversity of glycan structures associated with therapeutic mAbs and underscore the potential for further research and optimization in glycopeptide analysis. It is worth noting that there is a notable gap in the quantification and examination of minor, yet frequently occurring glycosylation species. The prevailing focus in the majority of studies has been on introducing novel methods, sample preparation techniques, and data evaluation, leaving room for increased attention to the detection and quantification of these lesser-explored glycan variants in future investigations.

In summary, our workflow's utility for biopharmaceuticals is demonstrated through its application to two additional therapeutic IgG molecules, adalimumab and rituximab, with the most precise determination of minor abundant glycosylation species achieved at the peptide and subunit levels. Furthermore, our meta-data analysis presents a comprehensive overview of the glycoprofiling of NISTmAb, rituximab, and adalimumab, highlighting the strengths and limitations of various studies within the field and reveals that most studies primarily investigate protein glycosylation through glycopeptide mapping (Fig. 10) [[10], [14], [16], [18], [20], [34], [36], [37], [38], [39], [40], [41], [43], [62], [63], [64], [65], [66]] or released glycan analysis. Notably, when considering analysis at the intact protein level, our raw data-based multi-level approach surpasses the performance of most other studies. However, it is essential to acknowledge that raw data-based intact glycoprotein quantification depends on prior knowledge of expected glycan profiles. The described intact protein quantification workflow becomes highly relevant when precise glycosylation profiling is required, such as in batch-to-batch comparisons or biosimilarity assessments, offering the advantage of eliminating the need for deconvolution algorithms [17,19].

## 5 Conclusions

In conclusion, our study examined reliable detection and quantification methods of glycans in biopharmaceutical drugs. Employing a multi-level quantification approach with three mAbs (NISTmAb, rituximab, and adalimumab), we focused on minor abundant glycovariants, revealing notable quantitative variations at different structural levels, especially for glycans occurring at less than 5% relative abundance with respect to the major variant. The use of open-source data-evaluation tools like Skyline and the R-package *fragquaxi*, coupled with data integration of different structural levels from released glycans up to intact glycoproteins, facilitated a comprehensive analysis. Additionally, we corrected glycosylation abundances for glycation, mitigating potential false results due to the inability to differentiate between hexoses contributing to galactosylation, oligomannose species or glycation. MoFi enabled the annotation of mass peaks, revealing isobaric glycosylation variants at the intact protein level, exposing otherwise hidden glycan combinations. Demonstrating the workflow's utility on NISTmAb, rituximab, and adalimumab, our study offers a novel perspective by profiling minor abundant variants across diverse structural levels. A meta-data analysis comparing literature on glycosylation of these antibodies revealed a predominant focus on major glycosylation variants, often limited to glycopeptide-level screening. Our study advances understanding and accessibility in glycosylation analysis, spotlighting minor abundant glycovariants in therapeutic antibodies.

## Declaration of generative AI and AI-assisted technologies in the writing process

During the preparation of this work, the author(s) used the Large Language Model ChatGPT (v3.5, https://chat.openai.com) in order to improve and condense text passages. After using this tool/service, the author(s) reviewed and edited the content as needed and take(s) full responsibility for the content of the publication.

## Data availability statement

All data, including MS-raw files (Glycopeptide_S-trap.rar, Adalimumab_1030241.rar, NISTmAb.rar, Rituximab_N7025B04.rar) and R-scripts (R-scripts_Supplement.rar), are accessible on Zenodo (https://doi.org/10.5281/zenodo.10455819). Supplementary figures and tables can be found in the online Supplement, accompanied by a detailed description for utilizing the data analysis of the multi-level data integration workflow. Readers are encouraged to refer to the provided documentation for guidelines and usage instructions.

## CRediT authorship contribution statement

**Katharina Böttinger:** Conceptualization, Data curation, Investigation, Methodology, Software, Writing – original draft. **Christof Regl:** Conceptualization, Data curation, Writing – review & editing. **Veronika Schäpertöns:** Data curation, Software, Visualization, Writing – review & editing. **Erdmann Rapp:** Data curation, Writing – review & editing. **Therese Wohlschlager:** Conceptualization, Writing – review & editing. **Christian G. Huber:** Conceptualization, Funding acquisition, Resources, Supervision, Writing – original draft.

## Declaration of competing interest

Dr. Erdmann Rapp is founder and CEO of the company glyXera GmbH (Magdeburg, Germany) that conducted released glycan analyses of which data were used in this study.
