# Supplementary material for: “Small is beautiful” – Examining reliable determination of low-abundant therapeutic antibody glycovariants
Source: J Pharm Anal. 2024 Apr 26;14(10):100982. doi: 10.1016/j.jpha.2024.100982 (PMC11755342; doi:10.1016/j.jpha.2024.100982)
Supplement: Multimedia component 1 [file mmc1.pdf]

Supplement to:
"Small is beautiful" – examining reliable determination of low-abundant therapeutic antibody glycovariants

Contents of the supplement

Contents of the supplement 1
List of supplementary figures 2
List of supplementary tables 3
1. Supplementary figures 4
2. Supplementary tables 29
3. Supplementary data 39
3.1. Superiority of the S-trap peptide generation protocol over classical C_{18} ZipTips 39
3.2. Detection of artifact modifications after de-glycosylation at subunit levels. 39
3.3. Assessment of PTMs other than glycosylation at the peptide level. 40
4. Instructions for multi-level data-integration workflow 40
4.1. Overview and organization of data and software tools 40
4.2. Installation of required software tools 42
4.3. Quantification of glycopeptides 42
4.4. Quantification of glycans at Fc/2 and HC 43
4.5. Correction of glycosylation abundances for glycation using the CAFOG algorithm 44
4.6. Comparison involving glycopeptides, Fc/2- and HC-glycans as well as qualitative comparison including released glycan data 44
4.7. Evaluation of glycosylation at the intact protein level 45
References 47

List of supplementary figures

Fig. S 1. Comparison S-trap vs. classical ISD with C_{18} ZipTip peptide purification on the example of rituximab pre-measurements. 4
Fig. S 2. Simplified workflow overview for data generation and evaluation. 5
Fig. S 3. Skyline – proteomics interface. 6
Fig. S 4. Skyline – edit transition settings.. 6
Fig. S 5. Skyline – edit peptide modifications. 7
Fig. S 6. Skyline – edit peptide. 7
Fig. S 7. Skyline – edit peptide list with different modifications. 8
Fig. S 8. Skyline – edit report. 9
Fig. S 9. Skyline – import results, i.e., the MS raw files. 9
Fig. S 10. Skyline – XIC of different charges.. 10
Fig. S 11. Skyline – observe results. 11
Fig. S 12. Graphical user interface (GUI) of MoFi [24]. The example is provided on rituximab. Input includes protein **Sequence**, PTM **Composition**, PTM **Structure**, and protein masses of the deconvoluted **Spectrum**. 12
Fig. S 13. Symbol nomenclature of *N*-glycans based on references [1,2]. 13
Fig. S 14. Extracted ion current chromatograms (EICCs) of the glycopeptide EEQYNSTYR of NISTmAb. 14
Fig. S 15. Total Ion Current Chromatograms (TICs) and Extracted Ion Current Chromatograms (EICCs) for IdeS-digested **(A)** and reduced **(B)** NISTmAb. 15
Fig. S 16. Total Ion Current Chromatogram (TICC, left) and Extracted Ion Current Chromatogram (EICC, right) of intact NISTmAb. 16
Fig. S 17. Separation of IdeS-generated Fc/2 and F(ab')2 species *via* RP-HPLC and the corresponding mass spectra (NISTmAb). 16
Fig. S 18. Raw spectra of PNGase F treated NISTmAb and quantification of glycation........ 17
Fig. S 19. CAFOG correction for glycation bias in NISTmAb. 18
Fig. S 20. Heatmap showing relative abundances of glycan-moieties at different structural glyco-(poly)peptide levels of NISTmAb... 19
Fig. S 21. Raw spectrum of intact NISTmAb acquired by native MS. 20
Fig. S 22. xCGE-LIF glycofingerprints (t_{mig} aligned and signal normalized electropherograms) of **(A)** rituximab and **(B)** adalimumab.. 21
Fig. S 23. Relative quantification of released glycans from rituximab, analysed by xCGE-LIF. 22
Fig. S 24. Relative quantification of released glycans from adalimumab, analysed by xCGE-LIF. 23
Fig. S 25. Analysis of glycation. 24
Fig. S 26. Quantitative **(A)** and qualitative **(B)** comparison of rituximab-glycan-moieties. ... 25
Fig. S 27. Quantitative **(A)** and qualitative **(B)** comparison of adalimumab-glycan-moieties. 26
Fig. S 28. Visualization of statistics for NISTmAb. 27
Fig. S 29. Visualization of statistics of adalimumab.. 28

List of supplementary tables

Table S 1. Publications considered for meta-data analysis. 29
Table S 2. Modification setting of Byonic peptide data evaluation. 30
Table S 3. Features in the Skyline output. 31
Table S 4. Released glycan data of NISTmAb. 32
Table S 5. Fractional abundances of the glycovariants of the EEQYNSTYR peptide of NISTmAb. 33
Table S 6. Number of occurrences of unknown modifications. 34
Table S 7. Statistics on NISTmAb Fc/2- and HC-glycans. 35
Table S 8. Released glycan data for rituximab as determined by xCGE-LIF. 36
Table S 9. Released glycan data for adalimumab as determined by xCGE-LIF. 37
Table S 10. Comparison of experimental means of glycans at the Fc/2 and HC level. 38

1. Supplementary figures

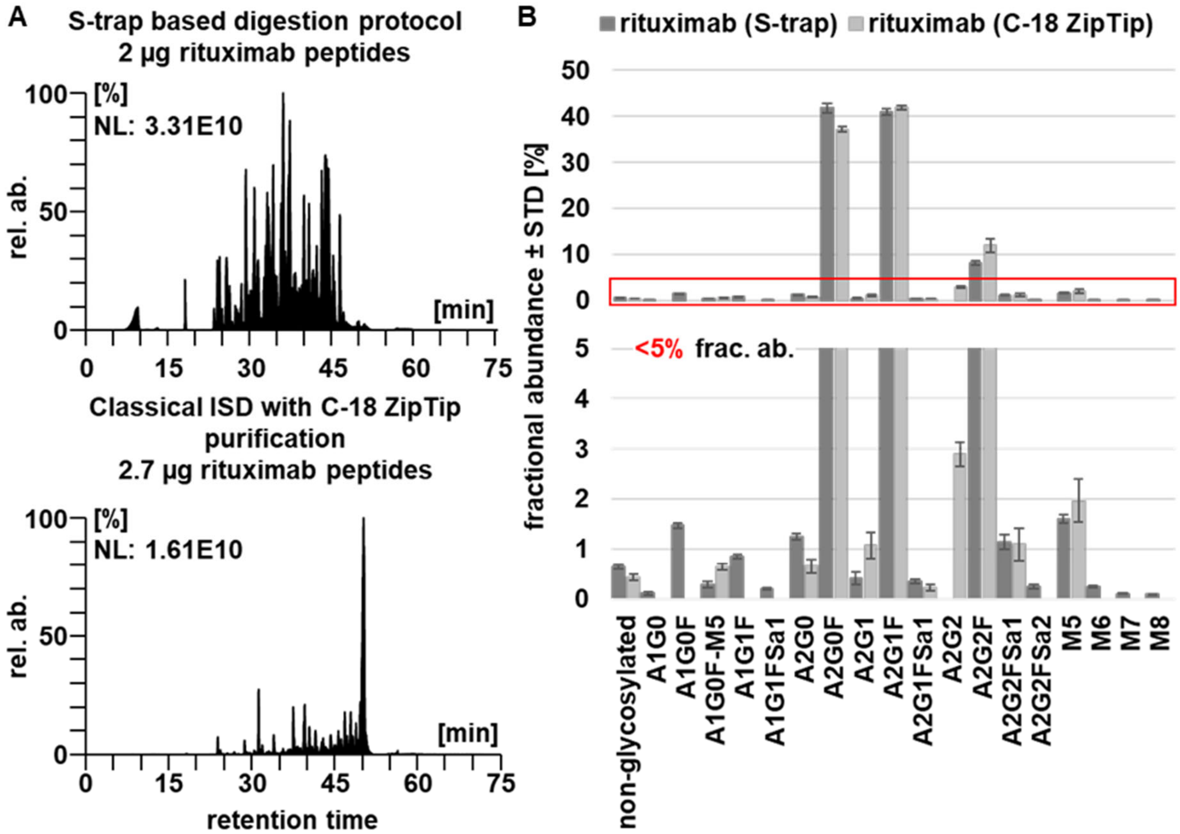

Fig. S 1. Comparison S-trap vs. classical ISD with C18 ZipTip peptide purification on the example of rituximab pre-measurements.
(A) Total ion current chromatograms of both digestion protocols. The top chromatogram was generated from rituximab prepared according to the S-trap protocol; the lower chromatogram shows data from rituximab peptides prepared with a classical ISD protocol with C18 ZipTip purification. y-axis, relative abundance in percent. **(B)** Skyline based quantification of glycopeptides. Especially minor abundant glycosylation variants (A1G0, A2G0F, A1G1F, A1G1FSa1, A2G2FSa2, M6-M8) got lost in the C18-ZipTip-based protocol. Only one variant is not present in S-trap measurements but the classical ISD protocol: A2G2. STD, standard deviation. Glycan nomenclature is explained in **Fig S13** (below), with the deviation that core fucosylation (F) is positioned at the end of the glycan name. The protocol for sample preparation as well as the discussion of data are provided in **chapter 3.1** of the supplementary material.

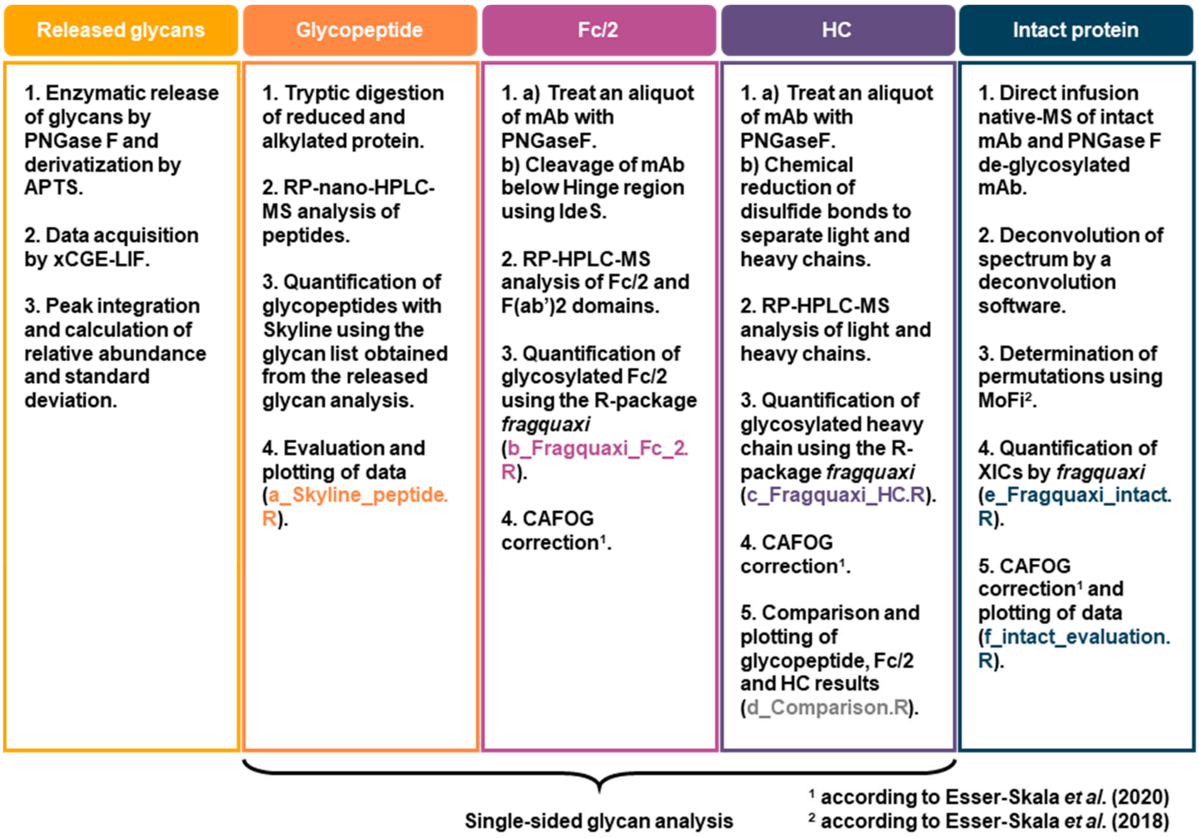

Fig. S 2. Simplified workflow overview for data generation and evaluation.
This figure describes data generation i.e., sample preparation and measurements in the lab as well as all necessary steps to evaluate the data. The results of the released N-glycan analysis served as basis for peptide mapping i.e., they were included in the glycopeptide quantification in Skyline. In addition, glycopeptide data were evaluated with Byonic and those results were combined with the list of glycopeptides to be quantified in Skyline. This list of glycans (observed at the peptide level) was also used to quantify glycosylation variants at the level of Fc/2 and heavy chain by utilization of the R-package fragquaxi. At the intact protein level, the software tool MoFi was used to annotate glycosylation variants to intact protein masses. In brief, the MoFi algorithm utilized quantitative data from the glycopeptide level and quantitative data from a zero-charge spectrum of the intact protein in order to calculate a permutation score providing quantitative information about the contribution of each glycan-combination to the intact mass. A detailed bioinformatic workflow description is provided in **Chapter 4** of this document. All raw data are available from Zenodo (https://doi.org/10.5281/zenodo.10455819).

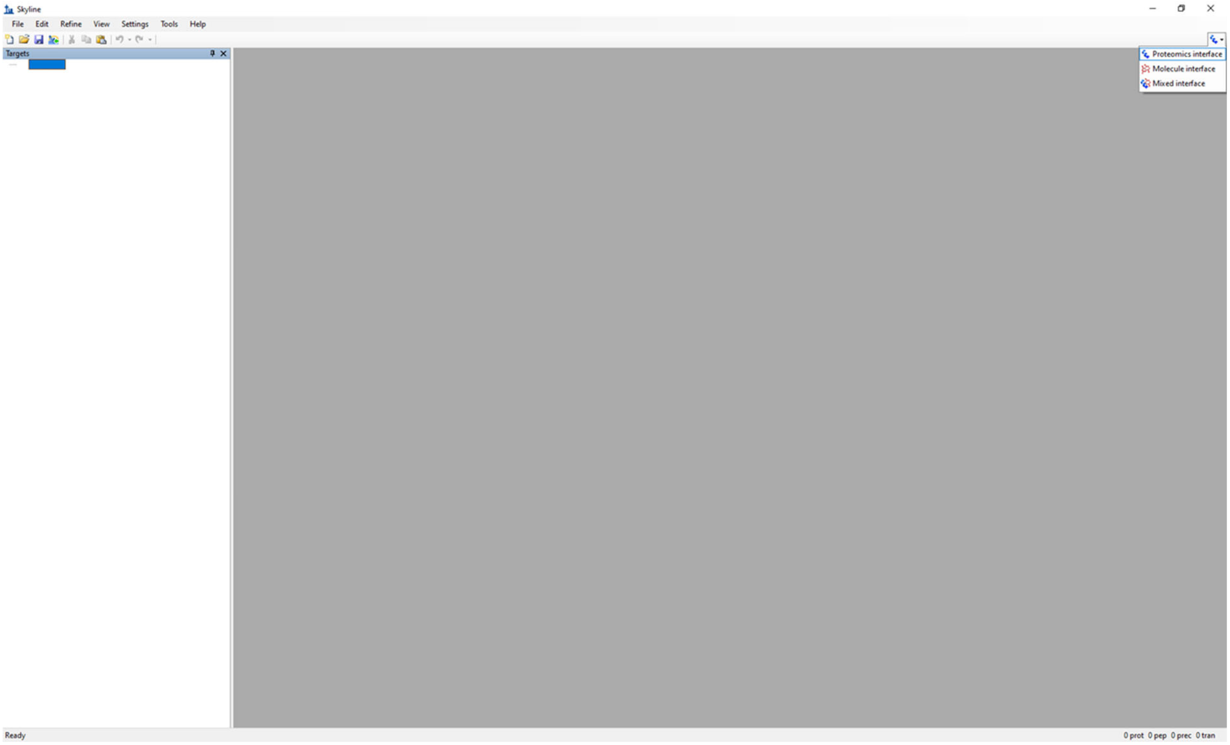

Fig. S 3. Skyline – proteomics interface.
A screenshot taken after Skyline has been opened. Proteomics interface should be selected for glycopeptide analysis.

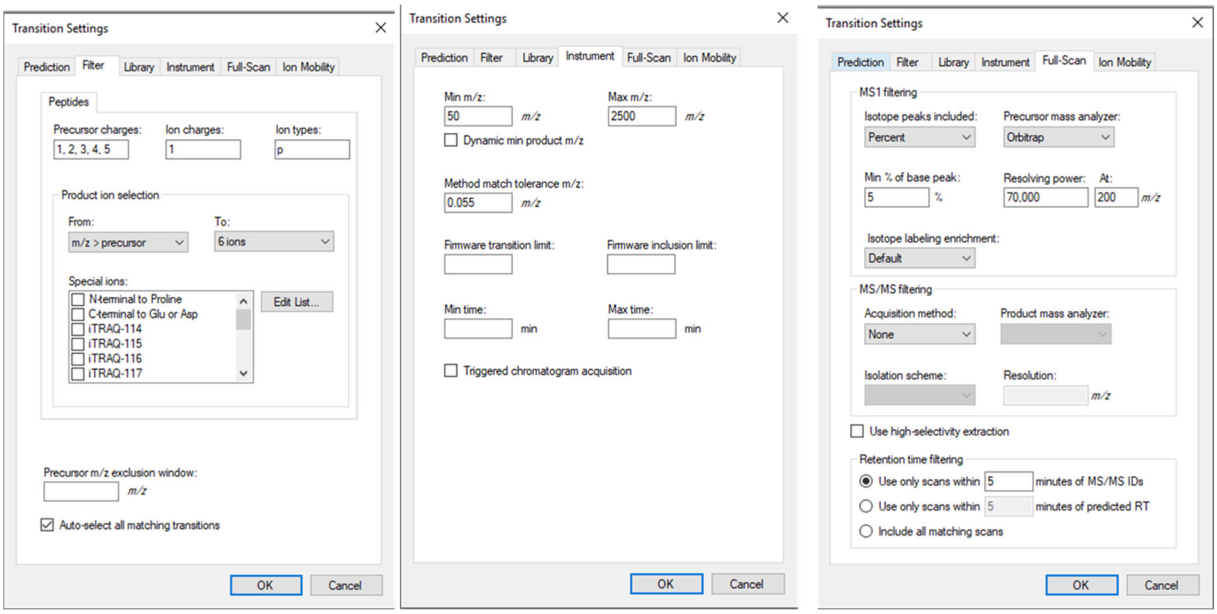

Fig. S 4. Skyline – edit transition settings.
Click *Settings* → *Transition Settings* to set the relevant parameters.

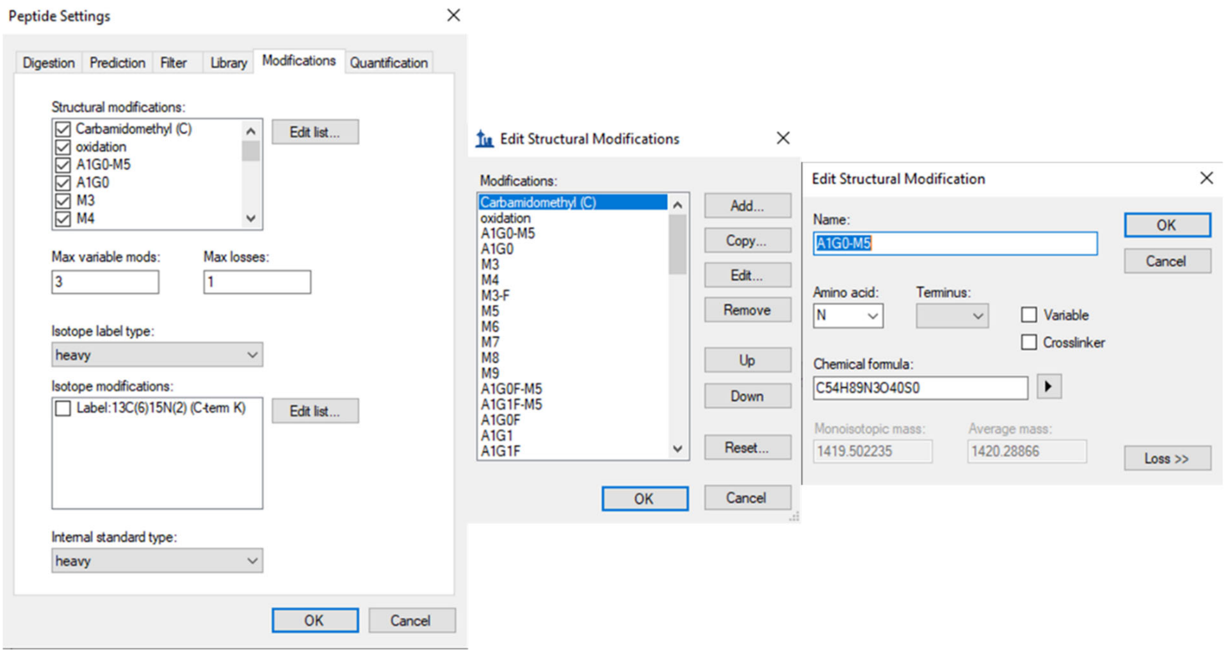

Fig. S 5. Skyline – edit peptide modifications.
Click: *Settings* → *Peptide Settings* → *Modifications* → *Edit list* → *Add* to generate a list of potential glycan structures or other peptide modifications.

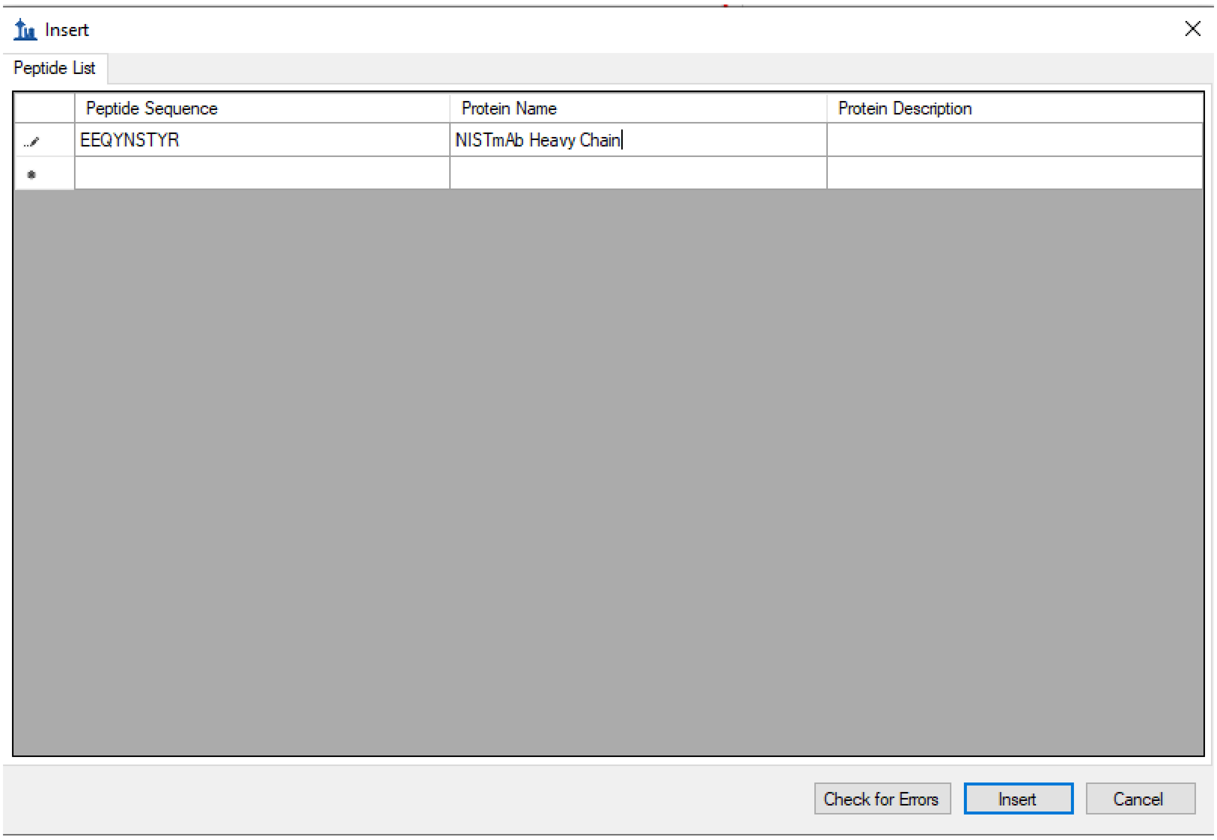

Fig. S 6. Skyline – edit peptide.
Click: *Edit* → *Insert* → *Peptides*. As an example, here, the glycopeptide of NISTmAb heavy chain is given.

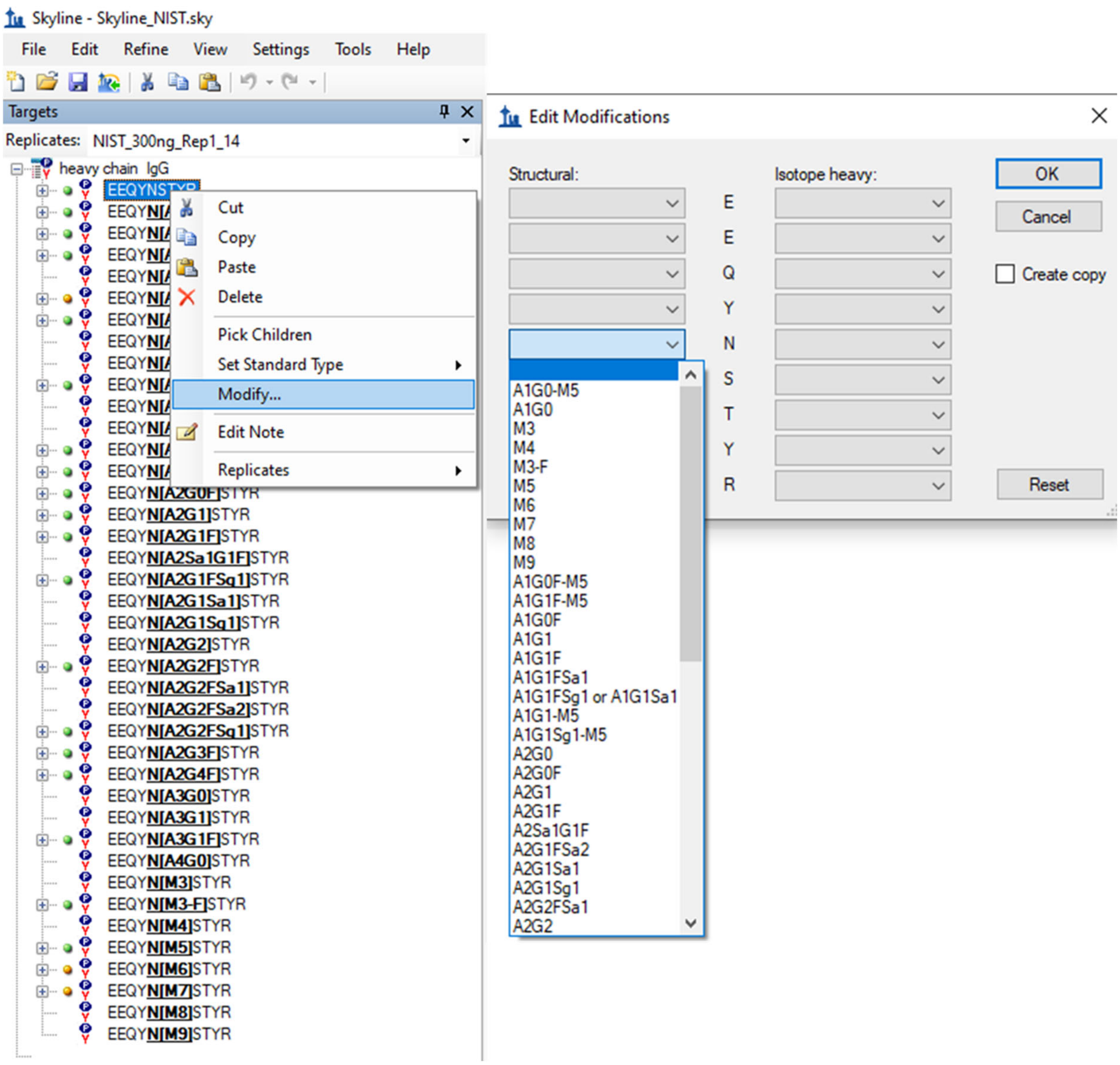

Fig. S 7. Skyline – edit peptide list with different modifications.
Right click on peptide → *Modify*. Then to each amino acid in the peptide sequence a modification can be added. After this step is done a list containing different modifications of the same peptide is provided under *Targets* in the Skyline window. It is important to tick *Create Copy*, otherwise the previous peptide will be overwritten.

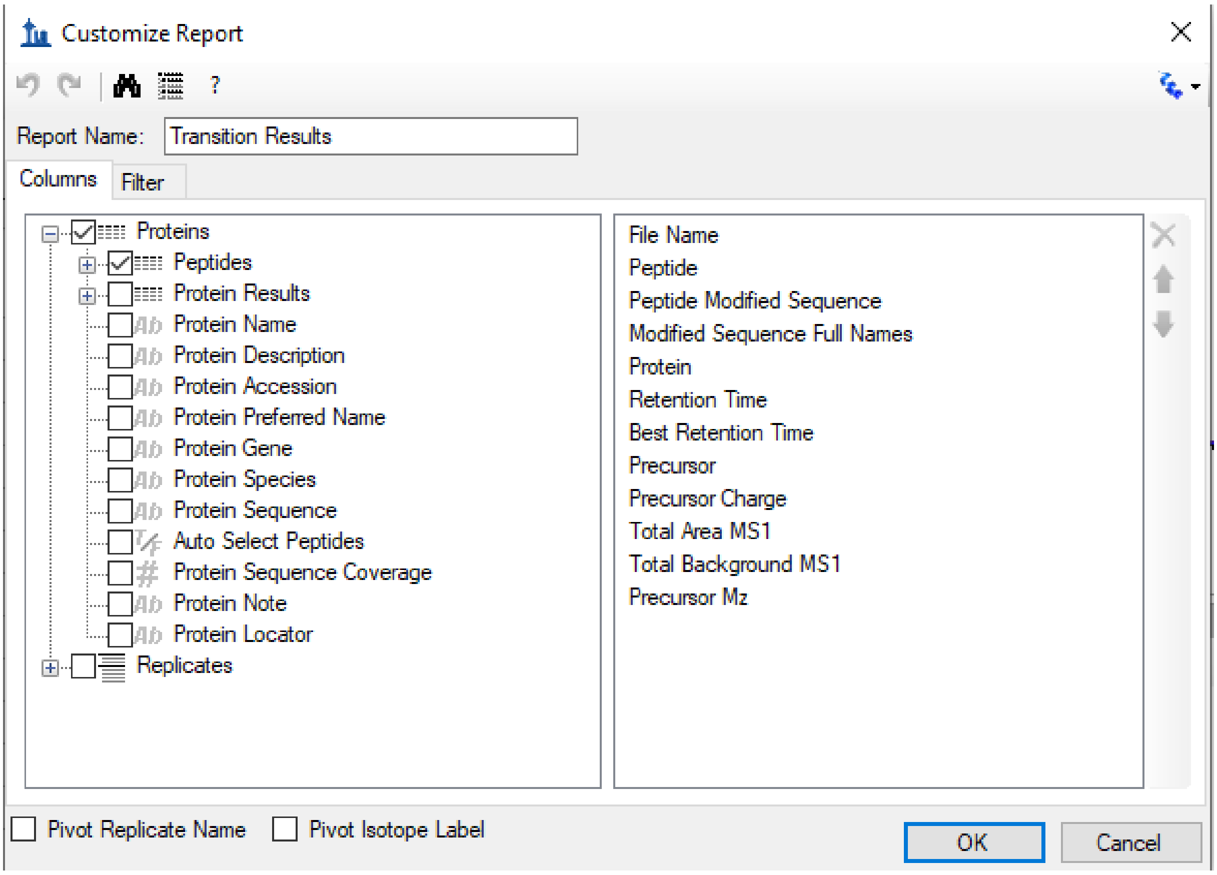

Fig. S 8. Skyline – edit report.
Click: *View* → *Document Grid* → *Reports* → *Edit Report*. Tick all the columns so that the same list as in this picture is present. The report name can be changed to the users own needs.

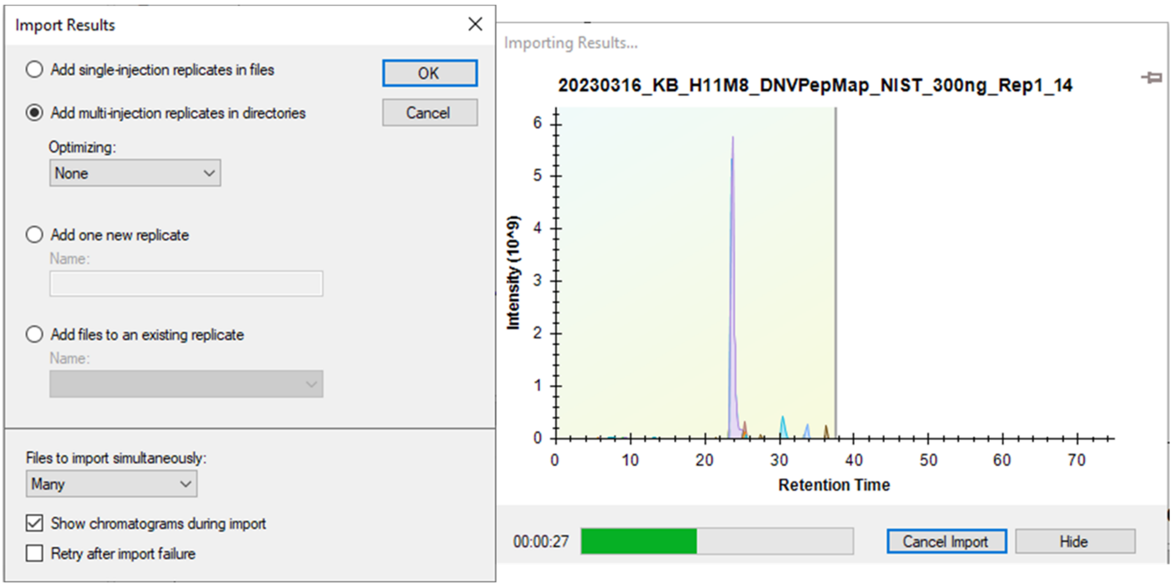

Fig. S 9. Skyline – import results, i.e., the MS raw files.
Click: *File* → *Import* → *Results*. Multiple files (i.e., technical replicates) can be loaded at the same time by selecting *add multi-injection replicates in directories*. By ticking *show chromatogram during import*, the "live" import can be observed. Note: before loading the results, the Skyline file can be saved as template and be re-used for other analyses, which saves time in setting up the list of modifications.

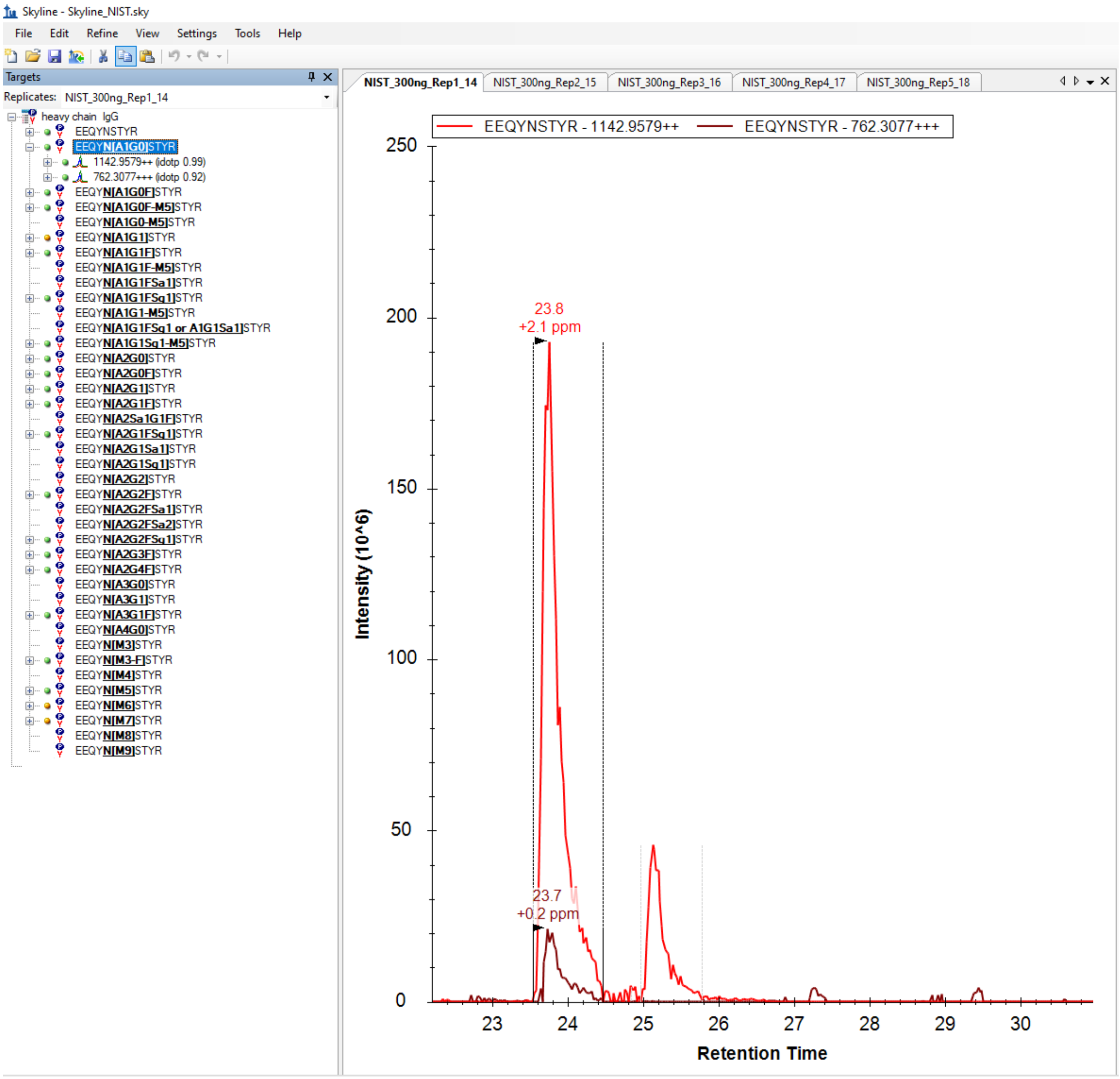

Fig. S 10. Skyline – XIC of different charges.
Here for the observed peptide the most abundant charge state (in red) is the doubly charged peptide. However, the 3x-charged peptide is present and can be quantified.

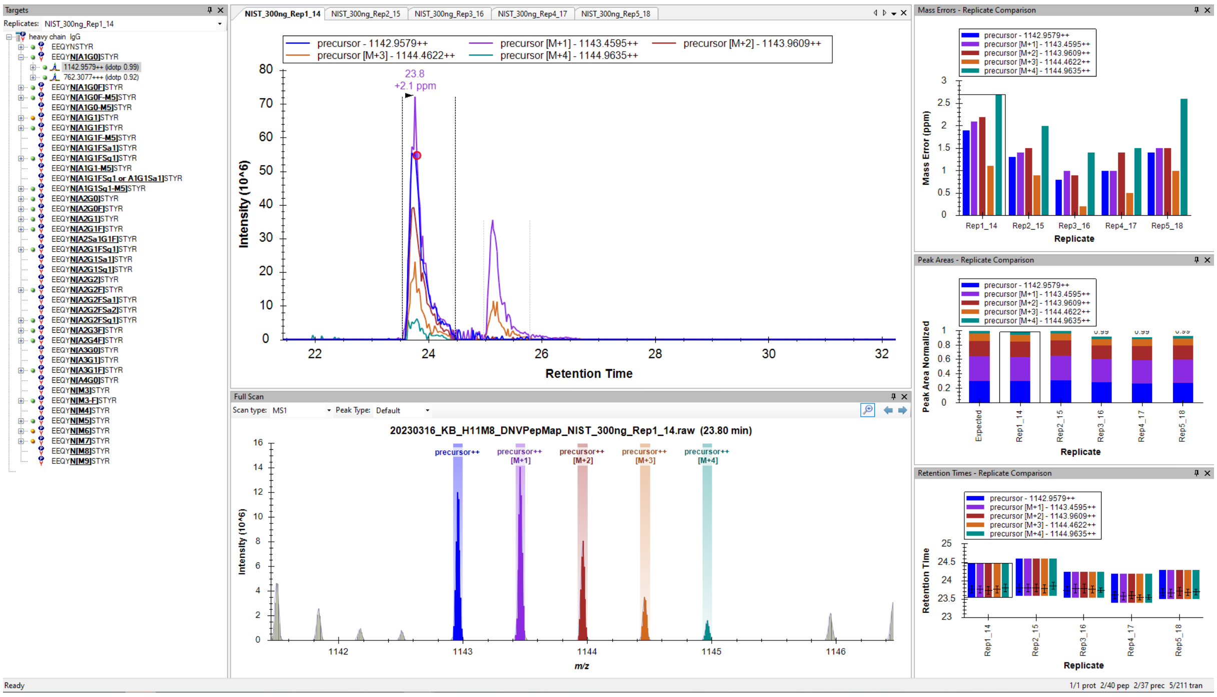

Fig. S 11. Skyline – observe results.

Once results were imported, the data can be observed (Precursor ions, Isotopic patterns etc.) for all replicates. This screenshot shows on the left the list of modifications of the glycopeptide (*targets*), on the top middle the chromatogram (specifically the extracted ion current chromatogram for the A1G0 modified peptide in the first technical replicate), and on the bottom middle the corresponding *full scan* with highlighted isotopes. In this screenshot the modification A1G0 is selected. Two charge states of the glycopeptide with this modification were observed (other charge states were deleted and are thus not shown here). For each charge state, isotopes were highlighted in the full scan. If the theoretical isotope pattern is fitting the real data, the charge state of that peptide will be considered for quantification. By scrolling below the x-axis of the chromatogram, the time window for the extracted ion current chromatogram chosen for quantification can be adjusted – the window for integration is shown as dashed vertical lines and above the most intense isotope a black arrow marks the peak to be quantified. On the right side of this screenshot a *replicate comparison* is provided for the selected 2+ charged A1G0 peptide including mass error, peak area, and retention time. The replicate comparison is obtained by clicking the following sequence: View → Retention times → replicate comparison; View → Peak areas → replicate comparison; View → Mass Errors → replicate comparison.

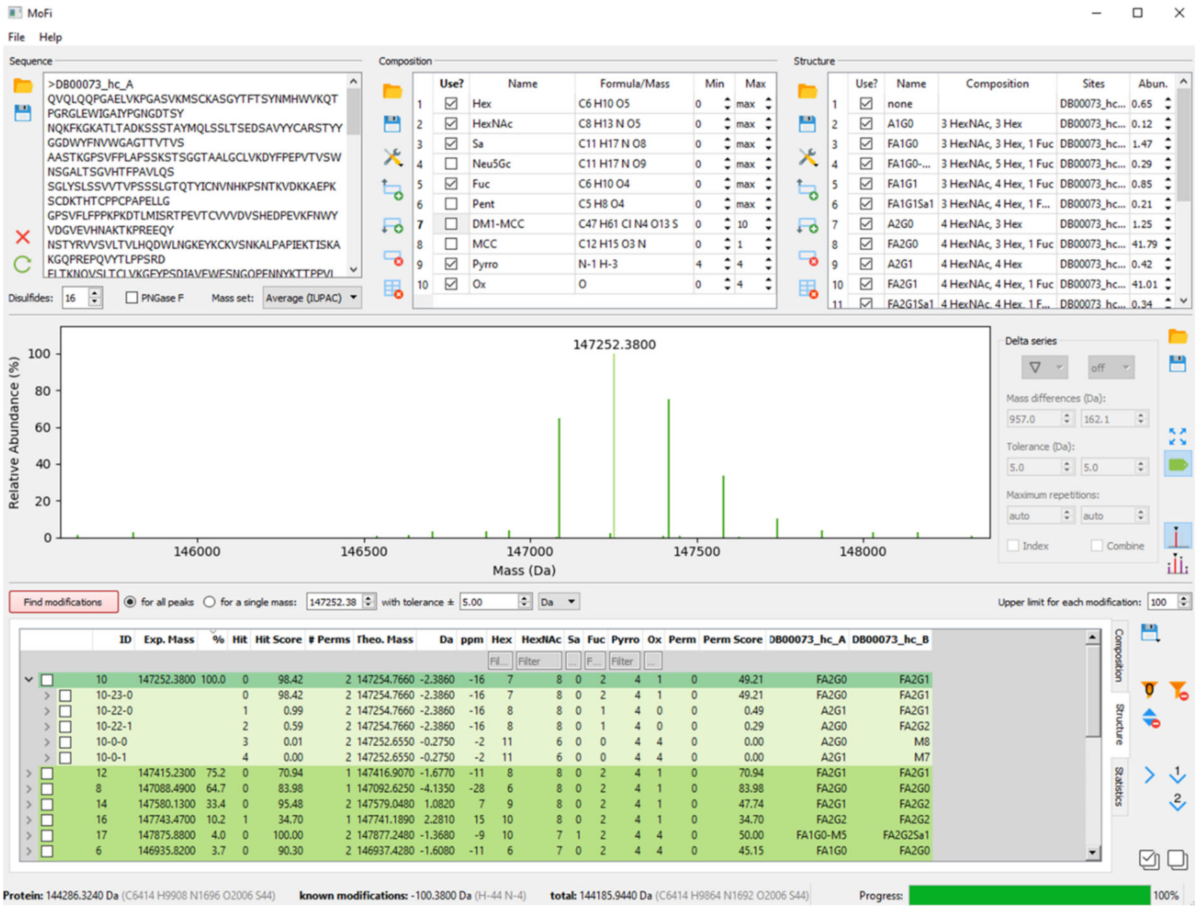

Fig. S 12. Graphical user interface (GUI) of MoFi [24]. The example is provided on rituximab. Input includes protein **Sequence**, PTM **Composition**, PTM **Structure**, and protein masses of the deconvoluted **Spectrum**.

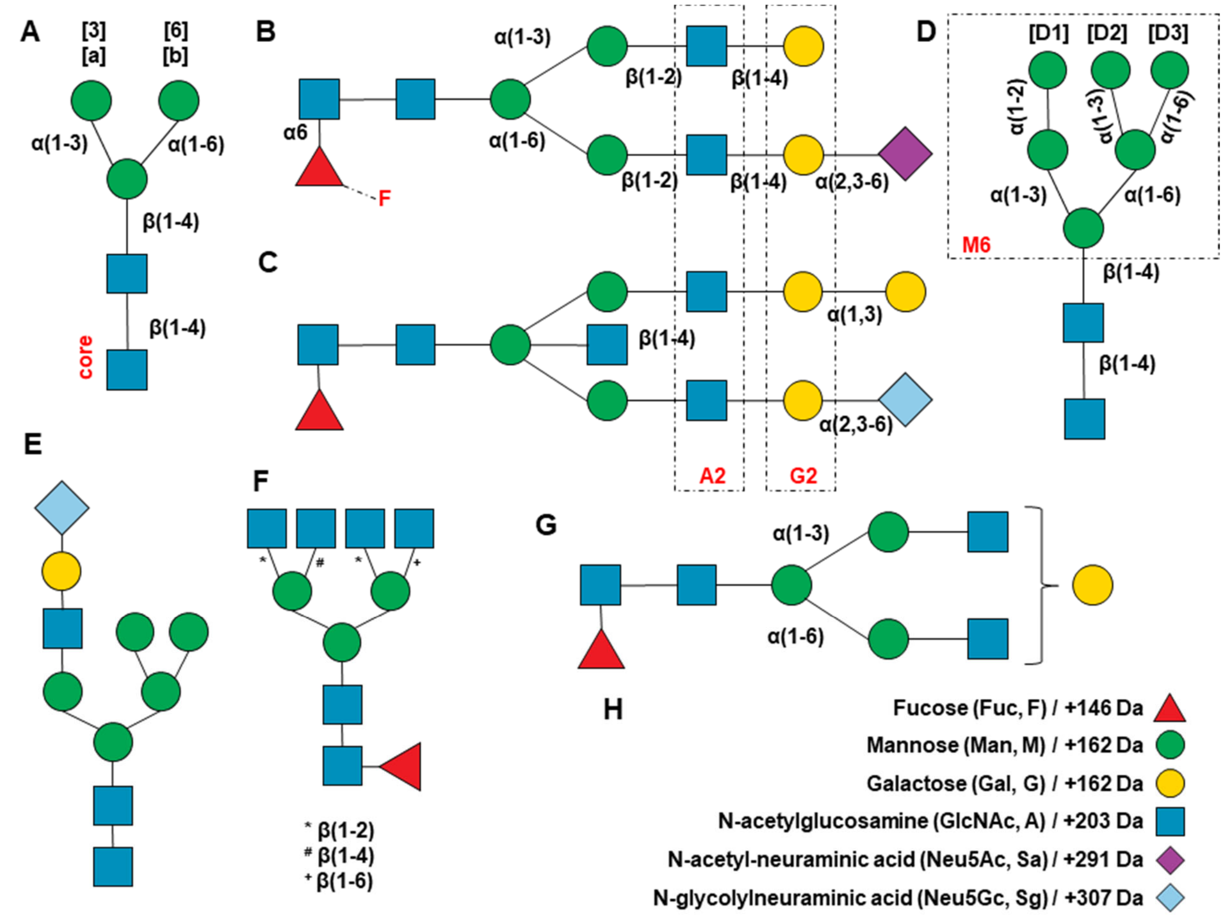

Fig. S 13. Symbol nomenclature of *N*-glycans based on references [1,2].
(A) The **core structure** of *N*-glycans is represented as an M3 species, comprising two GlcNAc units and three mannose units (mannotriose-di-N,N'-acetylchitobiose core structure). Antennae/arms' positions are denoted by squared brackets. For instance, extension of the α(1-3) arm (left arm) to oligomannose species is indicated as [a], while in complex-type glycans, it is represented as [3]. The linkage between two sugar residues is indicated using curved brackets, e.g., α(1-3). **(B)** Depicts a **complex-type** glycan with α(1,6)-linked core-fucosylation (F) and Neu5Ac (Sa) derivatization on the α(1-6) arm: FA2G2Sa1[6]. The MS-detectable glycan is abbreviated as FA2G2Sa1, ignoring the linkage position. **(C)** Shows a complex-type glycan with a **bisected** GlcNAc (B), an immunogenic α-Gal on the α(1-3) arm [3], and Neu5Gc on the α(1-6) arm. The abbreviated form is FA2BG2αG1[3]Sg1[6], with MS-detection shortened to FA3G3Sg1. The sialic acid can be either α(2,3)- or α(2,6)-linked to the galactose, abbreviated as α(2,3-6). **(D)** Represents an **oligomannose-type** glycan with the abbreviation M6. The number of mannoses is indicated after M. If further derivatization to M7 occurs, the position of the seventh mannose is indicated by M7[D1], located on the α(1-3) arm on the left side. **(E)** Depicts a **hybrid-type** glycan with a complex-type structure on the α(1-3) arm and an oligomannose structure on the α(1-6) arm: M5-A1G1Sg1(2,3-6)[3]. This is represented by a curved bracket. **(F)** Shows a complex-type glycan with **multiple antennae** (A) modifying the core mannoses: FA4G0. **(G)** By mass spectrometry, stereoisomers cannot be distinguished. Consequently, a terminal galactose can be located on both arms, the α(1-3) or the α(1-6). Annotations based on mass spectrometry therefore refer to one or both isomers. **(H)** Provides a legend with information about monosaccharide names, abbreviations, and their masses.

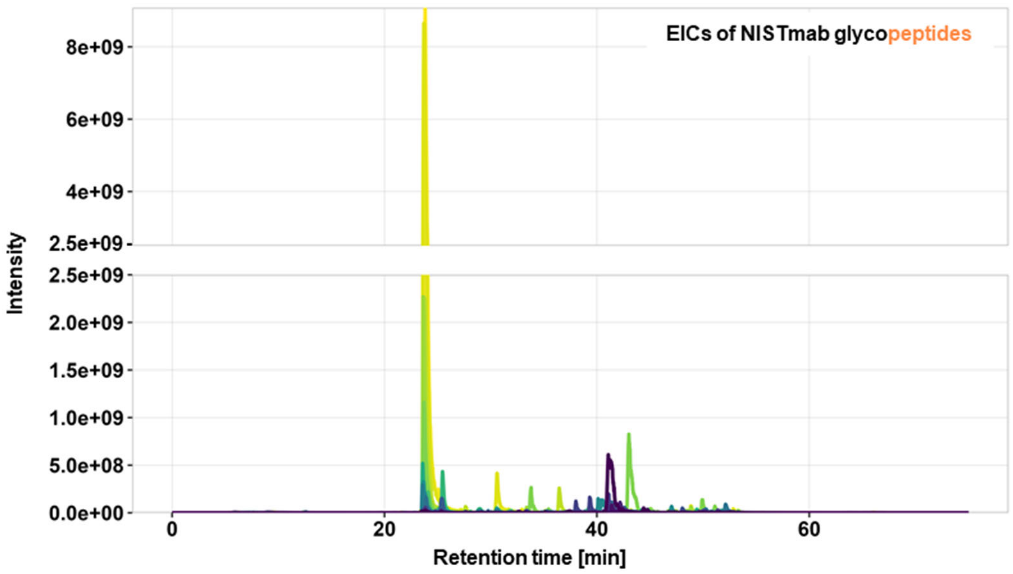

Fig. S 14. Extracted ion current chromatograms (EICCs) of the glycopeptide EEQYNSTYR of NISTmAb.
A detailed zoomed-in view of the glycopeptide region (t_R = 23.5-25.5 min) can be found in **Fig. 5A** in the main manuscript. t_R, retention time.

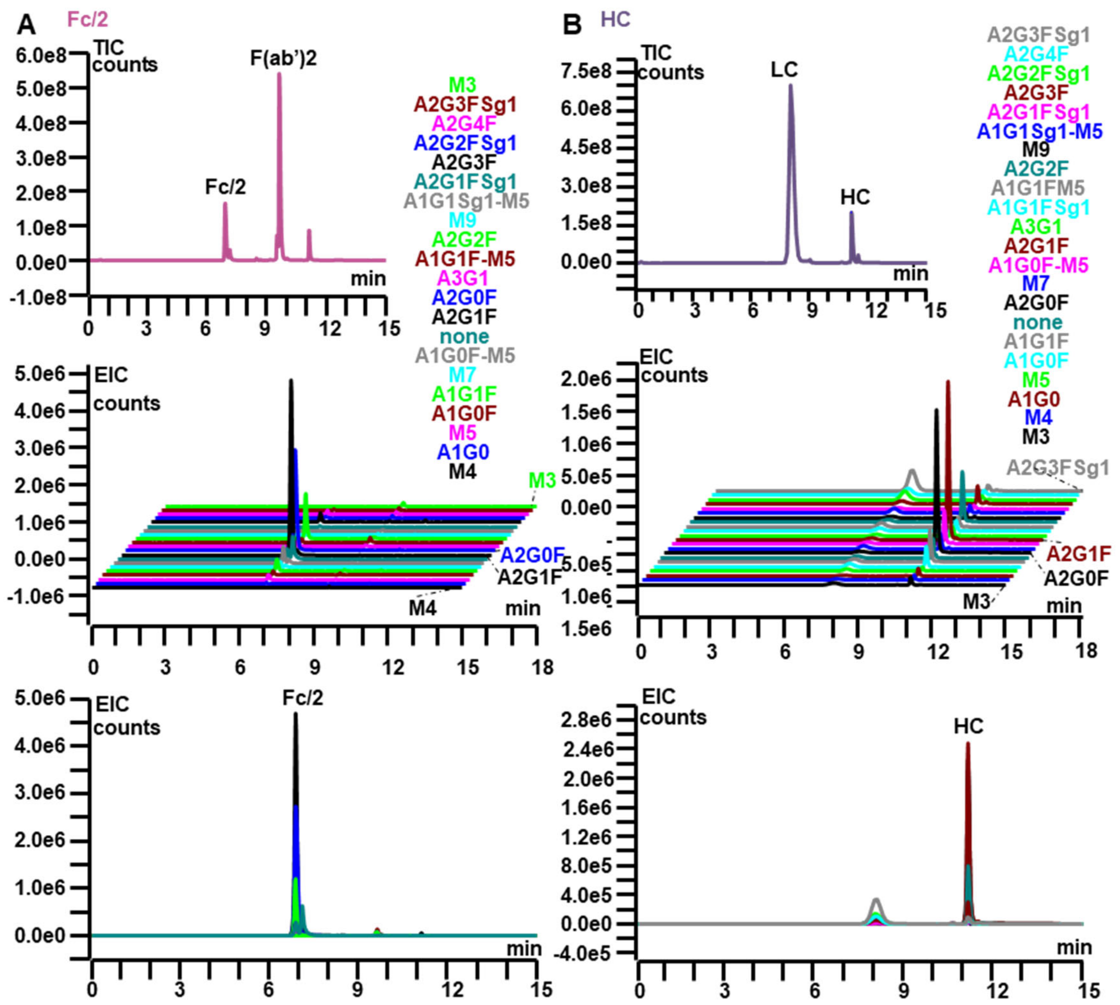

Fig. S 15. Total Ion Current Chromatograms (TICs) and Extracted Ion Current Chromatograms (EICCs) for IdeS-digested **(A)** and reduced **(B)** NISTmAb.

The top panel displays the TICC, while the middle and bottom panels offer a stacked and overlaid comparison of the EICCs. The glycans used for generating the EICCs are color-coded and ordered to correspond with the chromatograms. Zoomed-in views of these EICCs can be found in **Fig. 5B-C** in the main manuscript. Glycan nomenclature is explained in **Fig. S13**.

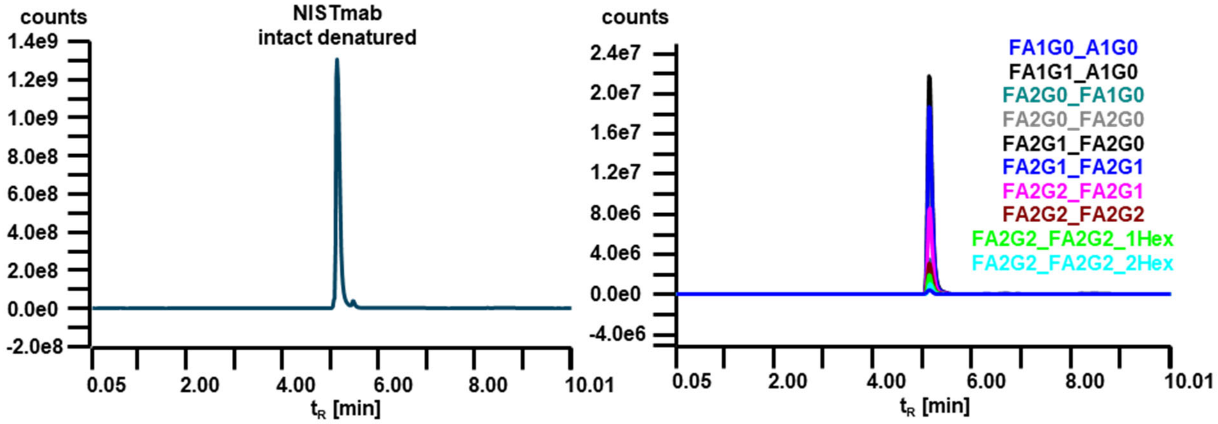

Fig. S 16. Total Ion Current Chromatogram (TICC, left) and Extracted Ion Current Chromatogram (EICC, right) of intact NISTmAb.

Spectral data was obtained through RP-LC-MS, providing denatured NISTmAb mass spectrometry data. Per *m/z* peak one glycan pair was used to calculate the EICCs. For closer examination, a zoomed-in view is provided in **Fig. 5D** of the main manuscript. Glycan nomenclature is explained in **Fig. S13**.

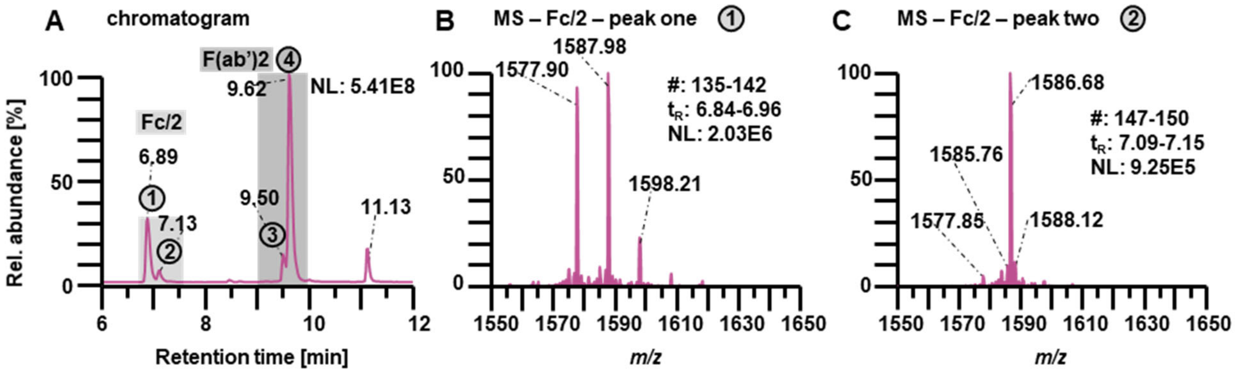

Fig. S 17. Separation of IdeS-generated Fc/2 and F(ab')2 species *via* RP-HPLC and the corresponding mass spectra (NISTmAb).

(A) The chromatogram displays the separation of Fc/2 (light grey rectangle), which elutes first, and F(ab')2 variants (dark grey rectangle). Fc/2 elutes at around t_R ~ 7 min, eluting as two distinct peaks labeled as (1) and (2). The F(ab')2 domain follows, eluting between eight and ten minutes, also as two peaks. **(B)** In the mass spectrum of the first Fc/2 peak, the underlying glycosylation variants are revealed. **(C)** The mass spectrum of the second Fc/2 peak showcases the main *m/z*-peak, corresponding to the non-glycosylated mAb species, highlighting the glycan-dependent separation of Fc/2. t_R, retention time; NL, normalized intensity value; #, number of scans.

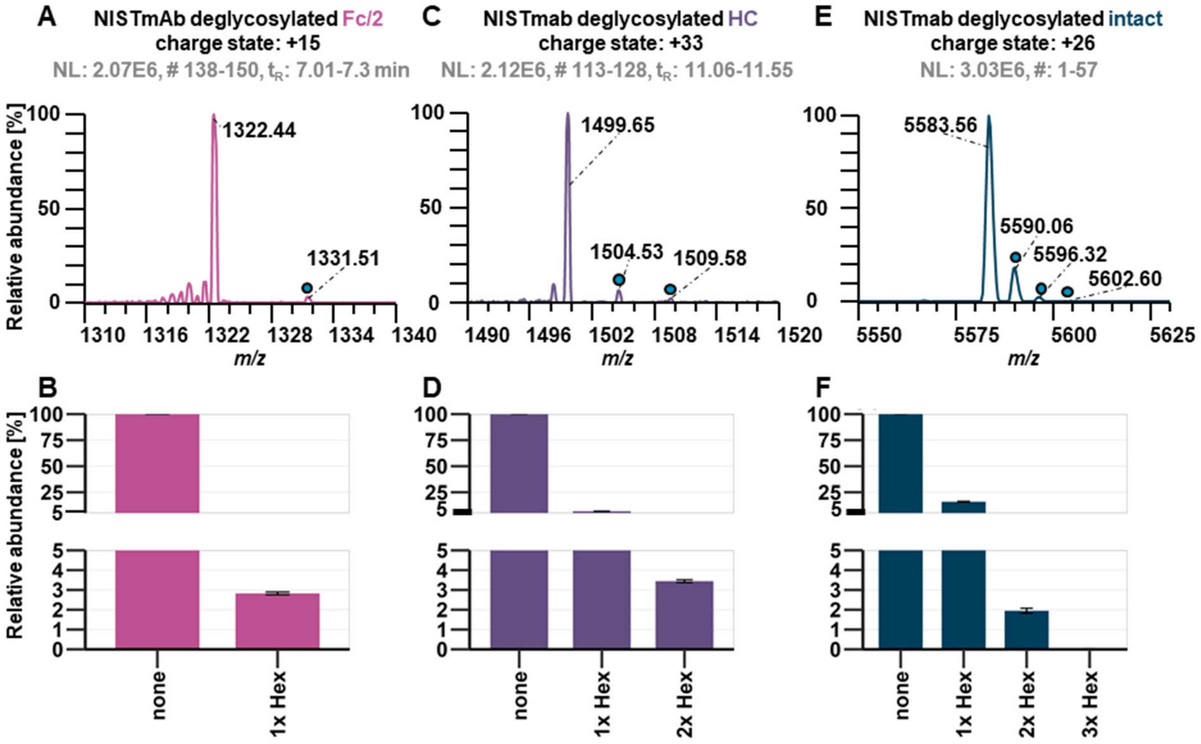

Fig. S 18. Raw spectra of PNGase F treated NISTmAb and quantification of glycation. **(A, B)** Glycation assessment at the Fc/2 level; **(C, D)** Glycation assessment at the HC (heavy chain) level; **(E, F)** Glycation assessment at the intact NISTmAb level. The most prominent *m/z*-peak corresponds to the non-glycated species ("none"), while *m/z*-peaks resulting from glycation are annotated with blue circles (representing glucose). Quantification was achieved using EICCs (Extracted Ion Current Chromatograms) with *fragquaxi*, applying specific modification parameters such as C-terminal lysine removal and fixed modification for loss of H_2O (-18 Da), accounting for pyroglutamic acid formation from glutamine. The y-axis represents relative abundance in percentage, and error bars depict standard deviation (%). These values were computed from five technical replicates for Fc/2 and HC, and six technical replicates for intact NISTmAb acquired under native conditions. t_R, retention time.

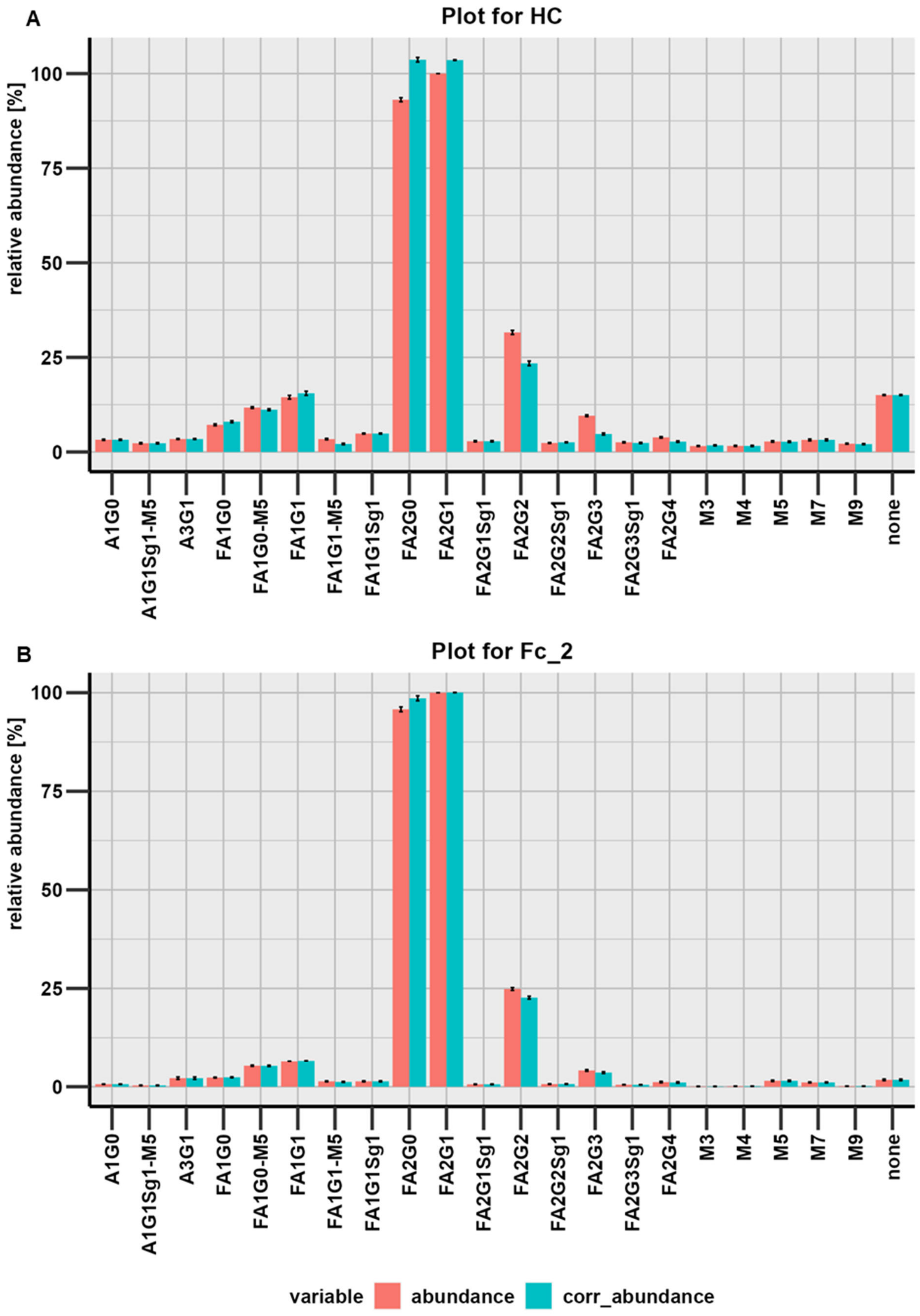

Fig. S 19. CAFOG correction for glycation bias in NISTmAb.
In **(A)** corrected glycan abundances for HC-level, and in **(B)** corrected glycan abundances for Fc/2 level are shown. Glycan nomenclature is explained in **Fig. S13**.

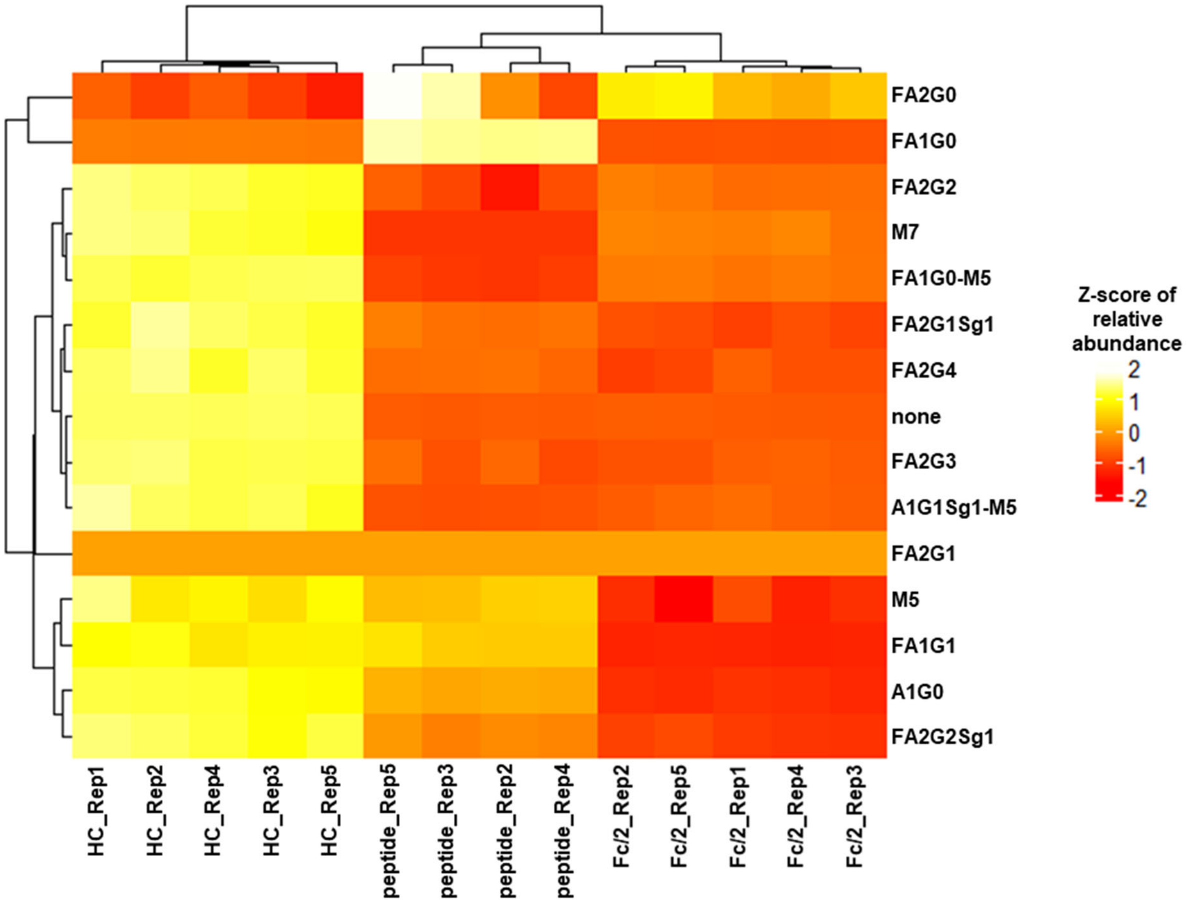

Fig. S 20. Heatmap showing relative abundances of glycan-moieties at different structural glyco-(poly)peptide levels of NISTmAb.
Note: only glycans present at all structural levels are included. Heat-map depicting the z-score. Glycan nomenclature is explained in **Fig. S13**.

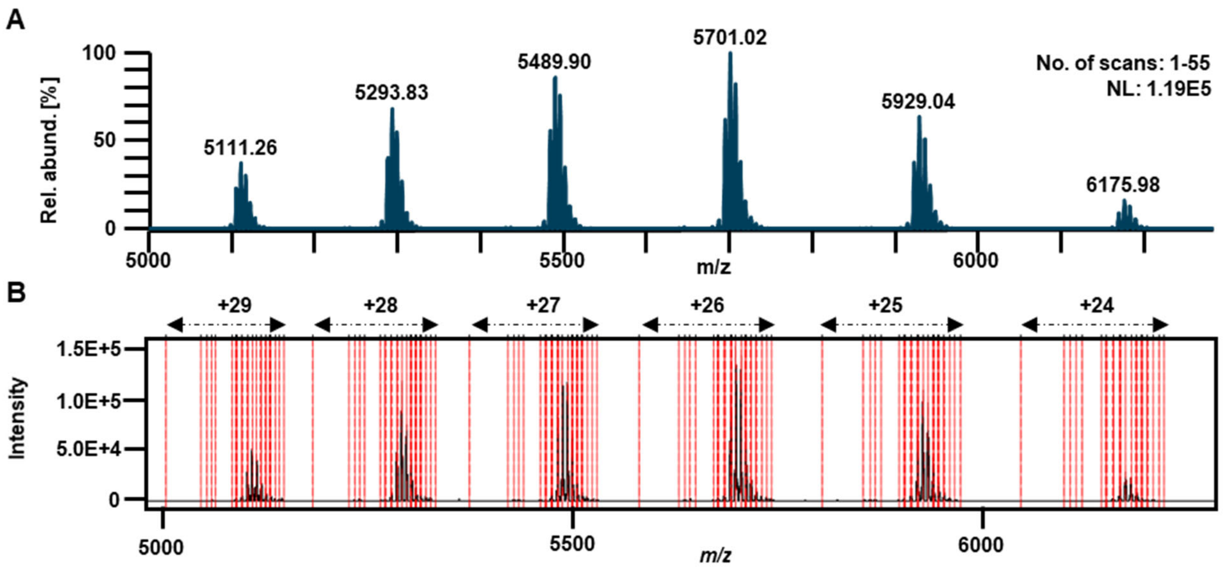

Fig. S 21. Raw spectrum of intact NISTmAb acquired by native MS.
(A) Raw spectrum of the fifth technical replicate of intact NISTmAb averaged from 55 scans. **(B)** The same spectrum with peak annotations visualized in R using the package *fragquaxi*. The charge state ladder ranges from 29+ to 24+. None of the charge states is overlapping. For closer examination of the spectrum with glycan annotations, please refer to **Fig. 7** in the main manuscript, which includes a zoomed-in view of the z = 27+ charge state.

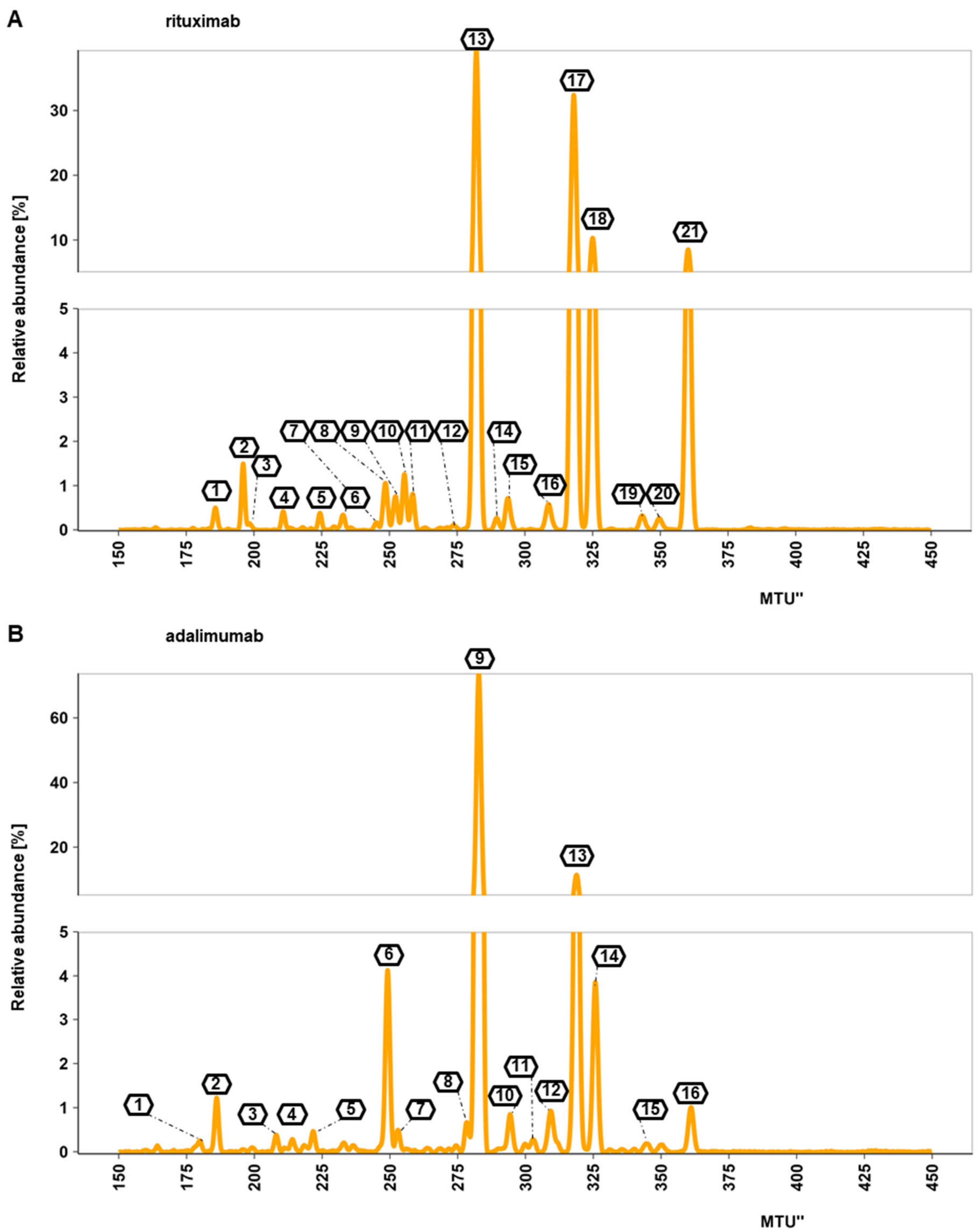

Fig. S 22. xCGE-LIF glycofingerprints (t_{mig} aligned and signal normalized electropherograms) of **(A)** rituximab and **(B)** adalimumab.

The annotated glycan structures can be found in **Fig. S23** and **Fig. S24** for rituximab and adalimumab, respectively.

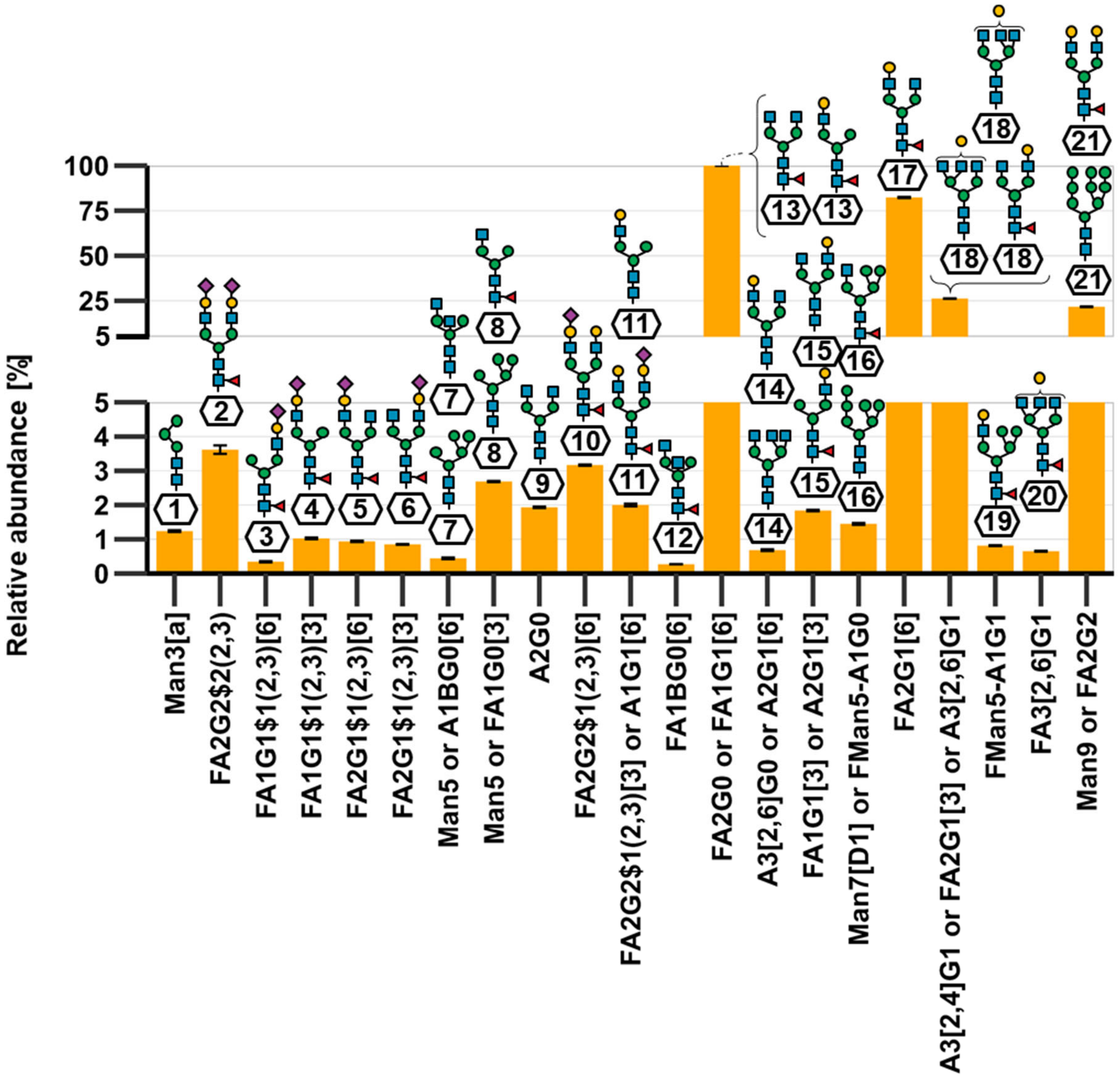

Fig. S 23. Relative quantification of released glycans from rituximab, analysed by xCGE-LIF. Peak numbers refer to peaks in **Fig. S22A**. Corresponding raw data are listed in **Table S8**. Glycan nomenclature is explained in **Fig. S13**. Deviation from glycan nomenclature in **Fig S13**: $, N-acetylneuraminic acid.

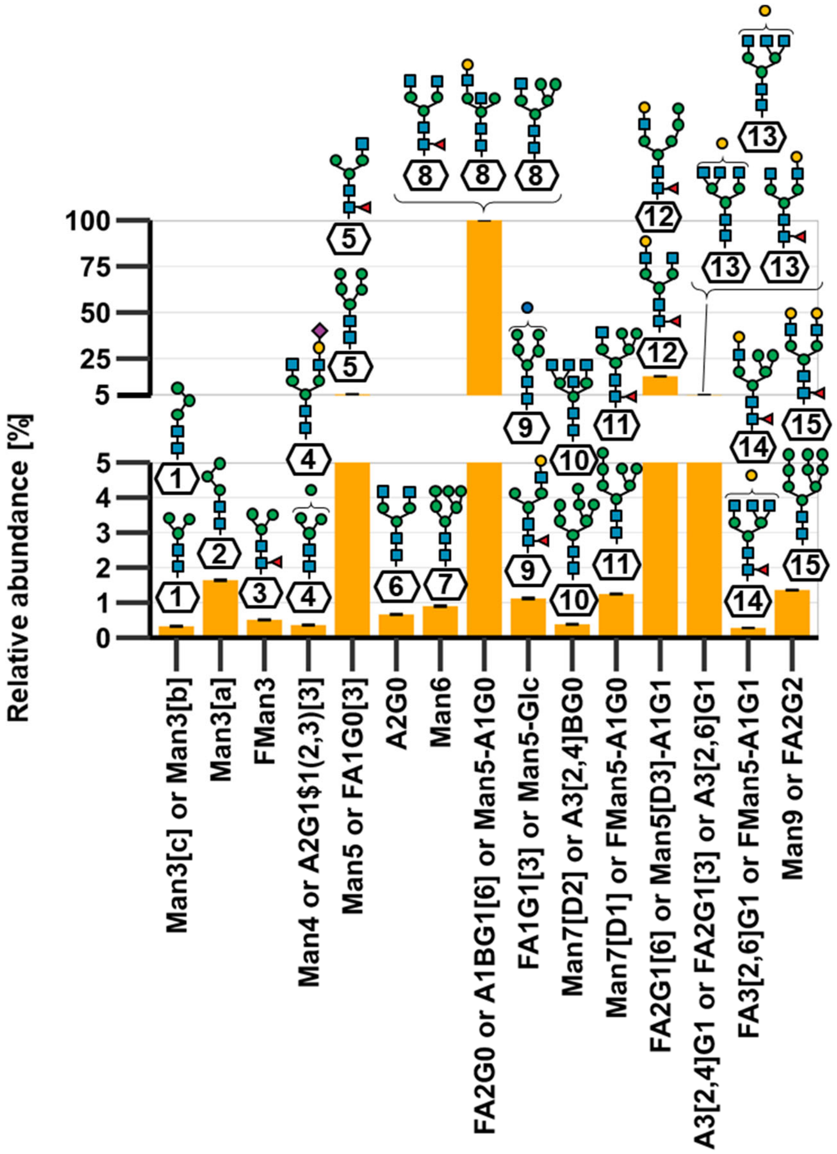

Fig. S 24. Relative quantification of released glycans from adalimumab, analysed by xCGE-LIF.

Peak numbers refer to peaks in **Fig. S22B**. Corresponding raw data are listed in **Table S9**. Glycan nomenclature is explained in **Fig. S13**. Deviation from glycan nomenclature in **Fig S13**: $, N-acetylneuraminic acid.

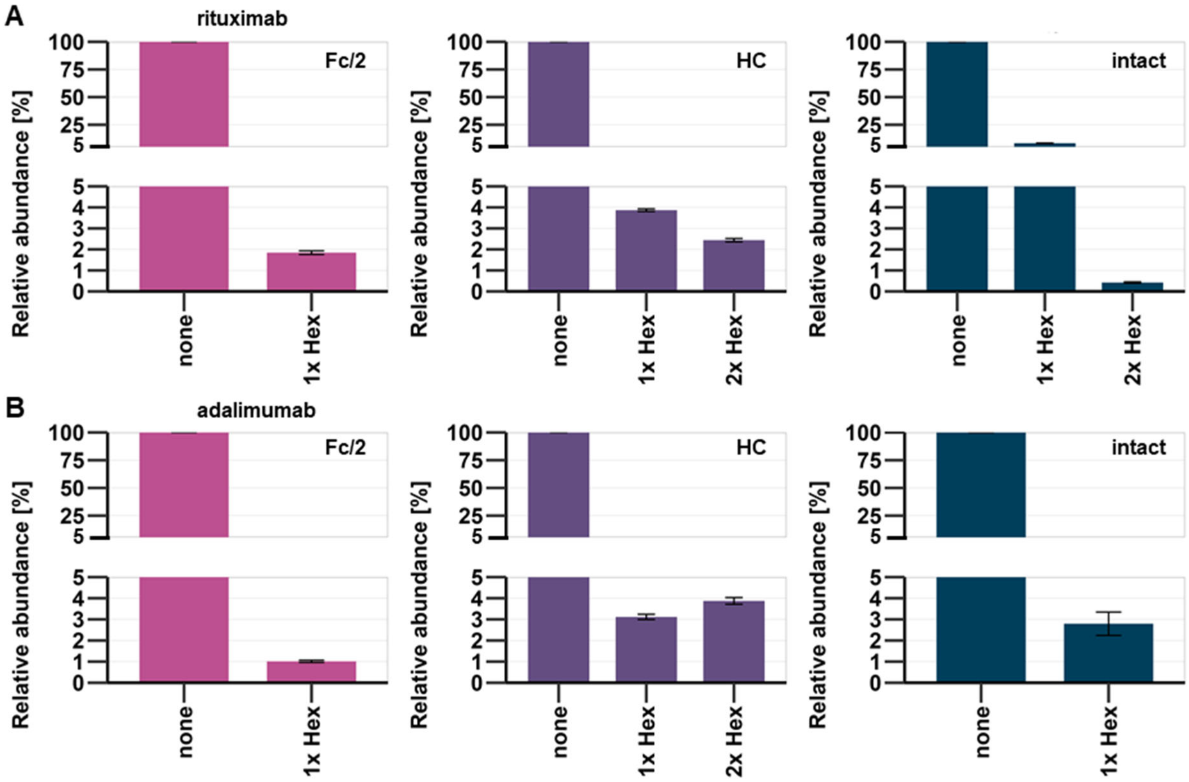

Fig. S 25. Analysis of glycation.
Glycation is shown for **(A)** rituximab and **(B)** adalimumab at the Fc/2 level after IdeS digestion (left), HC level upon disulfide bond reduction (middle), and at the intact protein level (right).

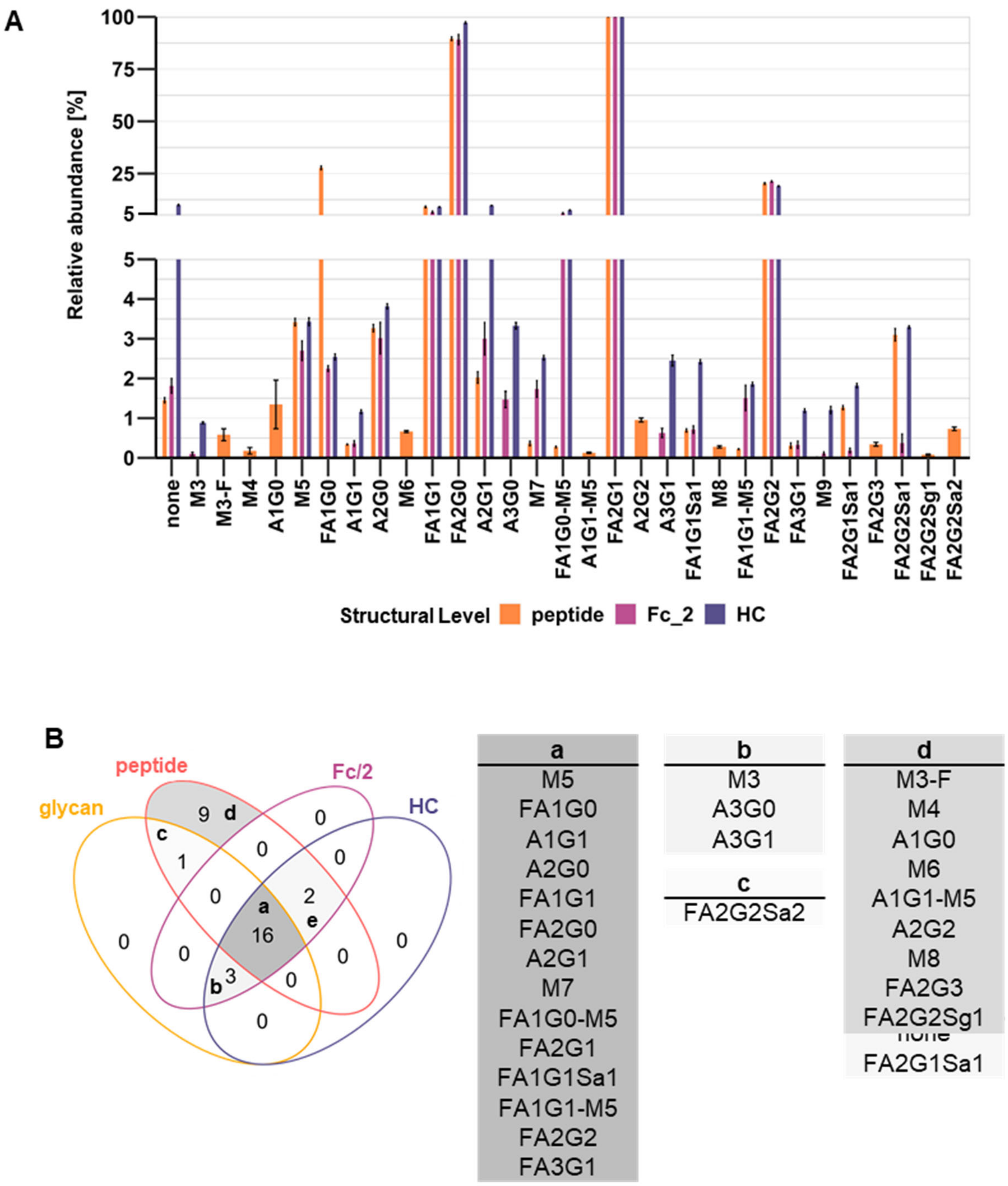

Fig. S 26. Quantitative **(A)** and qualitative **(B)** comparison of rituximab-glycan-moieties.
(A) Relative abundance is plotted for peptide and both subunit levels. Error bars represent standard deviation. Thickness of bars indicate number of occurrences of a glycoform at i.e., all, two or one structural level. **(B)** Venn diagram qualitatively shows the number of glycans occurring at diverse structural levels. Letters show the underlying glycans in the tables to the right of the Venn diagram. Glycan nomenclature is explained in **Fig. S13**.

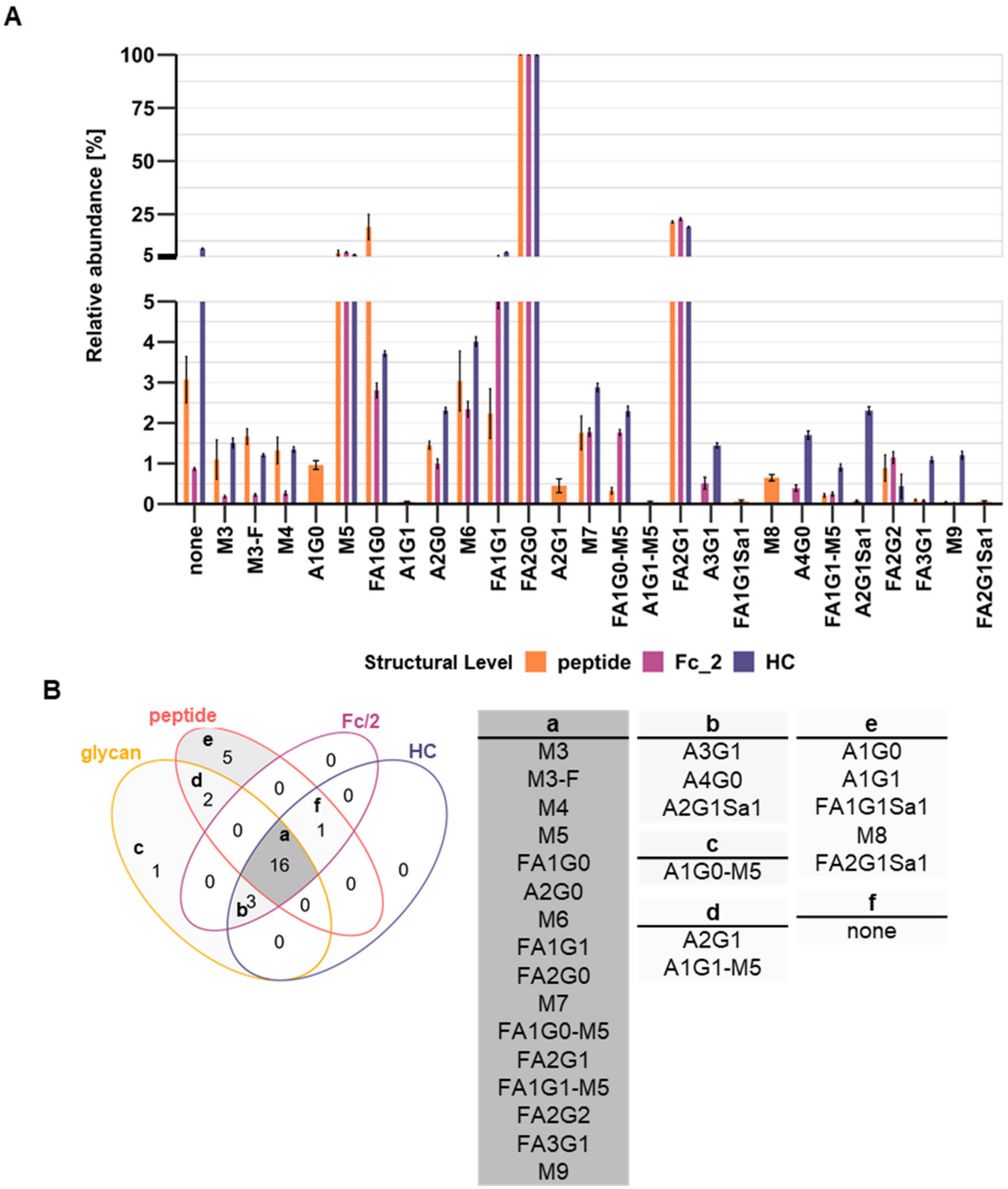

Fig. S 27. Quantitative **(A)** and qualitative **(B)** comparison of adalimumab-glycan-moieties. **(A)** Relative abundance is plotted for peptide and both subunit levels. Error bars represent standard deviation. Thickness of bars indicate number of occurrences of a glycoform at i.e., all, two or one structural level. **(B)** Venn diagram qualitatively shows the number of glycans occurring at diverse structural levels. Letters show the underlying glycans in the tables to the right of the Venn diagram. Glycan nomenclature is explained in **Fig. S13**.

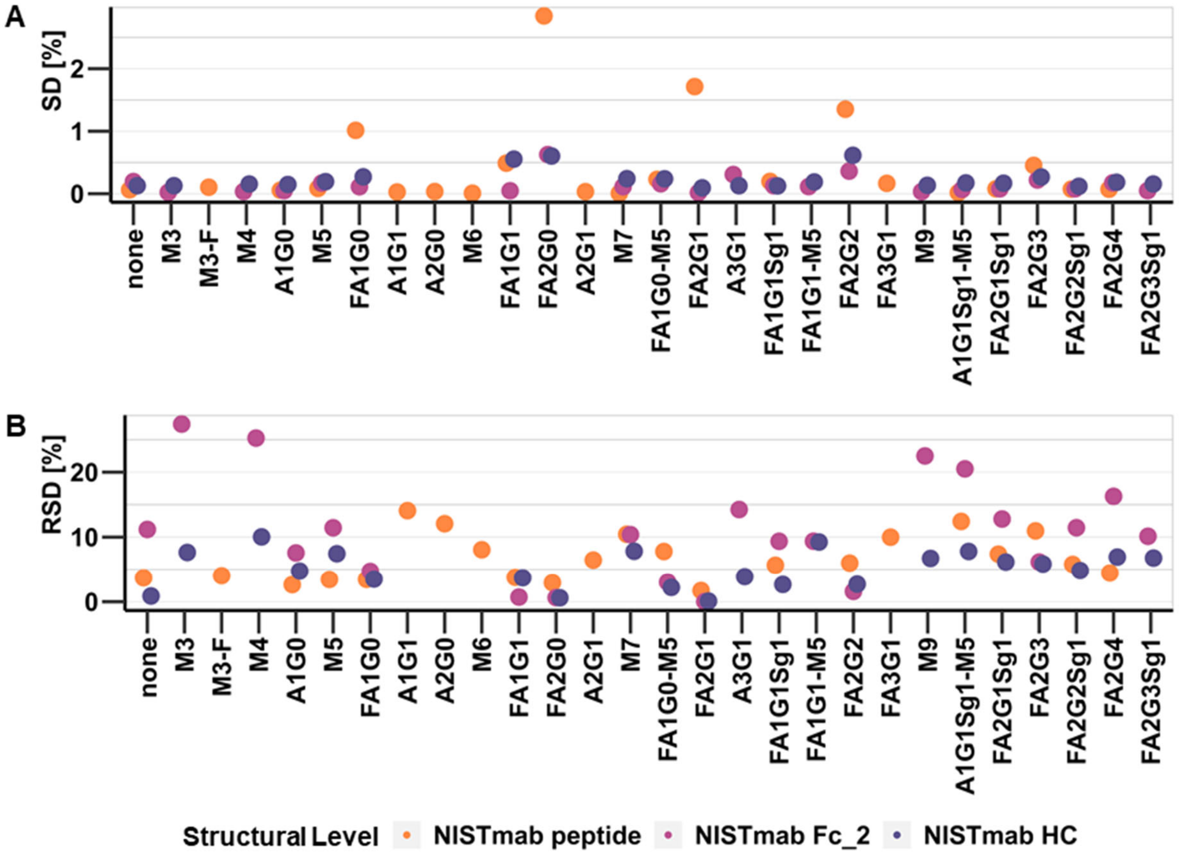

Fig. S 28. Visualization of statistics for NISTmAb.
Standard deviation [%] **(A)** and relative standard deviation [%] **(B)** plotted for each glycovariant at different structural levels for NISTmAb: peptide, Fc/2 and HC. Standard deviation (SD) and relative SD (RSD) were determined from five technical replicates. Glycan nomenclature is explained in **Fig. S13S**.

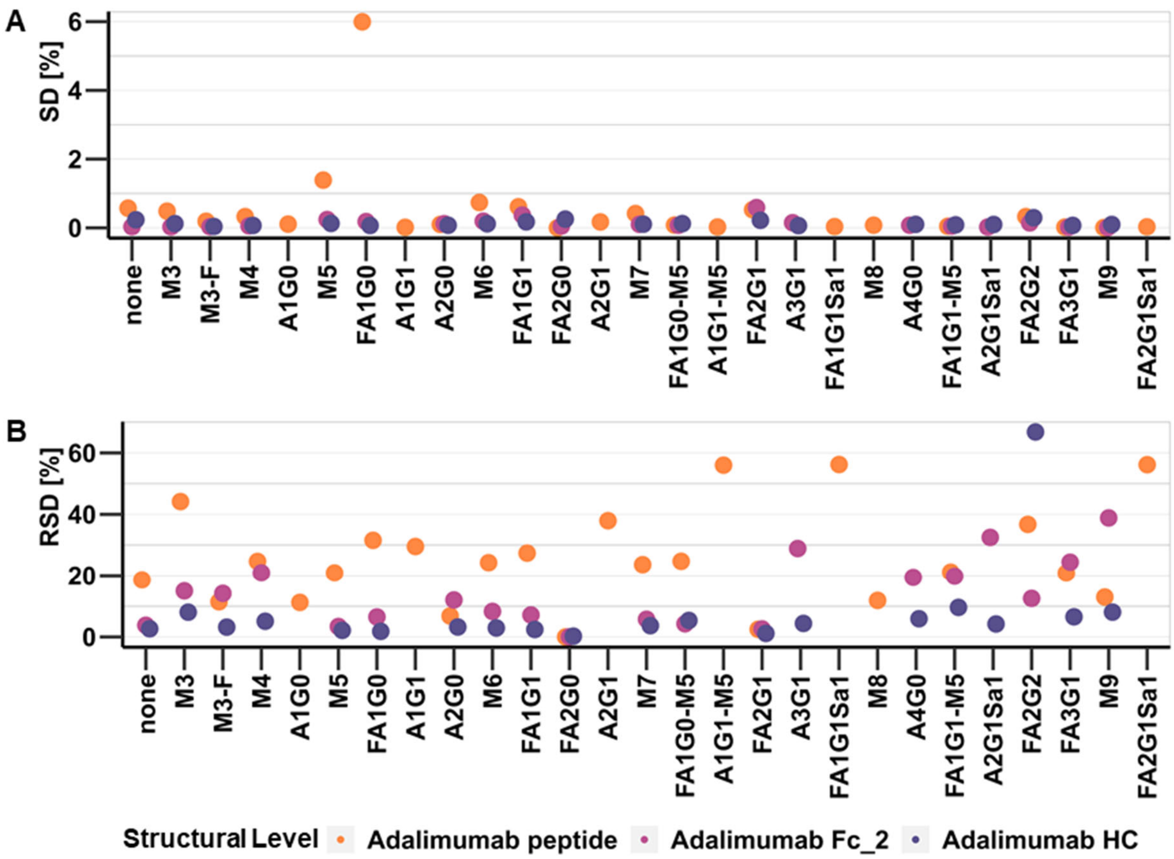

Fig. S 29. Visualization of statistics of adalimumab.
Standard deviation [%] **(A)** and relative standard deviation [%] **(B)** plotted for each glycovariant at different structural levels for adalimumab: peptide, Fc/2 and HC. Standard deviation (SD) and relative SD (RSD) were determined from five technical replicates. Glycan nomenclature is explained in **Fig. S13**.

2. Supplementary tables

Table S 1. Publications considered for meta-data analysis.

Reference number	Author(s)	Year	DOI	mAb	Structural level	Full reference link
Ref1	Zhao *et al.*	2021	10.1021/acs.jproteome.0c00659	NISTmAb	glycopeptide	[3]
Ref2	Carillo *et al.*	2020	10.1016/j.jpha.2019.11.008	Rituximab	intact_native, heavy_chain, Fc_2, glycopeptide	[4]
Ref3	Montacir *et al.*	2017	10.1016/j.jpba.2017.03.029	Rituximab	intact_native, heavy_chain, Fc_2, glycopeptide	[5]
Ref4	Jakes *et al.*	2021	10.1021/acs.analchem.1c02365	Rituximab	intact_native	[6]
Ref5	Zhu *et al.*	2021	10.1021/acs.jproteome.0c00373	Adalimumab	intact_native, Fc_2	[7]
Ref6	Millán-Martín *et al.*	2021	10.1016/j.ejpb.2020.11.006	Adalimumab	intact_native	[8]
Ref7	Duivelshof *et al.*	2021	10.3390/pharmaceutics13111744	Adalimumab	Fc_2	[9]
Ref8	De Leoz *et al.*	2020	10.1074/mcp.RA119.001677	NISTmAb	intact_native, glycopeptide	[10]
Ref9	Chen *et al.*	2018	10.1080/23312009.2018.1480455	NISTmAb	intact_native	[11]
Ref10	Millán-Martín *et al.*	2020	10.1007/s00216-020-02809-z	NISTmAb	glycopeptide	[12]
Ref11	Bi *et al.*	2022	10.1021/acs.jproteome.2c00027	NISTmAb	glycopeptide	[13]
Ref12	Largy *et al.*	2017	10.1016/j.chroma.2017.02.072	Adalimumab	intact_native, Fc_2, glycopeptide	[14]
Ref13	Liu *et al.*	2016	10.1007/s40259-016-0184-3	Adalimumab	intact_native	[15]
Ref14	Li *et al.*	2022	10.1016/j.aca.2021.339232	Adalimumab	glycopeptide	[16]
Ref15	Giorgetti *et al.*	2018	10.1016/j.talanta.2017.09.083	Adalimumab	glycopeptide	[17]
Ref16	Di Marco *et al.*	2021	10.3390/ijms22169072	Rituximab	intact_native	[18]
Ref17	Groves *et al.*	2020	10.1021/jasms.9b00022	NISTmAb	intact_native	[19]
Ref18	Yan *et al.*	2018	10.1021/acs.analchem.8b03773	NISTmAb	intact_native	[20]

Table S 2. Modification setting of Byonic peptide data evaluation.
Modifications were selected based on the artifacts detected at the subunit level analyses (see Supplementary data at the end of this document).

Modifcation	delta mass [Da]	targets	fine control
Gln->pyro-Glu	-17.027	NTerm Q	common1
Carbamidomethyl	57.021	C	fixed
Oxidation	15.995	M, W	rare2
Deamidated	0.984	N	rare1
Dehydrated	-18.011	NTerm C, Protein CTerm N, Protein CTerm Q, D, S, T, Y	rare1
Glu->pyro-Glu	-18.011	NTerm E	common1
Ammonia-loss	-17.027	NTerm C, Protein NTerm S, Protein NTerm T, N	rare1
Cys->Oxoalanine	-17.993	C	rare2
Met->Xle	-17.956	M	rare2
Asp->Pro	-17.974	D	rare2
Arg->Gln	-28.043	R	rare2
Arg->Lys	-28.006	R	rare2
Gln->Val	-28.990	Q	rare2
Met->Cys	-28.031	M	rare2
Val->Ala	-28.031	V	rare2
Met->Ser	-44.008	M	rare2
Phe->Cys	-44.059	F	rare2
Thr->Gly	-44.026	T	rare2
Asp->Ala	-43.990	D	rare2
Xle->Gly	-56.063	I, L	rare2
Arg->Val	-57.033	R	rare2
Asn->Gly	-57.021	N	rare2
Gln->Ala	-57.021	Q	rare2
Lys->Ala	-57.058	K	rare2
Trp->Glu	-57.037	W	rare2
Trp->Lys	-57.984	W	rare2
Arg->Thr	-55.053	R	rare2
Trp->Met	-55.039	W	rare2
Trp->Xle	-72.995	W	rare2
Hex	162.053	K	common2

Table S 3. Features in the Skyline output.

File Name	Peptide	Peptide Modified Sequence	Modified Sequence Full Names	Protein	Best Retention Time	Precursor	Precursor Charge	Total Area MS1	Total Background MS1	Precursor Mz	Precursor Result	Min Start Time	Max End Time

Table S 4. Released glycan data of NISTmAb.
Peaks which remained unassigned were marked with a question mark (?). Several peaks were assigned with multiple possible glycans (glycan_1, glycan_2, glycan_3). The three most abundant peaks are highlighted in bold. Relative abundance was calculated from five replicate xCGE-LIF measurements. Glycan nomenclature is explained in **Fig. S13**.

peak	glycan_1	glycan_2	glycan_3	MS-detectable annotation	rel. abund. [%]	sd rel. abund. [%]	rsd rel. abund [%]
1	Man3[a]			M3	1.16	0.0320	2.76
2	FA1G1Sg1(2,6)[3]			FA1G1Sg1	3.85	0.0539	1.40
3	FMan3			M3-F	0.35	0.0079	2.26
4	A1G0[3]	A1G0[6]	Man5[D3]-A1G1Sg1(2,6)	A1G0 or A1G0 or A1G1Sg1-M5	0.73	0.0223	3.05
5	FA2G1Sg1(2,6)[6]			FA1G1Sg1	0.80	0.0411	5.14
6	FA2G1Sg1(2,6)[3]			FA2G1Sg1	0.78	0.0348	4.46
7	Man5	FA1G0[3]	FA2G2Sg1(2,6)[3]	M5 or FA1G0 or FA2G2Sg1	7.04	0.0627	0.89
8	FA2G2aG1[6]Sg1(2,6)[3]	Man5[D3]-A1G0	Man6-U	FA2G3Sg1 or A1G0-M5	1.57	0.0646	4.11
**9**	**FA2G0**			**FA2G0**	**100.00**	**0.0000**	**0.00**
10	FA1G1[3]			FA1G1	6.81	0.0243	0.36
11	FMan5-A1G0			FA1G0-M5	0.77	0.0266	3.45
**12**	**FA2G1[6]**			**FA2G1**	**75.72**	**0.2914**	**0.38**
**13**	**FA2G1[3]**			**FA2G1**	**24.68**	**0.0662**	**0.27**
14	A2G1aG1[3]	FA1G1aG1[3]		A2G2 or FA1G2	2.62	0.0283	1.08
15	FMan5[D3]-A1G1			FA1G1-M5	0.33	0.0000	0.00
16	FA2G1aG1[6]			FA2G2	3.63	0.0050	0.14
17	FA2G2			FA2G2	17.75	0.1211	0.68
18	FA2G1aG1[3]			FA2G2	0.44	0.0189	4.30
19	?			-	0.55	0.0271	4.93
20	FA2G2aG1[6]			FA2G3	3.60	0.0463	1.29
21	FA2G2aG1[3]			FA2G3	0.76	0.0263	3.46
22	?			-	0.47	0.0189	4.02
23	FA2G2αG2			FA2G4	2.16	0.0103	0.48

Table S 5. Fractional abundances of the glycovariants of the EEQYNSTYR peptide of NISTmAb.
Quantification was done with Skyline; mean and standard deviations (SD) were calculated from five technical replicates. Glycan nomenclature is explained in **Fig. S13**.

glycan [a]	mean [%] [b]	SD [%]
A1G0	0.734	0.016
A1G0FM5	1.026	0.073
A1G0F	10.219	0.227
A1G1	0.068	0.010
A1G1F	4.504	0.105
A1G1FSg1 or A1G1Sa1	0.013	0.001
A1G1FSg1	1.213	0.062
A1G1Sg1M5	0.050	0.006
A2G0	0.102	0.011
A2G0F	33.692	0.899
A2G1	0.179	0.011
A2G1F	34.549	0.689
A2G1FSg1	0.369	0.023
A2G2F	7.852	0.389
A2G2FSg1	0.442	0.021
A2G3F	1.439	0.146
A2G4F	0.560	0.027
A3G1F	0.578	0.058
M3F	0.895	0.025
M5	0.866	0.042
M6	0.037	0.003
M7	0.021	0.002
unmodified	0.592	0.025

[a], annotated glycan.
[b], mean relative abundance [%]
[c], standard deviation of relative abundance [%]

Table S 6. Number of occurrences of unknown modifications.
Occurrences were counted from a final table of modifications, which detected by Byonic in all five technical replicates. Details on the evaluation are described in **chapter 3.2 and 3.3** of the supplementary material.

Protein	-17 Da	-18 Da	-28 Da	-44 Da	-57 Da
NISTmAb	21	47	11	8	16
NISTmAb + PNGase F	21	47	11	11	20
Rituximab	21	47	11	8	16
Rituximab + PNGase F	21	47	11	11	20
Adalimumab	20	47	11	10	18
Adalimumab + PNGase F	22	45	11	6	21

Table S 7. Statistics on NISTmAb Fc/2- and HC-glycans.
Mean (Ø), standard deviation (SD) and relative standard deviation (RSD) of fractional abundances of Fc/2 (left) and HC (right)-related NISTmAb glycans determined from five technical replicates. RSD values below 5% are highlighted in bold. Glycan nomenclature is explained in **Fig. S13**.

	Fc/2			HC		
glycan	Ø frac. ab. [%]	SD [%]	RSD [%]	Ø frac. ab. [%]	SD [%]	RSD [%]
none	0.69	0.07	10.80	4.66	0.04	**0.76**
A1G0	0.25	0.02	7.66	1.00	0.04	**3.73**
FA1G0	0.92	0.05	**5.00**	2.22	0.06	**2.56**
FA1G0-M5	2.11	0.06	**2.65**	3.64	0.09	**2.51**
FA1G1	2.57	0.02	**0.75**	4.48	0.13	**2.84**
FA1G1-M5	0.54	0.04	8.25	1.05	0.05	**4.74**
FA1G1Sg1	0.54	0.05	9.05	1.51	0.04	**2.33**
A1G1Sg1-M5	0.13	0.03	20.64	0.71	0.05	6.59
FA2G0	37.95	0.12	**0.31**	28.83	0.19	**0.67**
FA2G1	39.62	0.15	**0.38**	30.97	0.31	**0.99**
FA2G1Sg1	0.24	0.03	12.58	0.88	0.05	5.67
FA2G2	9.85	0.10	**1.02**	9.78	0.08	**0.81**
FA2G2Sg1	0.26	0.03	11.14	0.74	0.03	**4.03**
FA2G3	1.63	0.09	5.53	2.97	0.05	**1.62**
FA2G3Sg1	0.20	0.02	9.72	0.79	0.04	5.27
FA2G4	0.46	0.07	15.17	1.20	0.05	**4.15**
A3G1	0.86	0.12	14.25	1.06	0.04	**3.84**
M3	0.03	0.01	27.60	0.49	0.03	6.42
M4	0.05	0.01	24.70	0.50	0.04	8.16
M5	0.59	0.07	11.57	0.86	0.05	6.18
M7	0.43	0.04	10.27	0.98	0.06	6.34
M9	0.06	0.01	22.63	0.68	0.04	5.45

Table S 8. Released glycan data for rituximab as determined by xCGE-LIF.
Relative abundance and corresponding standard deviation were determined from five technical replicates. Glycan nomenclature is explained in **Fig. S13**.

glycan	MS-detectable composition	relative abundance [%]	standard deviation [%]
Man3[a]	M3	1.24	0.0253
FA2G2Sa2(2,3)	A2G2FSa2	3.62	0.1236
FA1G1Sa1(2,3)[6]	A1G1FSa1	0.34	0.0181
FA1G1Sa1(2,3)[3]	A1G1FSa1	1.03	0.0242
FA2G1Sa1(2,3)[6]	A2G1FSa1	0.94	0.0204
FA2G1Sa1(2,3)[3]	A2G1FSa1	0.85	0.0097
Man5 or A1BG0[6]	M5 or A2G0	0.44	0.0244
Man5 or FA1G0[3]	A1G0F or M5	2.69	0.0177
A2G0	A2G0	1.93	0.0198
FA2G2Sa1(2,3)[6]	A2G2FSa1	3.17	0.0186
FA2G2Sa1(2,3)[3] or A1G1[6]	A1G1 or A2G2FSa1	2.00	0.0373
FA1BG0[6]	A2G0F	0.27	0.0000
FA2G0 or FA1G1[6]	A2G0F or A1G1	100.00	0.0000
A3[2,6]G0 or A2G1[6]	A3G0 or A2G1	0.68	0.0247
FA1G1[3] or A2G1[3]	A2G1 or A1G1F	1.84	0.0212
Man7[D1] or FMan5-A1G0	M7 or A1G0F-M5	1.45	0.0257
FA2G1[6]	A2G1F	82.36	0.3083
A3[2,4]G1 or FA2G1[3] or A3[2,6]G1	A3G1 or A2G1F	26.24	0.1260
FMan5-A1G1	A1G1F-M5	0.81	0.0138
FA3[2,6]G1	A3G1F	0.65	0.0138
Man9 or FA2G2	M9 or A2G2F	21.71	0.1550

Table S 9. Released glycan data for adalimumab as determined by xCGE-LIF.
Relative abundance and corresponding standard deviation were determined from five technical replicates. Glycan nomenclature is explained in **Fig. S13**.

glycan	MS-detectable composition	relative abundance [%]	standard deviation [%]
Man3[c] or Man3[b]	M3	0.33	0.0112
Man3[a]	M3	1.64	0.0186
FMan3	M3-F	0.51	0.0099
Man4 or A2G1$1(2,3)[3]	M4 or A2G1Sa1	0.36	0.0121
?	-	0.60	0.0130
Man5 or FA1G0[3]	M5 or FA1G0	5.61	0.0172
A2G0	A2G0	0.67	0.0118
Man6	M6	0.90	0.0187
FA2G0 or A1BG1[6] or Man5-A1G0	FA2G0 or A2G1 or M5-A1G0	100.00	0.0000
FA1G1[3] or Man5-Glc	FA1G1 or M6	1.12	0.0210
Man7[D2] or A3[2,4]BG0	M7 or A4G0	0.38	0.0087
Man7[D1] or FMan5-A1G0	M7 or M5-FA1G0	1.25	0.0098
FA2G1[6] or Man5[D3]-A1G1	FA2G1 or M5-A1G1	15.41	0.0368
A3[2,4]G1 or FA2G1[3] or A3[2,6]G1	A3G1 or FA2G1 or A3G1	5.23	0.0149
FA3[2,6]G1 or FMan5-A1G1	FA3G1 or M5-FA1G1	0.28	0.0000
Man9 or FA2G2	M9 or FA2G2	1.36	0.0068

Table S 10. Comparison of experimental means of glycans at the Fc/2 and HC level.
Before performing a t-test, the F-statistic was determined to check whether there is a significant difference between the two variances at the 5% level. If there was no significant difference, i.e., F-statistic < F-critical, a pooled estimate, s, was calculated with which the t-statistic was determined. If F-statistic > F-critical, the t-statistic was calculated including the variances in the formula according to reference [21] (book chapter 3.3 and 3.6). Computations were performed in R (*d_comparison.R*). Glycan nomenclature is explained in **Fig. S13**.

NISTmAb			Adalimumab			Rituximab		
glycan [a]	F-statistic [b]	t-statistic [c]	glycan [a]	F-statistic [b]	t-statistic [c]	glycan [a]	F-statistic [b]	t-statistic [c]
A1G0	9.262	323.43	A2G0	2.582	209.763	A1G1	2.294	331.192
A1G1Sg1-M5	6.374	171.869	FA1G0	7.278	76.015	A2G0	**37.825**	4.45
FA1G0	5.819	197.372	FA1G0-M5	2.647	80.281	A2G1	**15.403**	35.538
FA1G0-M5	2.244	211.313	FA1G1	4.553	37.318	FA1G0	1.054	85.523
FA1G1	**136.756**	33.601	FA1G1-M5	3.156	209.787	FA1G0-M5	5.625	20.873
FA1G1Sg1	1.037	330.183	FA2G0	**24.884**	**0.000**	FA1G1	**18.424**	10.201
FA2G0	1.078	6.115	FA2G1	7.298	29.237	FA1G1-M5	**39.473**	2.383
FA2G1	**26.936**	**2.157**	FA2G2	4.193	20.792	FA1G1Sa1	2.979	456.898
FA2G1Sg1	4.704	196.087	FA3G1	**12.167**	30.336	FA2G0	**24.162**	7.159
FA2G2	2.895	**0.41**	M3	**19.435**	23.815	FA2G1	5.513	**0.000**
FA2G2Sg1	2.291	276.988	M3-F	1.462	1246.193	FA2G1Sa1	1.266	787.393
FA2G3	1.482	26.78	M4	1.526	439.285	FA2G2	2.947	33.101
FA2G4	1.099	77.797	M5	3.496	46.382	FA2G2Sa1	**46.683**	27.977
M5	1.287	52.691	M6	2.75	102.801	FA3G1	3.593	250.783
M7	4.579	89.247	M7	1.08	163.07	M5	6.363	31.977
none	2.307	742.411	M9	**102.172**	27.066	M7	13.6	8.007
			none	**50.742**	75.394	M9	4.899	347.566
						none	3.405	169.876

[a] Glycan, statistical test was performed on glycovariants occurring at all three – peptide, Fc/2 and HC – structural levels.
[b] F-statistic, the critical F-value was determined based on Degrees of Freedom = n – 1 = 4, at alpha = 0.05, F-critical = 9.605.
[c] t-statistic, the critical t-value was determined based on Degrees of Freedom = $n_1 + n_2 - 2 = 8$, at alpha = 0.05, t-critical = 2.31.

3. Supplementary data

3.1. Superiority of the S-trap peptide generation protocol over classical C_{18} ZipTips.

Initial tryptic digestion procedures were conducted following a classical in-solution digestion (ISD) protocol (method details are provided below). However, many carbohydrates identified in the released glycan approach could not be detected in the glycopeptide data. Therefore, we tried an alternative protocol implementing the S-trap. Here, especially minor abundant variants were seen in glycopeptide data, which were missed in measurements of the classical ISD-generated peptides (**Fig. S1**). As shown by Varnavides *et al.* [22], S-trap based tryptic digestion performed better than other proteomics sample preparation workflows when *N*-glycosylated proteins were considered [22]. We therefore only used quantitative peptide data derived from the S-trap protocol for downstream data integration.

Method C_{18} ZipTip sample preparation and HPLC-MS/MS analysis. mAbs were buffer exchanged to 175 mM ammonium acetate using 50 kDa MWCO filters. Next, mAb concentration was determined using NanoDrop at 260 nm and 280 nm. To reduce disulfide bonds, TCEP was added to a final concentration of 5 mmol L^{-1} and allowed to react at 60 °C and 900 rpm for 30 min. Reduction was quenched by addition of iodoacetamide to a final concentration of 20 mmol L^{-1} (22 °C, darkness, 900 rpm, 60 min). Reduced and alkylated polypeptides were purified using C_{18} tips according to manufacturer's instructions eluting them with 0.1% FA in 99% ACN. Samples were evaporated to dryness at 30 °C under vacuum for 30 min and re-dissolved in 175 mmol L^{-1} ammonium acetate. Tryptic digestion followed using an enzyme protein ratio of 1:15. Digestion was performed over night at 37 °C and 900 rpm. Trypsin was inactivated by acidification using 10% FA to a final concentration of 1%. HPLC-grade water with 0.1% FA and ACN with 0.1% FA were mobile phase A and B, respectively. Separations were carried out at 50 °C±2 °C. Injection mode was µL-PickUp. Peptides were separated on the Acclaim PepMap® RSLC (15 cm x 300 µm i.d., 2 µm d_p, 100 Å pore size, C_{18}, Thermo Scientific™). The flow rate was set to 1.2 µL min^{-1}. The column was equilibrated at 1% B for 5 min, followed by a linear gradient up to 30% B within 30 min, followed by a linear gradient up to 60% B within 5 min. Then the column was flushed at 99% B for 5 min and re-equilibrated at 1% B for 10 min. Inject mode was µL-PickUp. MS analysis was conducted on a Q-Exactive™ Pluss mass spectrometer. Eluting peptides were sprayed on a nano-ESI (NSI) source under positive polarity at a spray voltage of 1.5 kV. NSI parameters included 0 sheath, auxiliary, and sweep gas flow rate, capillary temperature of 250 °C, and S-lens RF level of 60. Full scan parameters: scan range 400-3,000 m/z, no fragmentation, resolution of 70,000 at *m/z* 200, 1 microscan, AGC target 3e6, maximum inject time 100 ms.

3.2. Detection of artifact modifications after de-glycosylation at subunit levels.

Heavy chain. NISTmAb's heavy chain is 82.5% un-glycated (fractional abundance), and the corresponding -44 Da mass variant has a fractional abundance of 7.2%. This shift could be attributed to an amino acid substitution (e.g., tyrosine to glycine) or CO_2 loss. Besides multiple artifact peaks, up to two times glycated species were observed (**Fig. S18C-D**).

Fc/2. Glycation assessment was also conducted at the Fc/2 level. Mass-loss species were observed in the mass spectra of rituximab, adalimumab, and NISTmAb after PNGase F treatment and IdeS digestion (**Fig. S18A-B**). These "unknown" modifications constitute approximately 16% of fractional abundances, causing mass losses of -17 Da, -28 Da, -44 Da, -56 Da, and -73 Da. The degree of glycation at the Fc/2 level is lower

compared to the heavy chain, as the Fc/2 subunit has fewer lysines available for potential glycation (18 Lys in Fc/2 *vs*. 35 Lys in the heavy chain of rituximab, for example).

3.3. Assessment of PTMs other than glycosylation at the peptide level.

In addition to a list of *N*-glycans, also other PTMs including glycation, oxidation, deamidation and pyroglutamic acid formation, amino acid substitutions etc. were added to the list of possible modifications for Byonic evaluation (see detailed list in **Table S2**). Based on the discovery of some unknown modifications at the subunit and intact protein level, the list was extended with potential modifications causing mass shifts of -17, -18, -28, -44, and -57 Da. Most of which arising from amino acid substitutions. In order to screen for modifications that are not glycans or the alkylation of cysteines, the Byonic output was filtered extensively using R (*Byonic_evaluation.R*):

(i) only peptides that are contained in all five replicates were considered,
(ii) peptides without any modification were removed,
(iii) glycopeptides bearing an *N*-linked glycan were deleted, and
(iv) the table was cleared from peptides that *only* contained the artificial alkylation of the cysteines (+57 Da) as their modification.

Table S5 lists the number of occurrences of each modification per antibody and the PNGase F treatments. The numbers were similar across all mAbs. For example, the modification -44 Da was counted in ten different peptides in the adalimumab measurement.

4. Instructions for multi-level data-integration workflow

4.1. Overview and organization of data and software tools

Fig. S2 is providing a general overview of the workflow described in detail below. All necessary files are to be downloaded from the electronic supplementary material deposited in Zenodo (https://doi.org/10.5281/zenodo.10455819). Basic programming skills in R and python are recommended for the multi-level data-integration workflow. Installation of all relevant software tools is described in **chapter 4.2**.

For the multi-level data-integration workflow the zipped file “R-scripts_Supplement.rar” is required. It contains three master R-scripts (one for each mAb) as well as one folder for each mAb. Each “mAb-folder” contains sub-folders for different structural levels as well as the R-scripts that are used by the master R-script. On the example of NISTmAb, the folder contains the following subfolders and R-scripts:

- *00_Released_glycan_data*: here the released glycan data are organized. The folder contains an R-script for plotting the released glycan data.
- *01_Skyline_data*: this folder contains the relevant Skyline document (e.g., Skyline_NIST.skyd) and the results of the Skyline glycopeptide evaluation (e.g., glycopeptide_NIST_skyline.csv). Please refer to **chapter 4.3** for a detailed description on the Skyline workflow. Further, there is a subfolder with the Byonic evaluation results.
- ***a_Skyline_peptide.R***: this R-script takes the Skyline-output, and performs data wrangling to calculate the fractional and relative abundances and plots the data. Please refer to **chapter 4.3** for details on the R-script.
- *02_Peptide_results*: this folder contains the results of the ***a_Skyline_peptide.R*** evaluation.

- *03_Raw_data_subunit*: this folder contains two sub-folders, each for a subunit, containing the raw-MS data as .mzML files for PNGase F treated and untreated samples.
- ***b_Fragquaxi_Fc_2.R***: this R-script utilizes the package *fragquaxi* to compute fractional and relative abundances of glycovariants at the Fc/2 level. Details are described in **chapter 4.4**.
- ***c_Fragquaxi_HC.R***: same as for the point above just considering the heavy-chain glycans.
- *04_Subunit_results*: here, the results of the ***b_Fragquaxi_Fc_2.R*** and ***c_Fragquaxi_HC.R*** are saved.
- *05_CAFOG*: This folder contains a folder for each subunit with all relevant files required for the CAFOG workflow including a list of glycans (glycan_library.csv), abundances of glycation (glycation.csv) and glycosylation (glycosylation.csv). Utilization of the CAFOG workflow is described in **chapter 4.5**. The results of the CAFOG workflow are to be found in the results.csv document.
- *06_Comparison*: this folder contains all results of comparative analyses including released glycans, glycopeptide, Fc/2 and heavy chain data.
- ***d_Comparison.R***: utilization of this R-script is mentioned in **chapter 4.6**.
    - First, this R-script re-arranges the CAFOG results.csv and saves it to Fc_2_CAFOG_data.xlsx (as on the example of Fc/2) into the *05_CAFOG* folder.
    - Second, this R-script plots the original and for glycation-corrected abundances and saves them as Fc_2_CAFOG.png (on the example of Fc/2) into the *05_CAFOG* folder.
    - Third, this R-script compares the results of all structural levels and saves the results as NISTmAb_site-specific_comparison.png to the *06_Comparison* folder.
    - Fourth, this R-script compares the errors in abundances at the structural levels and saves the results to the *06_Comparison* folder as NISTmAb_plot_error.png and NISTmAb_plot_rsd.png.
    - Fifth, this R-script generates a Venn diagram for a qualitative comparison of the different structural levels and saves the results as NISTmAb_Venn_Diagram.png into the *06_Comparison* folder.
    - Sixth, this R-script performs statistical tests such as the F-test and t-test to compare accuracies and differences in quantification among structural levels. The results are saved as NISTmAb_statistics_Fc_2_HC.xlsx into the *06_Comparison* folder.
- *07_Raw_data_intact*: this folder contains the raw data of the intact protein measurements as .mzML files.
- ***e_Fragquaxi_intact.R***: this R-script quantifies the glycosylation variants at the intact protein level using the package *fragquaxi*. The results are saved in the *08_Intact_results* folder as NISTmAb_Intact_STAT.txt.
- ***f_Intact_evaluation.R***: this R-script wrangles data of the ***e_Fragquaxi_intact.R*** output and saves them as NISTmAb_Intact_rel_ab.xlsx. Further, relative abundances at the intact protein level are plotted as NISTmAb_glycosylation_intact.png. All results are saved to the *08_Intact_results* folder.
- *08_Intact_results*: this folder contains the results concerning all evaluations from the intact protein level, as mentioned above. Further, it contains a sub-folder with the MoFi

input and output files. The input files include the protein sequence (1_NISTmAb_fasta.fasta), list of modifications (2_modifications.csv), list of quantitative data from the peptide level (3_glycan_library_NIST_S-trap.csv), and list of relative abundances from a deconvoluted spectrum at the intact protein level (4_spectrum_NIST_int_nat_BPF.csv). After the MoFi computation is finished, the results are saved according to glycan composition (MoFi_output_S-trap_composition.csv) and/or glycan structure (MoFi_output_S-trap_structure.csv). Details on the MoFi workflow are to be found in **chapter 4.7**.

- *g_Statistics.R* and *09_Meta_analysis*: this R-script and folder are not relevant to the multi-level data-integration workflow. They solely concern the meta-data analysis with existing literature.
- *monoisotopic_mass_glycans.xlsx*: this excel file contains a list of monoisotopic masses of glycans and is utilized by several R-scripts.

4.2. Installation of required software tools

This chapter outlines the prerequisites and steps required for data evaluation using our workflow. Before you begin, please ensure that you have the following software tools pre-installed on your system:

a. Skyline

- Download and install Skyline from the Skyline website.

b. R-Studio

- Download and install R-Studio from the R-Studio website.
- Open the master R-script for each mAb i.e., *NISTmAb.R*

c. *fragquaxi* R-package

- Install the fragquaxi R-package from GitHub repository:
  - Repository URL: https://github.com/cdl-biosimilars/fragquaxi/
  - Follow the installation instructions provided in the README of the repository.

d. MoFi

- Install MoFi:
  - Option 1: Install MoFi as a GUI following the instructions in the publication 10.1021/acs.analchem.8b00019
  - Option 2: Install MoFi from the GitHub repository:
    - Repository URL: https://github.com/cdl-biosimilars/mofi/

e. CAFOG

- Install CAFOG following the instructions provided in the GitHub repository.
  - Repository URL: https://github.com/cdl-biosimilars/cafog/

4.3. Quantification of glycopeptides

Using Skyline

a. Open Skyline to quantify glycopeptides of interest.

- In order to start quantification of glycopeptides in Skyline, a template has to be created following the steps in the following figures: First of all, Skyline has to be operated in the Proteomics interface (**Fig. S 3**). Briefly, it contains the following steps: peptide settings and modifications (**Fig. S 4**, **Fig. S 5**, **Fig. S 6**, **Fig. S 7**), creation of a report file (**Fig. S 8**), import of data (**Fig. S 9**), quantification of XICs (**Fig. S 10**, **Fig. S 11**), and export of results (**Table S3**).

Exporting Skyline results

b. Generate a report (with columns listed in **Table S3** and **Fig. S 8**).
c. Results from Skyline are exported by clicking *File* → *Report* → *Export*. The report name as defined in **Fig. S 8** is selected. Before exporting, the report can be previewed as well.
d. Export the results as a CSV file with a specific filename format, for example, glycopeptide_ADA_skyline.csv.
e. Save the exported CSV file to the "01_Skyline_data" folder.

Evaluating Skyline Output Using R-Script

f. Use the R-script ***a_Skyline_peptide.R*** to evaluate the Skyline output. The script performs the following critical steps:

    - Loading the Skyline output.
    - Computing the total area for each replicate.
    - Calculating fractional abundances of each replicate, irrespective of charge states (note that each peptide can occur at more than one charge state).
    - Grouping fractional abundances for each modification by summing up charge states.
    - Computing the mean, standard deviation, and relative standard deviation.
    - Extracting glycan names from glycopeptide information.
    - Plotting fractional abundances.

g. The results will be saved to the folder "02_Peptide_results".

4.4. Quantification of glycans at Fc/2 and HC

Conversion of Raw Files to mzML Format

h. Convert raw files to the mzML format.
i. Save the converted mzML files to the "03_Raw_data_subunit" folder, organized into subfolders named "Fc_2", "HC" and "PNGaseF".
j. For data related to PNGaseF treated, de-glycosylated proteins, follow the same procedure.
k. Note: mzML files of mAbs used in this study are already saved to the folder "03_Raw_data_subunit".

Preparing for Fragquaxi Evaluation

l. Before starting with the fragquaxi evaluation, ensure that you have saved your mzML files and fasta files to the "extdata" folder. This folder is generated after installing the *fragquaxi* package and can be found in the R-package source on your system.

Using the *Fragquaxi* R-Package for Quantification

m. Utilize the R-package *Fragquaxi* for quantification, employing the provided R-scripts:

- ***b_Fragquaxi_Fc_2.R*** for quantifying Fc/2 glycans
- ***c_Fragquaxi_HC.R*** for quantifying HC glycans

Fragquaxi Worklfow Overview:

n. The *fragquaxi* workflow encompasses the following essential steps:
   - Loading of mzML files.
   - Defining the mAb sequence, disulfide bonds, glycans, and other post-translational modifications (PTMs).
   - Defining the charge states to be considered for calculation.
   - Plotting raw data and m/z windows for different glycans.
   - Quantification based on retention time in seconds.
   - Calculation of abundances and visualization in a bar chart.
   - Computation of fractional abundances.
   - Computation of mean, standard deviation, and relative standard deviation for all replicates.

4.5. Correction of glycosylation abundances for glycation using the CAFOG algorithm

Determining Abundances of Glycation

a. Calculate the abundances of glycation using PNGaseF treated proteins, as determined in the fragquaxi workflow.
b. Save your results as CSV for the CAFOG workflow.

Performing CAFOG Analysis

c. Utilize the CAFOG algorithm to correct glycosylation abundances for glycation.
   - CAFOG analysis is performed as described in the publication in reference [23].
   - Reference [23] provides an exemplary dataset the get the CAFOG analysis running.

Saving CAFOG Results

d. Save the results of the CAFOG analysis to the "05_CAFOG" as XLSX file.

4.6. Comparison involving glycopeptides, Fc/2- and HC-glycans as well as qualitative comparison including released glycan data

Using R-Script for Comparison

a. Utilize the R-script ***d_Comparison.R*** to perform a comparison that involves glycopeptides, Fc/2- and HC-glycans, as well as qualitative comparison including released glycan data.

Key Steps in the Comparison Script

b. Quantitative comparison of Fc/2 and HC data, with CAFOG correction if applicable, including plotting of results.
c. Incorporation of peptide data into the comparison and plotting.
d. Qualitative comparison using a Venn Diagram to visualize intersection of released glycans, glycopeptide, and subunit data.
e. Saving of the resulting plots in the "06_Comparison" folder.

4.7. Evaluation of glycosylation at the intact protein level

Quantification of Paired Gylcans at the Intact Protein Level

a. Quantify paired glycans at the intact protein level. This process involves the following steps:
    - Deconvolution of spectra, which can be accomplished using software tools such as BioPharma Finder, UniDec, etc. In this study we used zero-charge mass values obtained from BioPharma Finder.
    - Peak annotation with MoFi.
    - Extracted ion current quantification with fragquaxi.
b. For this purpose, you will need the R-scripts:
    - ***e_Fragquaxi_intact.R*** and
    - ***f_Intact_evaluation.R***.

MoFi Input Requirements

c. When using MoFi, make sure you have the following input data ready:
    - Protein sequence in FASTA file format.
    - A list of potential modifications in CSV file format with columns for Checked, Name, Composition, Mass, Min, and Max.
    - Glycan library obtained from peptide mapping in CSV file format with column for Checked, Name, Composition, Sites, and Abundance.
    - Zero-charge spectrum in CSV format with columns for avg_mass and rel_abundance.
d. Refer to **Fig. S13** for the MoFi graphical user interface (GUI) example, proved here using rituximab as an example. Input includes protein sequence, PTM composition, PTM structure, and masses and abundances of protein species (deconvoluted spectrum).

MoFi GUI

e. The MoFi output contains a permutation score for each glycosylation variant associated with each deconvoluted mass. For example, as shown in **Fig. S13**, various glycosylation variants – each with a specific percentage of abundance – may have contributed to the intensity of the most abundant peak.

Converting Raw Files to mzML Format

f. Convert raw file to the mzML format and save them to the "07_Raw_data_intact" folder.
g. Note: mzML files of mAbs used in this study are already saved to the folder "07_Raw_data_intact".

Fragquaxi Evaluation

h. Before starting with the fragquaxi evaluation, ensure that you have saved your mzML files and fasta files to the "extdata" folder (in addition to "07_Raw_data_intact), because the fragquaxi-package will read files from "extdata". This folder is generated after installing the fragquaxi package and can be found in the R-package source on your system.

i. Evaluate the data using fragquaxi by following the same points as described in step 3, using the script ***e_Fragquaxi_intact.R***. Ensure that you define mAb-specificities in the script.
j. Perform plotting of abundances using the R-script ***f_Intact_evaluation.R***.

References

[1] S. Neelamegham, K. Aoki-Kinoshita, E. Bolton, M. Frank, F. Lisacek, T. Lütteke, N. O'Boyle, N.H. Packer, P. Stanley, P. Toukach, A. Varki, R.J. Woods, Updates to the Symbol Nomenclature for Glycans guidelines, Glycobiology. 29 (2019) 620–624. https://doi.org/10.1093/glycob/cwz045.

[2] Symbol Nomenclature for Glycans (SNFG), (n.d.). https://www.ncbi.nlm.nih.gov/glycans/snfg.html (accessed September 7, 2021).

[3] J. Zhao, W. Peng, X. Dong, Y. Mechref, Analysis of NIST Monoclonal Antibody Reference Material Glycosylation Using the LC-MS/MS-Based Glycoproteomic Approach, J. Proteome Res. 20 (2021) 818–830. https://doi.org/10.1021/acs.jproteome.0c00659.

[4] S. Carillo, R. Pérez-Robles, C. Jakes, M. Ribeiro da Silva, S. Millán Martín, A. Farrell, N. Navas, J. Bones, Comparing different domains of analysis for the characterisation of N-glycans on monoclonal antibodies, J. Pharm. Anal. 10 (2020) 23–34. https://doi.org/10.1016/j.jpha.2019.11.008.

[5] O. Montacir, H. Montacir, M. Eravci, A. Springer, S. Hinderlich, A. Saadati, M.K. Parr, Comparability study of Rituximab originator and follow-on biopharmaceutical, J. Pharm. Biomed. Anal. 140 (2017) 239–251. https://doi.org/10.1016/j.jpba.2017.03.029.

[6] C. Jakes, F. Füssl, I. Zaborowska, J. Bones, Rapid Analysis of Biotherapeutics Using Protein A Chromatography Coupled to Orbitrap Mass Spectrometry, Anal. Chem. (2021). https://doi.org/10.1021/acs.analchem.1c02365.

[7] W. Zhu, M. Li, J. Zhang, Integrating Intact Mass Analysis and Middle-Down Mass Spectrometry Approaches to Effectively Characterize Trastuzumab and Adalimumab Structural Heterogeneity, J. Proteome Res. 20 (2021) 270–278. https://doi.org/10.1021/acs.jproteome.0c00373.

[8] S. Millán-Martín, S. Carillo, F. Füssl, J. Sutton, P. Gazis, K. Cook, K. Scheffler, J. Bones, Optimisation of the use of sliding window deconvolution for comprehensive characterisation of trastuzumab and adalimumab charge variants by native high resolution mass spectrometry, Eur. J. Pharm. Biopharm. 158 (2021) 83–95. https://doi.org/10.1016/j.ejpb.2020.11.006.

[9] B.L. Duivelshof, S. Denorme, K. Sandra, X. Liu, A. Beck, M.A. Lauber, D. Guillarme, V. D'atri, Quantitative N-Glycan Profiling of Therapeutic Monoclonal Antibodies Performed by Middle-Up Level HILIC-HRMS Analysis, Pharmaceutics. 13 (2021) 1744. https://doi.org/10.3390/pharmaceutics13111744.

[10] M.L.A. De Leoz, D.L. Duewer, A. Fung, L. Liu, H.K. Yau, O. Potter, G.O. Staples, K. Furuki, R. Frenkel, Y. Hu, Z. Sosic, P. Zhang, F. Altmann, C. Grunwald-Grube, C. Shao, J. Zaia, W. Evers, S. Pengelley, D. Suckau, A. Wiechmann, A. Resemann, W. Jabs, A. Beck, J.W. Froehlich, C. Huang, Y. Li, Y. Liu, S. Sun, Y. Wang, Y. Seo, H.J. An, N.C. Reichardt, J.E. Ruiz, S. Archer-Hartmann, P. Azadi, L. Bell, Z. Lakos, Y. An, J.F. Cipollo, M. Pucic-Bakovic, J. Štambuk, G. Lauc, X. Li, P.G. Wang, A. Bock, R. Hennig, E. Rapp, M. Creskey, T.D. Cyr, M. Nakano, T. Sugiyama, P.K.A. Leung, P. Link-Lenczowski, J. Jaworek, S. Yang, H. Zhang, T. Kelly, S. Klapoetke, R. Cao, J.Y. Kim, H.K. Lee, J.Y. Lee, J.S. Yoo, S.R. Kim, S.K. Suh, N. De Haan, D. Falck, G.S.M. Lageveen-Kammeijer, M. Wuhrer, R.J. Emery, R.P. Kozak, L.P. Liew, L. Royle, P.A. Urbanowicz, N.H. Packer, X. Song, A. Everest-Dass, E. Lattová, S. Cajic, K. Alagesan, D. Kolarich, T. Kasali, V. Lindo, Y. Chen, K. Goswami, B. Gau, R. Amunugama, R.

Jones, C.J.M. Stroop, K. Kato, H. Yagi, S. Kondo, C.T. Yuen, A. Harazono, X. Shi, P.E. Magnelli, B.T. Kasper, L. Mahal, D.J. Harvey, R. O'Flaherty, P.M. Rudd, R. Saldova, E.S. Hecht, D.C. Muddiman, J. Kang, P. Bhoskar, D. Menard, A. Saati, C. Merle, S. Mast, S. Tep, J. Truong, T. Nishikaze, S. Sekiya, A. Shafer, S. Funaoka, M. Toyoda, P. De Vreugd, C. Caron, P. Pradhan, N.C. Tan, Y. Mechref, S. Patil, J.S. Rohrer, R. Chakrabarti, D. Dadke, M. Lahori, C. Zou, C. Cairo, B. Reiz, R.M. Whittal, C.B. Lebrilla, L. Wu, A. Guttman, M. Szigeti, B.G. Kremkow, K.H. Lee, C. Sihlbom, B. Adamczyk, C. Jin, N.G. Karlsson, J. Örnros, G. Larson, J. Nilsson, B. Meyer, A. Wiegandt, E. Komatsu, H. Perreault, E.D. Bodnar, N. Said, Y.N. Francois, E. Leize-Wagner, S. Maier, A. Zeck, A.J.R. Heck, Y. Yang, R. Haselberg, Y.Q. Yu, W. Alley, J.W. Leone, H. Yuan, S.E. Stein, NIST interlaboratory study on glycosylation analysis of monoclonal antibodies: Comparison of results from diverse analytical methods, Mol. Cell. Proteomics. 19 (2020) 11–30. https://doi.org/10.1074/mcp.RA119.001677.

[11] C.-H. Chen, H. Feng, R. Guo, P. Li, A.K.C. Laserna, Y. Ji, B.H. Ng, S.F.Y. Li, S.H. Khan, A. Paulus, S.-M. Chen, A.E. Karger, M. Wenz, D.L. Ferrer, A.F. Huhmer, A. Krupke, Intact NIST monoclonal antibody characterization—Proteoforms, glycoforms—Using CE-MS and CE-LIF, Cogent Chem. 4 (2018) 1480455. https://doi.org/10.1080/23312009.2018.1480455.

[12] S. Millán-Martín, C. Jakes, S. Carillo, T. Buchanan, M. Guender, D.B. Kristensen, T.M. Sloth, M. Ørgaard, K. Cook, J. Bones, Inter-laboratory study of an optimised peptide mapping workflow using automated trypsin digestion for monitoring monoclonal antibody product quality attributes, Anal. Bioanal. Chem. (2020) 1–16. https://doi.org/10.1007/s00216-020-02809-z.

[13] M. Bi, B. Bai, Z. Tian, Structure-Specific N-Glycoproteomics Characterization of NIST Monoclonal Antibody Reference Material 8671, J. Proteome Res. 21 (2022) 1276–1284. https://doi.org/10.1021/acs.jproteome.2c00027.

[14] E. Largy, F. Cantais, G. Van Vyncht, A. Beck, A. Delobel, Orthogonal liquid chromatography–mass spectrometry methods for the comprehensive characterization of therapeutic glycoproteins, from released glycans to intact protein level, J. Chromatogr. A. 1498 (2017) 128–146. https://doi.org/10.1016/j.chroma.2017.02.072.

[15] J. Liu, T. Eris, C. Li, S. Cao, S. Kuhns, Assessing Analytical Similarity of Proposed Amgen Biosimilar ABP 501 to Adalimumab, BioDrugs. 30 (2016) 321–338. https://doi.org/10.1007/s40259-016-0184-3.

[16] M. Li, W. Zhu, H. Zheng, J. Zhang, Efficient HCD-pd-EThcD approach for N-glycan mapping of therapeutic antibodies at intact glycopeptide level, Anal. Chim. Acta. 1189 (2022) 339232. https://doi.org/10.1016/j.aca.2021.339232.

[17] J. Giorgetti, V. D'Atri, J. Canonge, A. Lechner, D. Guillarme, O. Colas, E. Wagner-Rousset, A. Beck, E. Leize-Wagner, Y.N. François, Monoclonal antibody N-glycosylation profiling using capillary electrophoresis – Mass spectrometry: Assessment and method validation, Talanta. 178 (2018) 530–537. https://doi.org/10.1016/j.talanta.2017.09.083.

[18] F. Di Marco, T. Berger, W. Esser-skala, E. Rapp, C. Regl, C.G. Huber, Simultaneous monitoring of monoclonal antibody variants by strong cation-exchange chromatography hyphenated to mass spectrometry to assess quality attributes of rituximab-based biotherapeutics, Int. J. Mol. Sci. 22 (2021). https://doi.org/10.3390/ijms22169072.

[19] K. Groves, A. Cryar, S. Cowen, A.E. Ashcroft, M. Quaglia, Mass Spectrometry

Characterization of Higher Order Structural Changes Associated with the Fc-glycan Structure of the NISTmAb Reference Material, RM 8761, J. Am. Soc. Mass Spectrom. 31 (2020) 553–564. https://doi.org/10.1021/jasms.9b00022.

[20] Y. Yan, A.P. Liu, S. Wang, T.J. Daly, N. Li, Ultrasensitive Characterization of Charge Heterogeneity of Therapeutic Monoclonal Antibodies Using Strong Cation Exchange Chromatography Coupled to Native Mass Spectrometry, Anal. Chem. 90 (2018) 13013–13020. https://doi.org/10.1021/acs.analchem.8b03773.

[21] J.N. Miller, J.C. Miller, Statistics and Chemometrics for Analytical Chemistry, 6th ed., Pearson Education Limited, 2010.

[22] G. Varnavides, M. Madern, D. Anrather, N. Hartl, W. Reiter, M. Hartl, In Search of a Universal Method: A Comparative Survey of Bottom-Up Proteomics Sample Preparation Methods, J. Proteome Res. 21 (2022) 2397–2411. https://doi.org/10.1021/ACS.JPROTEOME.2C00265/SUPPL_FILE/PR2C00265_SI_005.ZIP.

[23] W. Esser-Skala, T. Wohlschlager, C. Regl, C.G. Huber, A Simple Strategy to Eliminate Hexosylation Bias in the Relative Quantification of N-glycosylation in Biopharmaceuticals, Angew. Chemie Int. Ed. (2020) anie.202002147. https://doi.org/10.1002/anie.202002147.

[24] W. Skala, T. Wohlschlager, S. Senn, G.E. Huber, C.G. Huber, MoFi: A Software Tool for Annotating Glycoprotein Mass Spectra by Integrating Hybrid Data from the Intact Protein and Glycopeptide Level, Anal. Chem. 90 (2018) 5728–5736. https://doi.org/10.1021/acs.analchem.8b00019.
